# G-Quadruplexes Are Present in Human Coronaviruses Including SARS-CoV-2

**DOI:** 10.3389/fmicb.2020.567317

**Published:** 2020-10-23

**Authors:** Haoran Cui, Leiliang Zhang

**Affiliations:** ^1^ Institute of Basic Medicine, The First Affiliated Hospital of Shandong First Medical University, Jinan, China; ^2^ Science and Technology Innovation Center, Shandong First Medical University and Shandong Academy of Medical Sciences, Jinan, China

**Keywords:** COVID-19, SARS-CoV-2, SARS-CoV, G-quadruplexes, nsP3

## Abstract

The global coronavirus disease 2019 (COVID-19) pandemic is caused by severe acute respiratory syndrome coronavirus 2 (SARS-CoV-2), which is one of seven human coronaviruses. G-quadruplexes are intrinsic obstacles to genome replication. Whether G-quadruplexes are present in human coronaviruses is unknown. In the current study, we have predicted that all seven human coronaviruses harbor G-quadruplex sequences. Conserved G-quadruplex sequences in SARS-CoV and SARS-CoV-2 were analyzed and verified by circular dichroism (CD) spectroscopy and Thioflavin T fluorescence assay. Similar to SARS-CoV, SARS-CoV-2 encodes an nsP3 protein, which is predicted to associate with G-quadruplexes. Targeting G-quadruplex sequences in the SARS-CoV-2 genome by G-quadruplex ligands could be a new way to conquer COVID-19.

## Introduction

Ongoing coronavirus disease 2019 (COVID-19) pandemic has been a major global threat for human health (Lu and Zhang, 2020; Tu et al., 2020), with over 20 million confirmed cases in over 200 countries and regions. COVID-19 is caused by severe acute respiratory syndrome coronavirus 2 (SARS-CoV-2), a betacoronavirus genus of *Coronaviridae* family. Among seven types of *Coronaviridae* family of viruses which could infect humans, HCoV-229E, HCoV-HKU1, HCoV-NL63, and HCoV-OC43 are common around the world, but SARS-CoV, MERS-CoV, and SARS-CoV-2 are more recent and rare.

**Figure 1:**
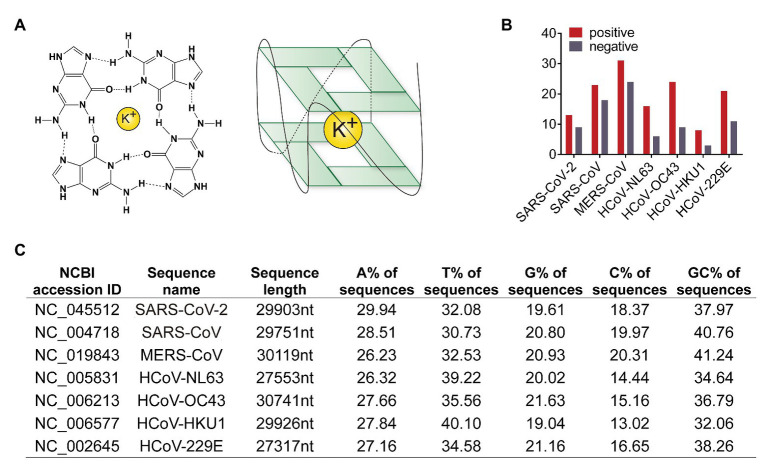
Human coronaviruses genomic RNA possesses G-quadruplexes. **(A)** Schematic diagram of G-quadruplex structure. Guanines forming G-tetrad structure with a potassium ion inside for stable. **(B)** Numbers of predicted G-quadruplex forming sites in seven human coronaviruses. The red column represents the G-quadruplex numbers in positive strand, and the gray showed the number in negative strand. **(C)** Sequence features of human coronavirus genomes. Lane one to three, NCBI accession ID, names, and sequence length of genome reference sequences. Lane four to seven, the content of each base in genome sequences. Lane eight, GC content in genome sequences.

Both SARS-CoV-2 and SARS-CoV are human SARS-related coronavirus (SARSr-CoVs). SARSr-CoVs have positive-stranded RNA genomes of about 30 kb in length, which encodes multiple proteins. One of the most complex tasks for all viruses is to replicate the entire genome. The replication rate of SARS-CoV-2 is higher than SARS-CoV (Chu et al., 2020). However, the underlying mechanism is not clear. There are intrinsic obstacles to genome replication. For instance, the folding of G-rich sequences into G-quadruplex structures is one source of replication stress. G4 structures’ formation requires at least four or more contiguous runs of guanosine nucleotides exist in a short sequence. G-tetrads are formed around K^+^ ions through four Hoogsteen-type hydrogen bonds, and then the tetrads stack to adopt G4 structures (Figure 1A).

Recently, the important functions for virus G4 structures have been demonstrated. G4 structures in the long terminal repeat promoter of the human immunodeficiency virus (HIV) was critical for promoter activity (Amrane et al., 2014; Perrone et al., 2014). A G4 structure in Epstein-Barr virus (EBV) functioned as a *cis*-acting regulatory region to translate EBV encoded nuclear antigen 1 (EBNA1) mRNA (Murat et al., 2014). G4 structures have also been observed in human papillomavirus (HPV), hepatitis B virus (HBV), Nipah virus, hepatitis C virus (HCV), Zika virus, and Ebola virus (Ruggiero and Richter, 2020). These studies highlight a critical role for G4 structures in viruses.

Whether G4 structures are present in the genomes of human coronaviruses including SARS-CoV-2 is largely unknown. To address this question, we analyzed human coronavirus RNA genomes and predicted several conserved G4. G4 ligands could be developed as antiviral agents for human coronaviruses, including SARS-CoV-2, causing the current COVID-19 pandemic. Moreover, we identified SARS-CoV-2 contains less G4 than SARS-CoV, which partially explains why SARS-CoV-2 replicated faster than SARS-CoV-2.

## Materials and Methods

### Sequence Analysis

Genomes of bat SARSr-CoV, SARS-CoV, and SARS-CoV-2 strains were downloaded from the NCBI virus database. Potential G4 forming sequences in human coronavirus genomes were predicted by QGRS mapper^1^ (Kikin et al., 2006) and Quadbase2^2^ (Parashar and Shantanu, 2016). The parameters were as below: max. length, 30; min. G-group size, 2; and loop size from 0 to 12. Global genome alignment among human coronavirus was conducted using R package DECIPHER with default parameters (Wright, 2015). The results were exported in Fasta format and visualized in MEGA X. Sequence logos were generated by weblogo tools^3^ (Crooks et al., 2004).

### Thioflavin T Fluorescence Assay

Single strand RNA oligmers of conserved G4 sequences in SARS-CoV-2, as well as mutant sequences, were synthesized. The sequences were listed in Table 1. RNA oligmers were desolved in RNase free buffer containing 20 mM Tris-HCl and 40 mM KCl to a final concentration of 2 μM. Then the RNA solution was heated at 90°C for 5 min and slowly cooling down to room temperature. Thioflavin T (ThT) powder were bought from Aladdin Industrial Corporation and dissolved in the buffer above. Oligmers and ThT were mixed at final concentration 2 and 2 μM, respectively. The fluorescence at 495 nm emission was collected after 425 nm excitation using SpectraMax microplate reader.

**Table 1:** Sequences for ThT staining and CD spectrum.

Name	G-quadruplex or mutant sequence
nsP1-a	5'-UGGCUUUGGAGACUCCGUGGAGGAGGU-3'
nsP1-b	5'-CGGUAAUAAAGGAGCUGGUGGC-3'
nsP10	5'-CGGUAUGUGGAAAGGUUAUGGC-3'
nsP10-m1	5'-CGGUAUGUAGAAAGGUUAUGGC-3'
nsP10-m2	5'-CGGUAUGUAAAAAGGUUAUGGC-3'
S-a	5'-UGGUUGGACCUUUGGUGCAGGU-3'
S-a-m1	5'-UGGUUAGACCUUUGGUGCAGGU-3'
S-a-m2	5'-UGGUUAAACCUUUGGUGCAGGU-3'
S-b	5'-UGGCUUAUAGGUUUAAUGGUAUUGGA-3'
S-c	5'-UGGCCAUGGUACAUUUGGCUAGGU-3'
N	5'-GGGCUGGCAAUGGCGGA-3'
N-m1	5'-GGGCUAGCAAUGGCGGA-3'
N-m2	5'-GGGCUAACAAUGGCGGA-3'
Positive	5'-AGGGCGGUGUGGGAAGAGGGAAGAGGGGGAGGCAG-3'
Negative	5'-GCGCGCGCUUUUGCGCGCGC-3'

### Circular Dichroism Spectrum

G4 RNA oligmers were dissolved in RNase free buffer containing 20 mM Tris-HCl and 40 mM KCl to a final concentration of 2 μM. Circular dichroism (CD) spectrum was collected by Chirascan V100 from wavelength 200 to 400 nm.

### Plasmids Construction and Primer Extension Assay

G4 sequences were inserted after start codon of green fluorescent protein (GFP) sequences and cloned into pCHA vector. Primers used for plasmid construction were below: forward primer for nsP10, 5'-CCGGAATTCATGGGTATGTGGAAAGGTTATGGCGTGAGCAAGGGCGCC-3'; forward primer for Sa, 5'-CCGGAATTCATGGGTTGGACCTTTGGTGCAGGTGTGAGCAAGGGCGCC-3'; forward primer for N, 5'-CCGGAATTCATGGGCTGGCAATGGCGGGTGAGCAAGGGCGCC-3'; and reverse primer for nsP10, Sa, and N, 5'-CGCGGATCCTCACTTGTACAGCTCATCCAT-3'. Primers used in primer extension assay were below: forward primer, 5'-GTGGAGCAATAGCAGAGCTC-3' and reverse primer, TCACTTGTACAGCTCATCCA-3'. These plasmids were used as PCR templates. N,N΄-(9-(4-(Dimethylamino)phenylamino)acridine-3,6-diyl)bis(3-(pyrrolidin-1-yl)propanamide) (BRACO-19) and meso-5,10,15,20-Tetrakis-(N-methyl-4-pyridyl)porphine, Tetratosylate (TMPyP4) at concentrations of 0, 5, 10, 20, and 40 μM were added into PCR reaction mixture. The PCR products were analyzed by electrophoresis in agarose gels stained with Gelred.

### Protein Expression Assay

G4-based plasmids expressing GFP constructed above were transfected into 293T cells using PEI transfection reagent. Forty micromolar BRACO-19 and TMPyP4 were added into cells 4 h after transfection and the cell lysis and were collected to detect the expression level of GFP by SDS-PAGE followed by Western blot.

### Structure Analysis

The 3D structure of the SARS-CoV-2 nsP3 SARS-unique domain (SUD) was obtained through homology modeling using the swiss-model^4^ (Waterhouse et al., 2018). The template was downloaded from the PDB database with PDB ID 2W2G (Tan et al., 2009). The superimposed image and atom distances were generated by chimera software (Downloaded from http://www.rbvi.ucsf.edu/chimera; Pettersen et al., 2004).

### G4 Ligands

The previously reported G4 ligands with antiviral function were BRACO-19 (Read et al., 1999), PHENdc3 (De Cian et al., 2007), IZCZ-3 (Hu et al., 2018), PIPER (Fedoroff et al., 1998), PDP (Müller et al., 2010), PDS (Rodriguez et al., 2008), TMPyP4 (Parkinson et al., 2007), c-exNDI (Collie et al., 2012), and quarfloxin (Drygin et al., 2009). The chemical structures were drawn using Chemdraw software.

## Results

### Prediction of G4 Sequences in Human Coronaviruses

Genome sequences of MERS-CoV, SARS-CoV, SARS-CoV-2, HCoV-NL63, HCoV-229E, HCoV-OC43, and HCoV-HKU1 were obtained from the NCBI nucleotide database. Genomes of bat SARSr-CoV as well as SARS-CoV and SARS-CoV-2 strains were downloaded from the NCBI virus database. Potential G4 forming sequences in human coronavirus genomes were predicted by QGRS mapper (Kikin et al., 2006). In both positive and negative strands, SARS-CoV-2 possessed less number of predicated G4 and less GC content than SARS-CoV (Figures 1B,C and Supplementary Tables S1 and S2). SARS-CoV-2 with less G4 sequences replicates faster because of less energy is required to bypass G4 structure.

Global genome alignment among human coronaviruses was conducted and conserved G4 forming sites were analyzed according to the corresponding position in genomes and confirmed manually (Supplementary Table S3). Further, another G4 prediction tool quadbase2 was used to confirm the conserved sequences, and the results were listed in Supplementary Table S4. As shown in Supplementary Table S3, the G4 sequences in ORF1ab were conserved in four coronaviruses, which were SARS-CoV, SARS-CoV-2, HCoV-OC43, and HCoV-229E. The G4 sequences in the S protein-coding region were conserved in five coronaviruses (SARS-CoV, SARS-CoV-2, MERS-CoV, HCoV-NL63, and HCoV-229E), including the three with highest mortality rates. G4 sequences are key RNA secondary structures in the viral genomes. Consequently, these conserved G4 sequences could be interesting targets in developing of innovative drugs against human coronaviruses.

**Figure 2:**
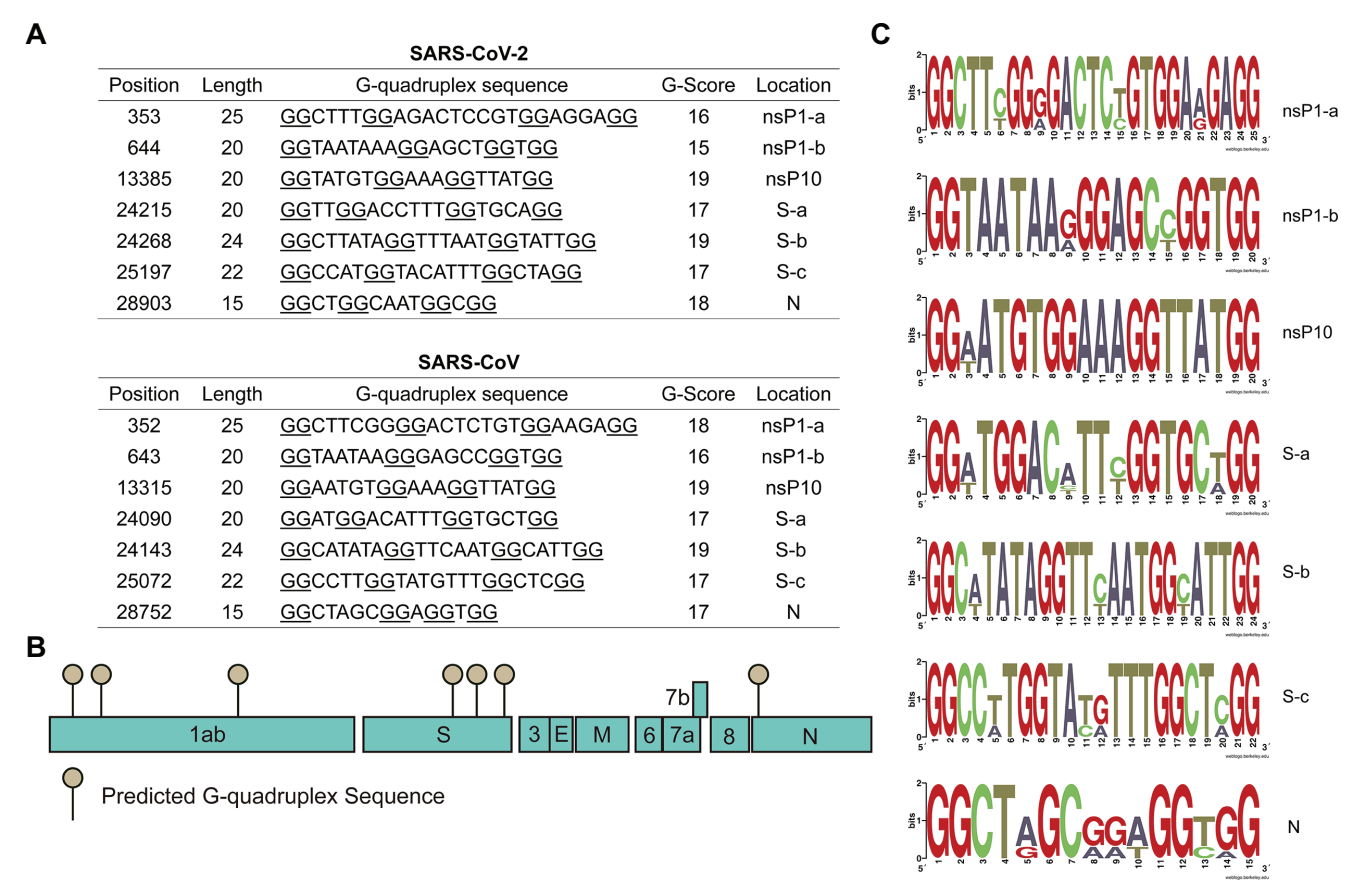
Severe acute respiratory syndrome-related coronavirus (SARSr-CoV) possesses G-quadruplexes. **(A)** Conserved G-quadruplex sequences in severe acute respiratory syndrome coronavirus 2 (SARS-CoV-2) and SARS-CoV genomes. **(B)** A model of the conserved G-quadruplex forming sites in SARS-CoV-2 genome. **(C)** Sequence logos formed by G-quadruplex sequences in typical SARSr-CoV.

Interestingly, SARS-CoV and SARS-CoV-2 shared the most similar G4 sequences among human coronaviruses. Seven conserved G4 sequences in SARS-CoV and SARS-CoV-2 genomes were listed in Figure 2A, and the schematic diagram of G4 sites were shown in Figure 2B. There were two in the nsP1 coding region and three in S coding region, and the other two were in nsP10 and N coding regions. According to the SARS-CoV-2 genome annotation, we identified the conserved position of G4 sequences (Figure 2A).

Conserved G4 sequences in SARSr-CoV were analyzed, including bat SARS-related coronavirus, SARS-CoV, and SARS-CoV-2. The results showed that the seven G4 sites identified above were also conserved in bat SARSr-CoV (Supplementary Tables S5 and S6). To better visualize the conserved sequences, sequence logos were generated using the weblogo tool. Results in Figure 2C showed that critical G4 sequences were conserved in typical SARSr-CoV. To further explore G4 sequences’ evolution in SARS-CoV-2, all the SARS-CoV-2 strains available on the NCBI database (up to 8th April 2020) from different countries were downloaded and analyzed. Genome alignment was conducted using R package DECIPHER, and it turned out that G4 sites were highly conserved in SARS-CoV-2 strains (Supplementary Table S7 and Supplementary Figure S1). Finally, the conserved G4 sequences in all SARSr-CoV strains were aligned and logos were generated (Supplementary Figure S2), indicating that the G4 sequences were evolutionarily conserved. Our observation of the strong conservation of the G4 sites in SARSr-CoV genomes supports a hypothesis that these sequences are very important for SARSr-CoV.

### Characterization of G-Quadruplex Structures

**Figure 3:**
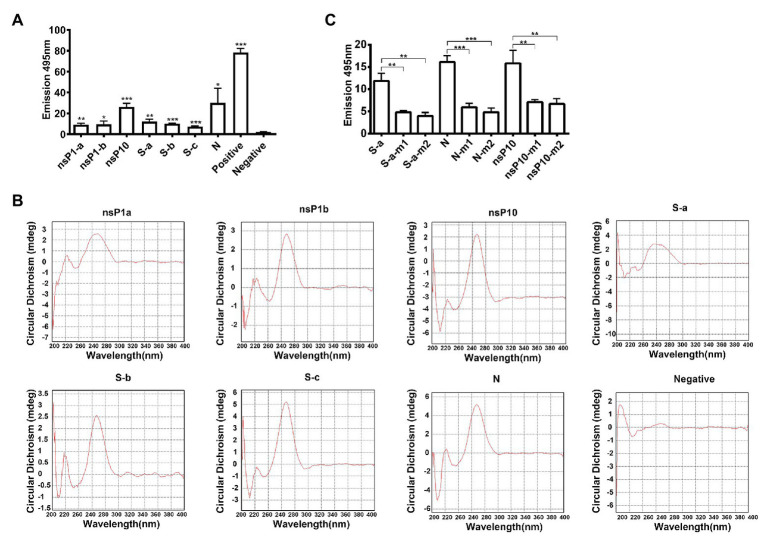
Characterization of G-quadruplex structures. **(A)** Thioflavin T (ThT) fluorescence assay for each conserved G-quadruplex sequences. ^*^p < 0.05, ^**^p < 0.01, and ^***^p < 0.001 based on the Student *t*-test. All results are from three independent experiments. Error bars, SD. **(B)** Circular dichroism (CD) spectrum of conserved G-quadruplex sequences. **(C)** ThT fluorescence assay for G-quadruplex single or double mutations. m1, single guanine mutation; m2, double guanines mutation. ^*^p < 0.05, ^**^p < 0.01, and ^***^p < 0.001 based on the Student *t*-test. All results are from three independent experiments. Error bars, SD.

G4 structure-specific binding to ThT to induce its fluorescence (Renaud de la Faverie et al., 2014). Single strand RNA oligomers of these conserved sequences in SARS-CoV-2 were synthesized. ThT fluorescence assay results showed that these conserved sequences were adopted to form G4 structures in SARS-CoV-2 genomes (Figure 3A).

Circular dichroism spectrum was employed to further confirm the existence of G4 structure in SARS-CoV-2 genome. All these seven conserved sequences have absorbance at about 264 nm, indicating that G4 structures in SARS-CoV-2 genome were adopted to form parallel-strand topologies (Figure 3B). To analyze the effect of single guanine in the G4 region, single or two nucleotide mutations of nsP10, S-a, and N G4 sequences were designed and oligomers were synthesized. The results in Figure 3C showed that the fluorescence signals of mutation oligomers were decreased significantly compared with that of wide type.

### SARS-CoV-2 nsP3 Potentially Associates With G-Quadruplex Sequences

**Figure 4:**
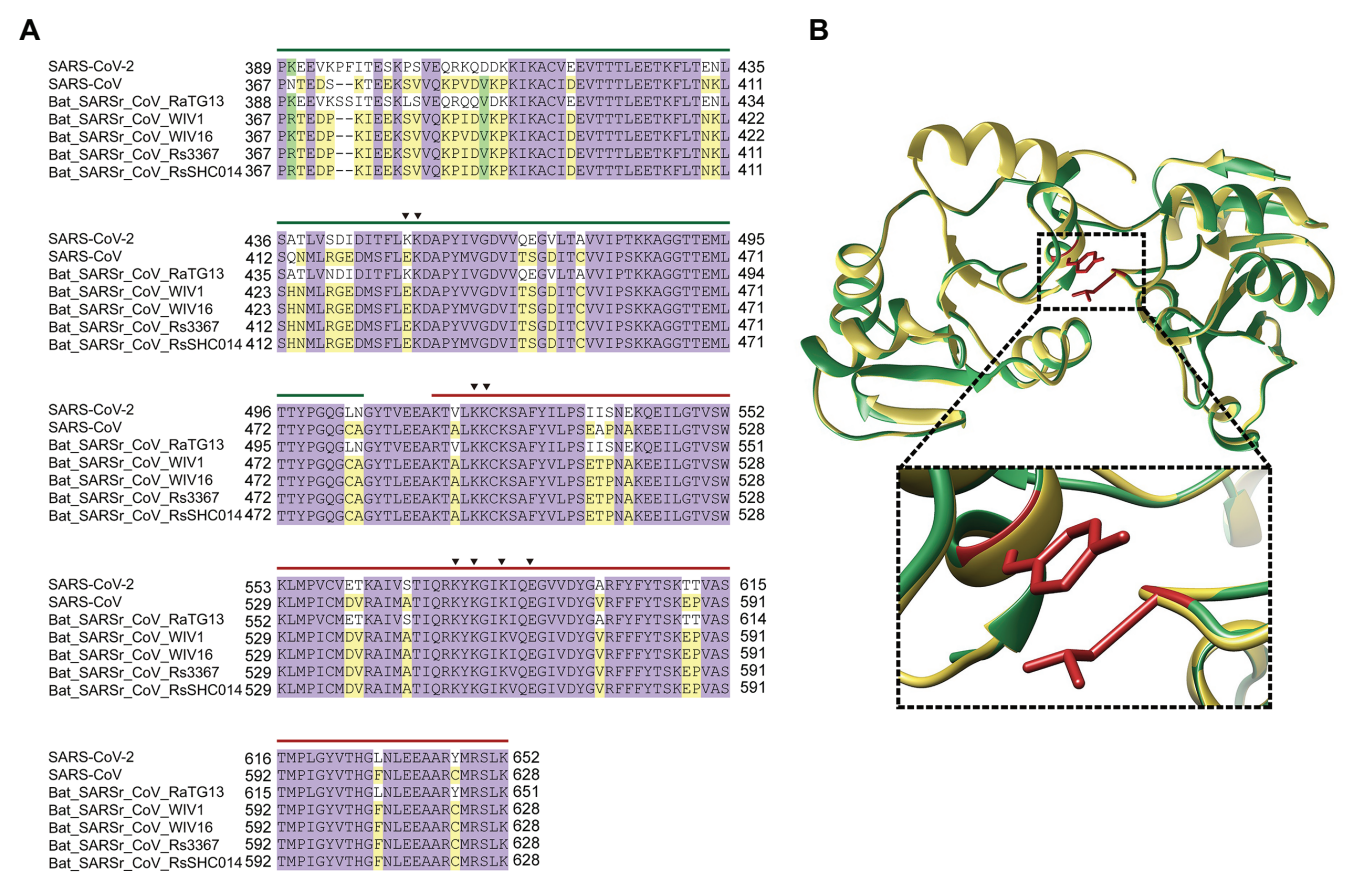
SARS-CoV-2 nsP3 potentially associates with G-quadruplex sequences. **(A)** Sequence alignment of nsP3 SARS-unique domain (SUD)-M and SUD-N in typical SARSr-CoV. The key amino acids in G-quadruplex binding were indicated in triangle. Green line is SUD-M, while red line is SUD-N. **(B)** SUD 3D structure superimpose of SARS-CoV and SARS-CoV-2. The enlarged image showed the atom distance between L516 and Y647 in SARS-CoV-2.

SARSr-CoV encodes an nsP3 protein, which possesses two SUD (M and N) capable of interacting with G4 sequences and potentially essential for unwinding G4 folds in RNA (Figure 4A; Kusov et al., 2015). Stimulated structure of SUD from SARS-CoV-2 nsP3 showed a similar structure to SARS-CoV SUD. Interestingly, an L-Y hydrophobic interaction in SARS-CoV-2 SUD replaced the disulfide bond in SARS-CoV SUD (Figure 4B). The two SUD from SARS-CoV-2 and SARS-CoV are conserved, indicating the co-evolution of nsP3 and G4 sequences (Supplementary Figure S3). The utility of SUD adds additional support for the importance of these genomic G4 folds in SARSr-CoV.

### G-Quadruplex Ligands Block G4-Based Gene Expression

**Figure 5:**
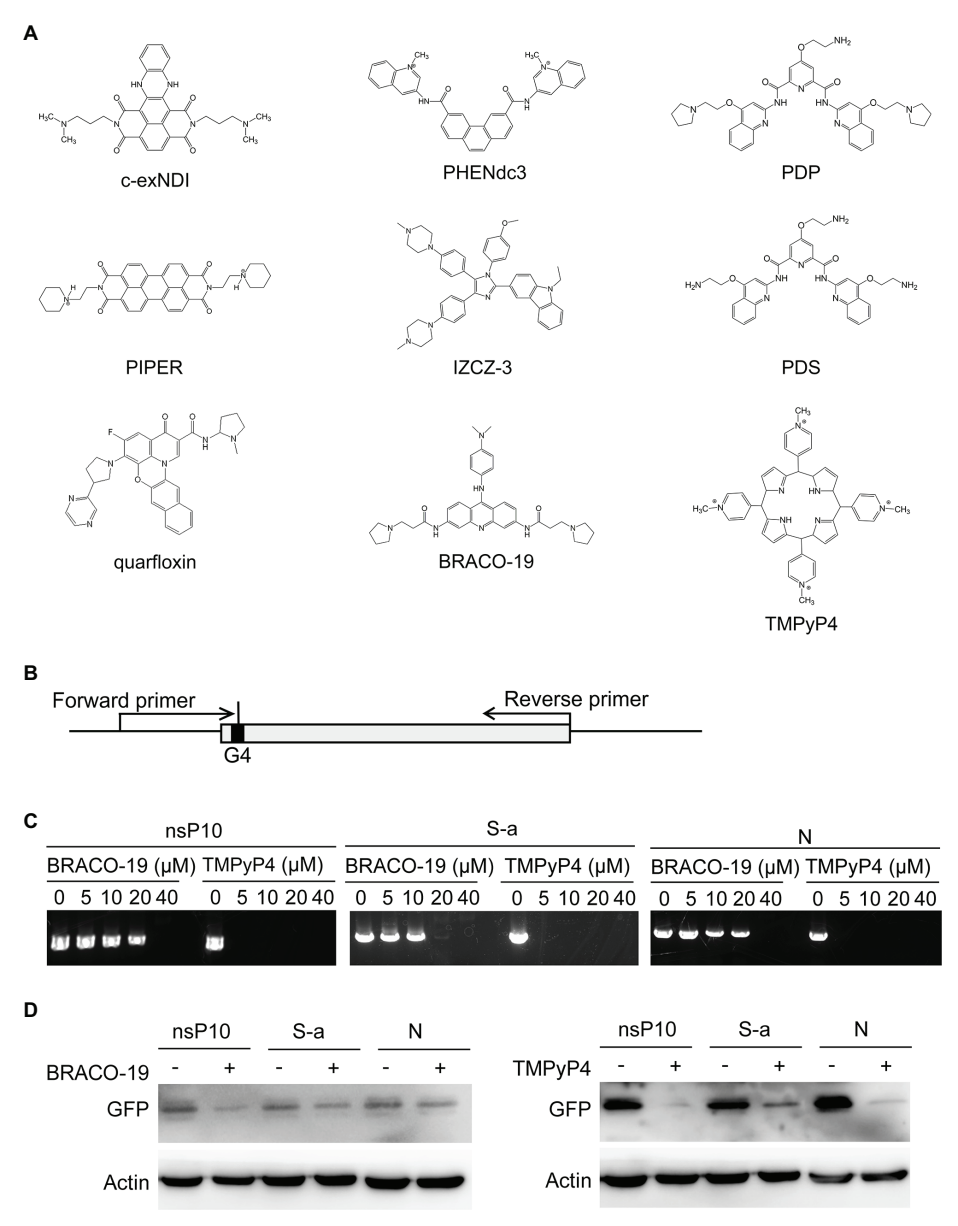
G-quadruplex ligands BRACO-19 and TMPyP4 block G4-based gene expression. **(A)** G-quadruplex ligands with antiviral roles. **(B)** Schematic diagram of G-quadruplex containing plasmids construction and primer extension strategies. **(C)** PCR products of primer extension assay were observed by agarose electrophoresis. The concentrations of G-quadruplex ligands treatment were as indicated. **(D)** GFP expression level with or without treatment were detected through Western blot.

G4 ligands could stabilize RNA G4 and have been demonstrated to be potential antivirus strategies for HIV, HBV, HCV, and Ebola virus (Ruggiero and Richter, 2018). Figure 5A listed the G4 ligands which have been reported to exert antiviral activities in the past few years. G4 sequences were present in human coronaviruses, and these G4 ligands may be developed as potential drugs against SARS-CoV-2, SARS-CoV, and MERS-CoV.

Primer extension assay were performed to detect whether G4 stabilization affect DNA replication. Plasmids were constructed by inserting the G4 sequences into GFP gene after translation start codon ATG, and two primers were selected to amplify the GFP gene (Figure 5B). Two G4 binding ligands, BRACO-19 and TMPyP4, were used to stabilize the G4 structure. As shown in Figure 5C, with the increase of treatment concentration, the yield of PCR product reduced. To further detect the influence of G4 structure in protein expression, plasmids constructed above were transfected into cells and GFP expression were examined in the presence or absence of G4 ligands. The results showed both BRACO-19 and TMPyP4 treatment decreased the expression of GFP inserted by G4 sequences (Figure 5D).

## Discussion

Our study provides a paradigm for assessing G4 functions in viral genomes. Through computational search, different G4-forming sequences were predicted from the human coronaviruses including SARS-CoV-2. The formation of G4s was determined by CD spectroscopy and ThT fluorescence assay. The role of a G4 in gene expression was addressed using primer extension assay and Western blot. Overall, our results point to a potential role for G4s in controlling SARS-CoV-2 viral gene expression. The role of G4s in SARS-CoV-2 viral replication awaits further investigation. We believe that genome-wide analyses of G4s in more viruses will help us to establish a general link between virus life cycle and viral G4s.

G4 structures could hinder gene expression. SARS-CoV-2 contains fewer predicated G4 than SARS-CoV (Figure 1B), which partially explains why SARS-CoV-2 replicates faster than SARS-CoV. G4 sequences are potential antiviral targets. We showed G4 ligands including TMPyP4 and BRACO-19 could inhibit G4 reporter expression (Figure 5D), indicating that G4 ligands could inhibit G4-contaning virus genome replication.

SARS-CoV-2 G4 could be used to develop tools for SARS-CoV-2 studies. G4 has been applied to detect HCV (Luo et al., 2019) and HIV (Sontakke and Srivatsan, 2020). Likewise, G4 could be developed as a potential biosensor for SARS-CoV-2 detection. G4 is applied to set up HCV helicase assay (Leung et al., 2015). In the future, G4 might be used to measure SARS-CoV-2 nsP13 helicase assay.

Many viral proteins associated with virus-encoded G4. HIV-1 nucleocapsid protein NCp7 binds and unfolds the HIV-1 G4 and promotes reverse transcription (Butovskaya et al., 2019). HCV helicase NS3 unwound viral G4 (Leung et al., 2015). SUD domains of SARS-CoV nsP3 were shown to bind to viral G4 (Tan et al., 2009) and play a critical role in viral replication and transcription (Kusov et al., 2015). Based on the similarity of SARS-CoV SUD and SARS-CoV-2 SUD (Figure 4B), nsP3 from SARS-CoV-2 was predicted to associate with viral G4 through SUD domain. Moreover, it is possible that SARS-CoV-2 helicase nsP13 may unwind viral G4 to enhance viral replication.

Viral G4 also associated with host proteins. Cellular nucleolin interacted with viral core G4 to suppress HCV replication (Bian et al., 2019). Nucleolin directly binds to EBV G4 in EBNV1 mRNA sequence to inhibit EBNV1 protein expression (Lista et al., 2017). Nucleolin stabilizes the HIV-1 LTR G4s, and the human ribonucleoprotein A2B1 (HnRNP A2/B1) unwinds the G4s to promote HIV-1 transcription (Scalabrin et al., 2017). Whether host proteins interact with G4 of SARS-CoV-2 remains inconclusive. In the future, we will identify host proteins involved in the function of SARS-CoV-2 G4.

In summary, our results have predicted that all seven human coronaviruses harbor G4 sequences, indicating that G4 structures are crucial elements in the genomes of human coronaviruses. Thus, targeting G4 in viral genomes is a new way to develop antiviral agents. Analysis through genome alignment demonstrated that SARS-CoV and SARS-CoV-2 contained seven conserved G4 sequences. ThT fluorescence assay and CD spectroscopy showed that these conserved G4 sequences in SARS-CoV-2 were able to form G4 folds. Whether these G4s are important for maintaining global genome structure remains an open question. Further studies are needed to better understand these G4s in human coronaviruses and, more specifically, the SARS-CoV-2.

## Data Availability Statement

All datasets presented in this study are included in the article/Supplementary Material.

## Author Contributions

HC performed the bioinformatics study and experiments. LZ conceived the research. HC and LZ wrote the manuscript and approved the final version for publication.

### Conflict of Interest

The authors declare that the research was conducted in the absence of any commercial or financial relationships that could be construed as a potential conflict of interest.

## Footnotes

^1^http://bioinformatics.ramapo.edu/QGRS/index.php/analyze.php

^2^http://quadbase.igib.res.in/TetraPlexFinder

^3^https://weblogo.berkeley.edu/logo.cgi

^4^https://swissmodel.expasy.org/
