# Supplementary material for: G-Quadruplexes Are Present in Human Coronaviruses Including SARS-CoV-2
Source: Front Microbiol. 2020 Oct 23;11:567317. doi: 10.3389/fmicb.2020.567317 (PMC7644843; doi:10.3389/fmicb.2020.567317)
Supplement: Supplementary file 1 [file Table_1.docx]

**G-quadruplexes are present in human coronaviruses including SARS-CoV-2**

Haoran Cui, Leiliang Zhang^*^

**Supporting Information**

**Figure S1** Conserved G-quadruplex sequence logos formed by SARS-CoV-2 strains.

**Figure S2** Conserved G-quadruplex sequence logos formed by SARSr-CoV.

**Figure S3** Amino acid alignment of nsP3 SARS unique domain in human coronaviruses.

**Table S1** Predicted G-quadruplex sequences in seven human coronaviruses.

**Table S2** Predicted G-quadruplex sequences in negative strands of seven human coronaviruses.

**Table S3** Conservation analysis on G-quadruplex sequences in human coronaviruses.

**Table S4** Results comparison of two prediction website in human coronaviruses. This table showed G-quadruplex sequences predicted by Quadbase2 tool. The lines in yellow indicated the consistent results of conserved sequences with QGRS mapper.

**Table S5** Conserved G-quadruplxe sequences in bat SARSr-CoV.

**Table S6** Conserved G-quadruplex sequences in typical SARSr-CoV.

**Table S7** Conserved G-quadruplex sequences in SARS-CoV-2 strains.

**Figure S1** Conserved G-quadruplex sequence logos formed by SARS-CoV-2 strains.


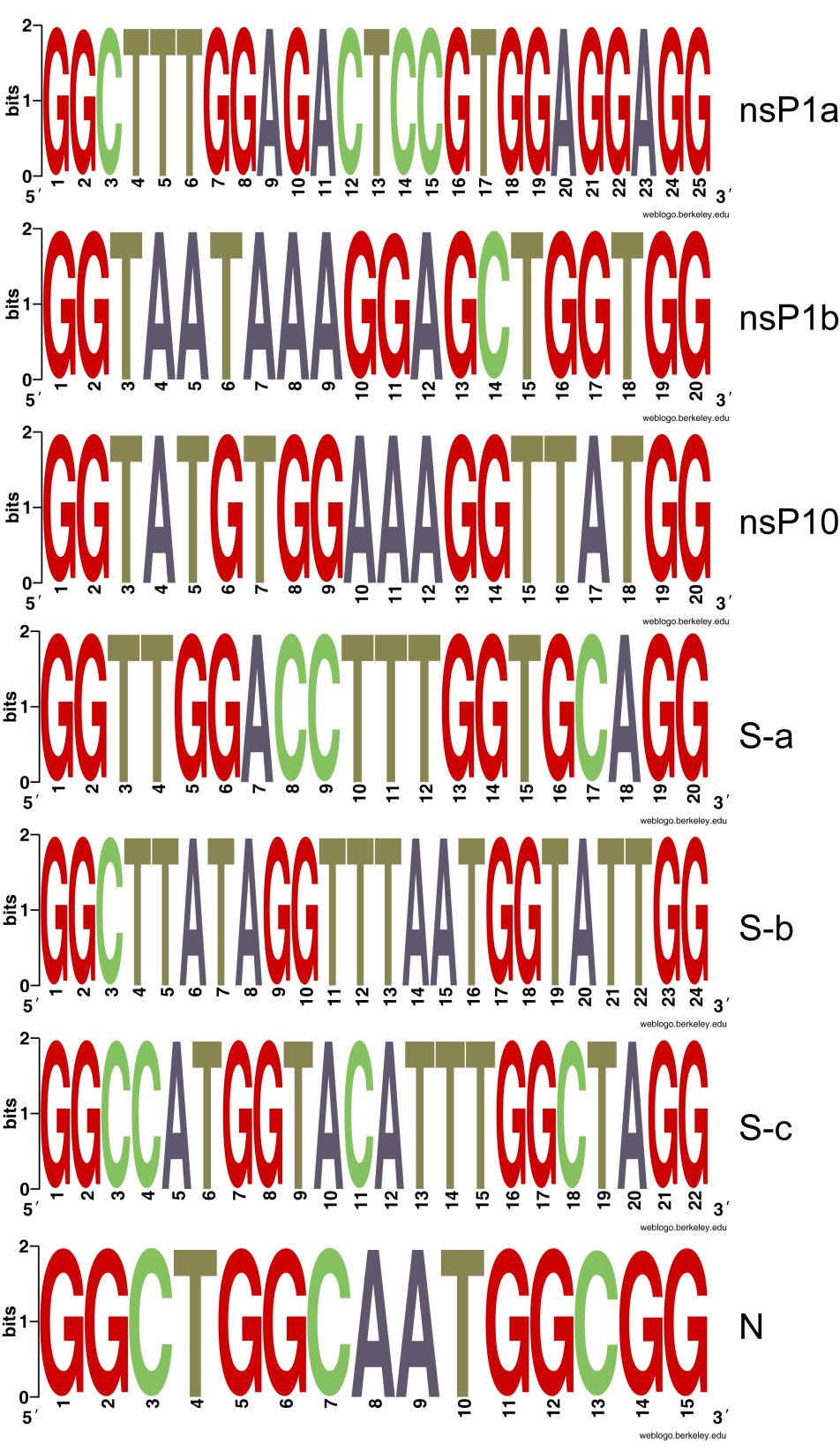


**Figure S2** Conserved G-quadruplex sequence logos formed by SARSr-CoV.


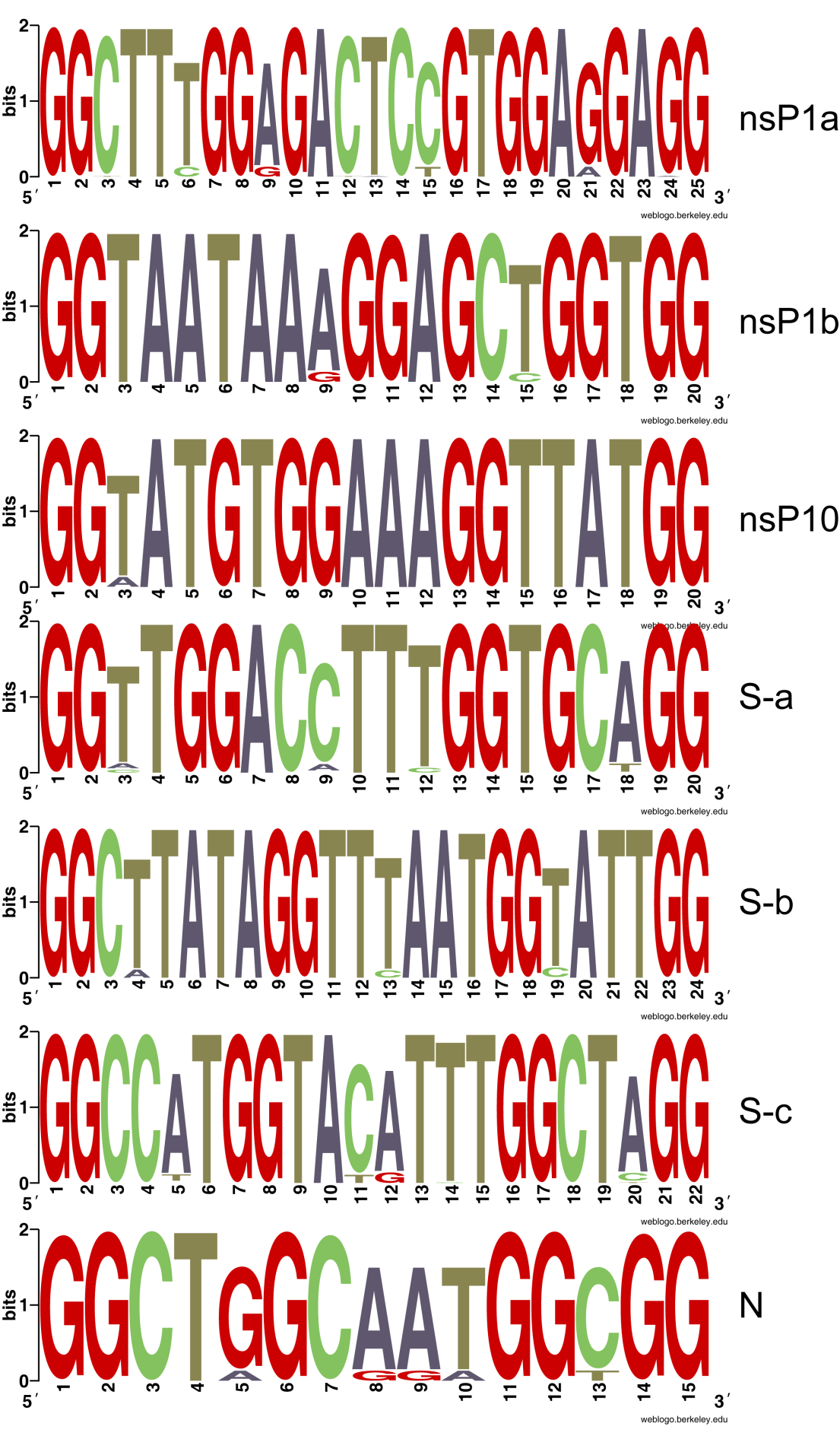


**Figure S3** Amino acid alignment of nsP3 SARS unique domain in human coronaviruses.


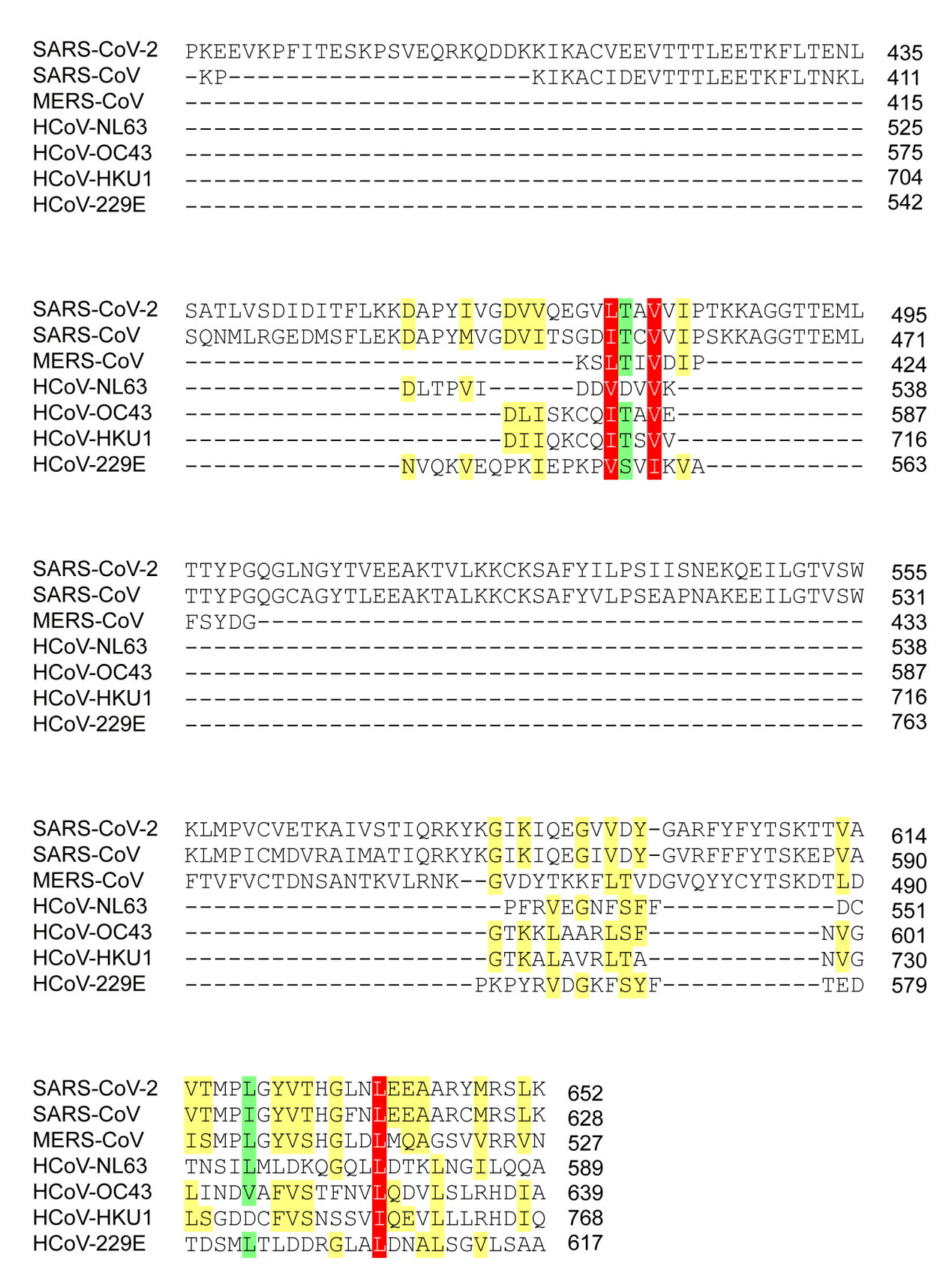


**Table S1** Predicted G-quadruplex sequences in seven human coronaviruses.

| SARS-CoV-2 | | | |
| --- | --- | --- | --- |
| Position | Length | QGRS | G-Score |
| 353 | 25 | GGCTTTGGAGACTCCGTGGAGGAGG | 16 |
| 644 | 20 | GGTAATAAAGGAGCTGGTGG | 15 |
| 1574 | 26 | GGTGTTGTTGGAGAAGGTTCCGAAGG | 18 |
| 2714 | 29 | GGCGGTGCACCAACAAAGGTTACTTTTGG | 10 |
| 3467 | 17 | GGAGGAGGTGTTGCAGG | 15 |
| 4262 | 28 | GGTTTAAATGGTTACACTGTAGAGGAGG | 10 |
| 8687 | 23 | GGATACAAGGCTATTGATGGTGG | 14 |
| 13385 | 20 | GGTATGTGGAAAGGTTATGG | 19 |
| 18296 | 23 | GGATTGGCTTCGATGTCGAGGGG | 9 |
| 24215 | 20 | GGTTGGACCTTTGGTGCAGG | 17 |
| 24268 | 24 | GGCTTATAGGTTTAATGGTATTGG | 19 |
| 25197 | 22 | GGCCATGGTACATTTGGCTAGG | 17 |
| 28903 | 15 | GGCTGGCAATGGCGG | 18 |
| 29123 | 19 | GGAAATTTTGGGGACCAGG | 14 |

| SARS-CoV | | | | |
| --- | --- | --- | --- | --- |
| Position | Length | QGRS | | G-Score |
| 352 | 25 | GGCTTCGGGGACTCTGTGGAAGAGG | | 18 |
| 516 | 30 | GGTCGTTGAGCTGGTTGCAGAAATGGACGG | | 13 |
| 643 | 20 | GGTAATAAGGGAGCCGGTGG | | 16 |
| 1461 | 24 | GGGAGGTAGGACTAGATGTTTTGG | | 11 |
| 1558 | 26 | GGCTCAGGCCATACTGGCATTACTGG | | 18 |
| 3186 | 26 | GGAAGAAGAAGAGGAAGACTGGCTGG | | 13 |
| 3400 | 17 | GGTGGTGGTGTAGCAGG | | 15 |
| 7159 | 25 | GGTCTGGCCGCTGAGTGGGTTTTGG | | 15 |
| 9988 | 29 | GGTTTTAGGAAAATGGCATTCCCGTCAGG | | 15 |
| 12078 | 30 | GGAGGCCTATGAGCAGGCTGTAGCTAATGG | | 11 |
| 12723 | 20 | GGGAGGTAGGTTTGTGCTGG | | 15 |
| 13315 | 20 | GGAATGTGGAAAGGTTATGG | | 19 |
| 13541 | 18 | GGAGAAGGATGAGGAAGG | | 19 |
| 14000 | 30 | GGATCTTAATGGGAACTGGTACGATTTCGG | | 17 |
| 19923 | 19 | GGTAGAGTGGAAGGACAGG | | 17 |
| 21210 | 28 | GGGGCTAACTATCTTGGCAAGCCGAAGG | | 10 |
| 24090 | 20 | GGATGGACATTTGGTGCTGG | | 17 |
| 24143 | 24 | GGCATATAGGTTCAATGGCATTGG | | 19 |
| 24404 | 30 | GGAGGTACAAATTGACAGGTTAATTACAGG | | 10 |
| 25072 | 22 | GGCCTTGGTATGTTTGGCTCGG | | 17 |
| 25829 | 26 | GGTTATTCTGAGGATAGGCACTCAGG | | 15 |
| 28210 | 21 | GGAGGACGCAATGGGGCAAGG | | 15 |
| 28630 | 29 | GGCTTCTACGCAGAGGGAAGCAGAGGCGG | | 10 |
| 28752 | 15 | GGCTAGCGGAGGTGG | | 17 |
| MERS-CoV | | | | |
| Position | Length | QGRS | G-Score | |
| 244 | 27 | GGTTTCGTCCGGTGCGTGGCAATTCGG | 18 | |
| 618 | 28 | GGCAGCCTGGTTGGCACAACTTTGCAGG | 11 | |
| 791 | 27 | GGACGATTTTGAGGCGGATCCTAAAGG | 12 | |
| 929 | 26 | GGCCAAGGATGGAATAACCAAACTGG | 11 | |
| 1339 | 21 | GGTTTGCCTGTGGATGTGGGG | 12 | |
| 1659 | 25 | GGCTGTACTGGCTCTTGGAACAAGG | 19 | |
| 2347 | 19 | GGTTTGTGGTGGTCAATGG | 17 | |
| 3735 | 17 | GGCGGTGGTATCGCTGG | 15 | |
| 4982 | 26 | GGCTGCAGTCCATGGGTGGAAGATGG | 12 | |
| 5276 | 30 | GGTTTGGAGAGAGTGGTGCAATGTCTGTGG | 12 | |
| 7187 | 27 | GGCCTTCAACTGGTTGTTGTTGGCAGG | 14 | |
| 10061 | 27 | GGCTTGTATGGTTCAGGTTACCTGCGG | 17 | |
| 10464 | 26 | GGTAGTGTTGGTTACACCAAGGAGGG | 14 | |
| 12815 | 24 | GGCGCGTGTTGAAGGTAAGGACGG | 12 | |
| 13531 | 21 | GGCTAAGGTTGCTGGTATTGG | 20 | |
| 13999 | 21 | GGTCAAGGCTGGTTTAGTCGG | 16 | |
| 14041 | 30 | GGACCTTAATGGCAAGTGGTATGATTTTGG | 17 | |
| 14680 | 27 | GGTATCTAAAGGTTTCTTTAAGGAGGG | 14 | |
| 20559 | 18 | GGTGTAAGGATGGACAGG | 18 | |
| 22569 | 26 | GGCTCAGTTGTGGAACAGGCTGAAGG | 16 | |
| 23103 | 12 | GGTGGTGGCTGG | 20 | |
| 24324 | 20 | GGTGTTGGCTGGACTGCTGG | 17 | |
| 24391 | 30 | GGTTAAACGGTGTTGGCATTACTCAACAGG | 13 | |
| 25339 | 22 | GGCCGTGGTACATTTGGCTTGG | 17 | |
| 26091 | 21 | GGATGGAGGAATCCCTGATGG | 12 | |
| 28791 | 19 | GGGTATTGGCGGAGACAGG | 17 | |
| 28827 | 30 | GGGAATGGAATTAAGCAACTGGCTCCCAGG | 13 | |
| 28906 | 26 | GGGCTGTTAAGGATGGCATCGTTTGG | 15 | |
| 29054 | 12 | GGGGACTGGAGG | 18 | |
| 29169 | 26 | GGTCCATCTGGAATCGGAGCAGTAGG | 18 | |
| 29364 | 28 | GGTCTTCGCGGACCAGGAGACCTCCAGG | 16 | |

| HCoV-NL63 | | | |  |
| --- | --- | --- | --- | --- |
| Position | Length | QGRS | G-Score | |
| 602 | 22 | GGCCATGGTGCAGGAAGTGTGG | 19 | |
| 1041 | 22 | GGAGTGTTGGTGCATGGGATGG | 18 | |
| 4253 | 23 | GGTCCACTTAAGGTTGGAGCAGG | 14 | |
| 8103 | 15 | GGTTGGAAGGTTTGG | 20 | |
| 9746 | 15 | GGTTGTAGGTGGTGG | 17 | |
| 9788 | 29 | GGTTTTAATGAATGGGCTATGGCTAATGG | 15 | |
| 10653 | 26 | GGGGCATTTTGTATTGGTTCAATAGG | 10 | |
| 11735 | 26 | GGTGATGGTGGTGTTTTAGGTGATGG | 18 | |
| 12368 | 21 | GGTTGTTGGTTGGGACACGGG | 19 | |
| 14351 | 27 | GGTTTTATAGGCTTGGTAATGAGTTGG | 15 | |
| 17239 | 16 | GGTTGGTTGGGTATGG | 20 | |
| 19786 | 26 | GGTTCTGACAAAGGTGTGGCACCTGG | 14 | |
| 21192 | 22 | GGTTCCACACTAGTGGACGGGG | 9 | |
| 23274 | 17 | GGTGGCATGGTGCTCGG | 17 | |
| 24355 | 17 | GGCCTTGGTGGGTTTGG | 19 | |
| 26421 | 26 | GGTGTTGTTTGGGTTGCTAAGGAAGG | 15 | |

| HCoV-OC43 | | | |  |
| --- | --- | --- | --- | --- |
| Position | Length | QGRS | G-Score | |
| 265 | 30 | GGATGTTTGAGGACGCAGAGGAGAAGTTGG | 20 | |
| 582 | 24 | GGATGGACCATGGGTTTGTTTCGG | 15 | |
| 696 | 25 | GGTGCAGGTCAATTCGTGGGTTGGG | 16 | |
| 825 | 17 | GGACGTGGCGGTTTTGG | 18 | |
| 1545 | 17 | GGCTATGGTGGTTTTGG | 18 | |
| 1688 | 29 | GGTTTATACTGGAGTTTTAGGTTGCAAGG | 19 | |
| 3236 | 29 | GGACACTTTGGATGATGGTCCAAGTGTGG | 17 | |
| 4113 | 23 | GGCCATATGGCACATGGTGGTGG | 19 | |
| 5339 | 19 | GGTTTTGGCCAAAGGTGGG | 18 | |
| 6070 | 30 | GGCCAACAGCTACAGGTGATGTGGTGTTGG | 13 | |
| 7261 | 21 | GGAGTTTTAGGTTGCTGGTGG | 15 | |
| 7484 | 18 | GGCTAATGGTGGCACTGG | 17 | |
| 10032 | 22 | GGTTTATGGTTGGATGACAAGG | 16 | |
| 10482 | 25 | GGGGATTTTTATGGTCCTTATAAGG | 12 | |
| 13263 | 17 | GGATTTTGGCGGGATGG | 18 | |
| 13894 | 30 | GGACAAATTGGTGGAGGTAGGCTTAGTAGG | 20 | |
| 16348 | 24 | GGTAATGAATGGTCTGGTTTTTGG | 16 | |
| 17101 | 17 | GGCCAAGGTCAGGGTGG | 19 | |
| 19255 | 23 | GGATGGCATGGATGCTAAGCAGG | 13 | |
| 19508 | 23 | GGTCATTATACAGGACAGGCTGG | 13 | |
| 19951 | 15 | GGTGGACAAGGTTGG | 18 | |
| 20069 | 29 | GGTAATCTGGGGAGTAATGGTAAACCCGG | 21 | |
| 25131 | 26 | GGTTCGTGTGTAGGTAGTGGTCCTGG | 15 | |
| 29602 | 30 | GGTTTCCGCCTGGCACGGTACTCCCTCAGG | 14 | |

| HCoV-HKU1 | | | |
| --- | --- | --- | --- |
| Position | Length | QGRS | G-Score |
| 261 | 30 | GGCTGCTTCCGGATGCAGCGGAGGAGTTGG | 20 |
| 541 | 30 | GGGTGGTTTTCCTAAAGGGTATGTTATGGG | 13 |
| 1325 | 25 | GGTTGGGTTTCAGGTAATATGATGG | 15 |
| 4371 | 22 | GGCATATGCTCCATGGTGGTGG | 10 |
| 5532 | 20 | GGCAGTGGCAGGAAGCATGG | 17 |
| 7188 | 24 | GGATAAAGGCTACTTTTGGTTTGG | 16 |
| 18418 | 25 | GGTTGGGTTGGTTTTGATGTTGAGG | 11 |
| 20329 | 25 | GGTAATCTAGGTGGTAATTGCGCGG | 13 |

| HCoV-229E | | | |
| --- | --- | --- | --- |
| Position | Length | QGRS | G-Score |
| 608 | 17 | GGTAAGAGAGGTGGTGG | 15 |
| 1050 | 22 | GGTCTGTTGGTGATTGGACCGG | 18 |
| 2450 | 20 | GGAGGCAAAATATGGAATGG | 14 |
| 2867 | 19 | GGCTGTGGTGGTATTTTGG | 16 |
| 4564 | 30 | GGTTTGTAAGGTTAAGGATTTTGTGTCTGG | 14 |
| 8462 | 27 | GGTTACGTTTGTGGTACTGGTTTGTGG | 15 |
| 9827 | 23 | GGTTGCACATGGTGGCTTAAAGG | 14 |
| 9998 | 25 | GGCTTTGGTGGTAAACAAATTTTGG | 10 |
| 11810 | 17 | GGTGAAGGTGATGGAGG | 18 |
| 12045 | 28 | GGAGAGGTGCTGTTTTGGGTTACATTGG | 15 |
| 12233 | 19 | GGTTCTGGTAGCGGTCAGG | 20 |
| 17324 | 18 | GGTTAGGAATGGATGTGG | 20 |
| 17610 | 24 | GGTGTTTGTACTTTGGGCTGGCGG | 10 |
| 19405 | 28 | GGCATCTTAAAGGCAGAGGAGTTTGTGG | 16 |
| 20062 | 23 | GGTAGGACAAAGGCAATTGATGG | 15 |
| 20864 | 26 | GGTACTGGGAGAGGTGATTGTAAAGG | 15 |
| 21825 | 25 | GGAGTGATGGTGATGGAATTACTGG | 18 |
| 22817 | 29 | GGTTCTTTAATTGGTGGAATTGCTTTAGG | 12 |
| 23910 | 17 | GGCCGTGGTGGGTGTGG | 19 |
| 25448 | 28 | GGACATAGATTGGCTTCAGGTGTTCAGG | 17 |
| 26508 | 26 | GGAAGTGCAGGTGTTGTGGCCAATGG | 19 |

Table S2 Predicted G-quadruplex sequences in negative strands of seven human coronaviruses.

| SARS-CoV-2 | | | |
| --- | --- | --- | --- |
| Position | Length | QGRS | G-Score |
| 165 | 22 | GGCCTCGGTGAAAATGTGGTGG | 13 |
| 13136 | 19 | GGTTAAGTGGTGGTCTAGG | 16 |
| 13963 | 18 | GGATCTGGGTAAGGAAGG | 19 |
| 16623 | 27 | GGATTTGGATGATCTATGTGGCAACGG | 14 |
| 19865 | 25 | GGTGATAGAGGTTTGTGGTGGTTGG | 19 |
| 23877 | 17 | GGATATGGTTGGTTTGG | 19 |
| 25003 | 16 | GGTGGAATGTGGTAGG | 17 |
| 27432 | 24 | GGGGCTTTTAGAGGCATGAGTAGG | 13 |
| 29867 | 23 | GGTTGGTTTGTTACCTGGGAAGG | 13 |

| SARS-CoV | | | |
| --- | --- | --- | --- |
| Position | Length | QGRS | G-Score |
| 1178 | 29 | GGATTGCGGGTGCCAATGTGGTCTTTGGG | 18 |
| 1402 | 30 | GGTGTTGATTGGAACGCCCTGGCCTCGAGG | 19 |
| 1493 | 29 | GGTAAACCTTGGGGTCGGCGCTGTTTTGG | 16 |
| 1590 | 27 | GGGGGGCACTACGTTGGTTTGATTGGG | 14 |
| 5158 | 17 | GGAAGGACATAAGGTGG | 16 |
| 6713 | 30 | GGATAATTTTGGTCCACAAACCGTGGCCGG | 11 |
| 6857 | 30 | GGTGCAAGGTTTGCCATCAGGGGAGAAAGG | 16 |
| 10999 | 29 | GGCAATGTTGGTCATGGTTACTCTGAAGG | 15 |
| 11350 | 16 | GGTCACCTGGTGGAGG | 16 |
| 11629 | 28 | GGTAGGTCATGTCCTTTGGTATGCCTGG | 12 |
| 12361 | 22 | GGGGGGCTGGTAATTGAGCAGG | 13 |
| 13881 | 18 | GGATCTGGGTAAGGCAGG | 19 |
| 16541 | 21 | GGATTTGGATGGTCAATGTGG | 16 |
| 17668 | 26 | GGCCTCCTGGGCAGTGGCATAAGCGG | 19 |
| 23801 | 26 | GGTTGAGTTGGTACAAGGTCTATAGG | 19 |
| 26492 | 20 | GGTGTAGGTTCTGGTTCTGG | 21 |
| 28362 | 22 | GGTCTTGACAGGCAGGACATGG | 15 |
| 29713 | 25 | GGTTGGTTGGCTTTTCCTGGGTAGG | 16 |

| MERS-CoV | | | |
| --- | --- | --- | --- |
| Position | Length | QGRS | G-Score |
| 244 | 23 | GGACAAGCGATGGTGAGATGGGG | 12 |
| 338 | 25 | GGACCAGGCTGAACACTTGGACGGG | 14 |
| 1343 | 23 | GGGGTAGAATTGGCATTAAGAGG | 13 |
| 1379 | 17 | GGTGGAAAGGTAAGAGG | 17 |
| 1516 | 30 | GGCAAAGGAAACAGCACGAGGTGCAGCAGG | 14 |
| 1752 | 27 | GGGTTTGGCCACGGTGACTTCATTAGG | 14 |
| 2298 | 14 | GGTGGTAGAGGGGG | 18 |
| 3228 | 26 | GGAGAAACTGGGACTAGCTGGACGGG | 16 |
| 3489 | 21 | GGGAGGCCACTATGGTAAAGG | 16 |
| 3992 | 25 | GGAGGTAGAGGGAACATCCATCAGG | 11 |
| 5003 | 12 | GGAGGAGGGAGG | 20 |
| 5067 | 19 | GGGAGGTAATGGGCTCAGG | 18 |
| 6151 | 29 | GGCACCATGGAGAGCCTGGTTTATTTTGG | 19 |
| 6522 | 29 | GGACACAACCAACAGGTGTCTGAAGGGGG | 10 |
| 7069 | 23 | GGATGGGACAATGGATACACAGG | 17 |
| 14208 | 18 | GGGTCTGGATAAGGAAGG | 19 |
| 15560 | 26 | GGCAATGTGCATGGCTGGATCAGCGG | 13 |
| 16811 | 30 | GGGTCACGGACACACTGAGCAGGGATTTGG | 14 |
| 18688 | 17 | GGCAATGGGGCTTGAGG | 16 |
| 21086 | 27 | GGATCATGGCAGTATGGTGTCATACGG | 18 |
| 22496 | 24 | GGCCTGCGTAGGGCGGTAGTGAGG | 15 |
| 26614 | 27 | GGGCTGGATAGTCTGAGGTGCAGACGG | 15 |
| 28843 | 22 | GGAGTGGTAAATGTAGGTTGGG | 16 |
| 29574 | 23 | GGTACACACGAGGACCTGGGAGG | 14 |

| HCoV-NL63 | | | |
| --- | --- | --- | --- |
| Position | Length | QGRS | G-Score |
| 766 | 25 | GGCTTATTAGGTTTCTTAGGAGTGG | 17 |
| 5036 | 19 | GGTCTGGTTGGTTACATGG | 17 |
| 6040 | 23 | GGTTGAGAGGACGGGCCAAAAGG | 18 |
| 9799 | 18 | GGTGGTTTTGGCTCAAGG | 17 |
| 12654 | 25 | GGATCTGGGTAAGGCAAATAATAGG | 16 |
| 27178 | 26 | GGTGCGAACGGCTACAGAAGGAATGG | 16 |

| HCoV-OC43 | | | |
| --- | --- | --- | --- |
| Position | Length | QGRS | G-Score |
| 838 | 19 | GGCTCCTCTTCTGGCGGGG | 11 |
| 975 | 22 | GGTGTTACACCAGAGGTAGGGG | 9 |
| 1570 | 26 | GGTCGGACTGATCGGCCCACTTGAGG | 14 |
| 6996 | 13 | GGAGGAGGACCGG | 19 |
| 7274 | 26 | GGACATCTGCCATAGGGGTAGAGAGG | 9 |
| 8743 | 19 | GGTGATGAGGGGTTGATGG | 15 |
| 14938 | 18 | GGATTAGGATATGGAAGG | 19 |
| 20816 | 19 | GGAAGCAGTAGGCGGTTGG | 14 |
| 29990 | 27 | GGATTGCACAGGCATAAAGGCTAAGGG | 18 |

| HCoV-HKU1 | | | |
| --- | --- | --- | --- |
| Position | Length | QGRS | G-Score |
| 1200 | 23 | GGATGCATTGGCATATGGGCCGG | 17 |
| 5499 | 20 | GGACAGGAAGCAGAAGGTGG | 14 |
| 7433 | 25 | GGTAAGGATAAAAAAGGTGCAGTGG | 16 |

| HCoV-229E | | | |
| --- | --- | --- | --- |
| postion | Length | QGRS | G-Score |
| 819 | 17 | GGTCAAGGTCTCTGGGG | 16 |
| 1185 | 22 | GGGAAGGAGCACGGGAGTCAGG | 18 |
| 4972 | 25 | GGACCGAAGTGGTCCAATTGGAAGG | 15 |
| 10182 | 27 | GGCATGACTTGGTGGTAAATCAATAGG | 12 |
| 11510 | 18 | GGTTTAACGGTGGTTTGG | 16 |
| 12332 | 19 | GGCTAGGATCTGGATATGG | 20 |
| 13080 | 26 | GGTGGCATTTCTGGTAGCTACTATGG | 12 |
| 22670 | 27 | GGACACTGGTTTTGGTTCTATTTTAGG | 15 |
| 26316 | 28 | GGTTTAGTAAAGGCCTCTAGGATGTTGG | 17 |
| 26924 | 23 | GGCCTCGCTATAACGGCGGACGG | 10 |
| 27013 | 29 | GGTTGCAGGCCATTAGGAACAGTTACTGG | 16 |

Table S3 Conservation analysis on G-quadruplex sequences in human coronaviruses.

| Human coronavirus | nsP1-a | nsP1-b | nsP10 | S-a | S-b | S-c | N |
| --- | --- | --- | --- | --- | --- | --- | --- |
| SARS-CoV-2 | GGCTTTGGAGACTCCGTGGAGGAGG | GGTAATAAAGGAGCTGGTGG | GGTATGTGGAAAGGTTATGG | GGTTGGACCTTTGGTGCAGG | GGCTTATAGGTTTAATGGTATTGG | GGCCATGGTACATTTGGCTAGG | GGCTGGCAATGGCGG |
| SARS-CoV | GGCTTCGGGGACTCTGTGGAAGAGG | GGTAATAAGGGAGCCGGTGG | GGAATGTGGAAAGGTTATGG | GGATGGACATTTGGTGCTGG | GGCATATAGGTTCAATGGCATTGG | GGCCTTGGTATGTTTGGCTCGG | GGCTAGCGGAGGTGG |
| MERS-CoV |  |  |  |  |  | GGCCGTGGTACATTTGGCTTGG |  |
| HCoV-NL63 |  |  | GGTTGTTGGTTGGGACACGGG |  |  | GGCCTTGGTGGGTTTGG |  |
| HCoV-OC43 |  |  | GGATTTTGGCGGGATGG |  |  |  |  |
| HCoV-HKU1 |  |  |  |  |  |  |  |
| HCoV-229E |  |  |  |  |  | GGCCGTGGTGGGTGTGG |  |

**Table S4** Results comparison of two prediction website in human coronaviruses. This table showed G-quadruplex sequences predicted by Quadbase2 tool. The lines in yellow indicated the consistent results of conserved sequences with QGRS mapper.

| name | postion | length | G-quadruplexes sequence |
| --- | --- | --- | --- |
| SARS-CoV-2 | 235 | 34 | GGTTTCGTCCGGGTGTGACCGAAAGGTAAGATGG |
| SARS-CoV-2 | 352 | 25 | GGCTTTGGAGACTCCGTGGAGGAGG |
| SARS-CoV-2 | 544 | 35 | GGCATTCAGTACGGTCGTAGTGGTGAGACACTTGG |
| SARS-CoV-2 | 643 | 32 | GGTAATAAAGGAGCTGGTGGCCATAGTTACGG |
| SARS-CoV-2 | 1573 | 26 | GGTGTTGTTGGAGAAGGTTCCGAAGG |
| SARS-CoV-2 | 2713 | 29 | GGCGGTGCACCAACAAAGGTTACTTTTGG |
| SARS-CoV-2 | 3466 | 31 | GGAGGAGGTGTTGCAGGAGCCTTAAATAAGG |
| SARS-CoV-2 | 4254 | 32 | GGGTCAGGGTTTAAATGGTTACACTGTAGAGG |
| SARS-CoV-2 | 5035 | 32 | GGACAACAGTTTGGTCCAACTTATTTGGATGG |
| SARS-CoV-2 | 8686 | 23 | GGATACAAGGCTATTGATGGTGG |
| SARS-CoV-2 | 10057 | 41 | GGTTTTAGAAAAATGGCATTCCCATCTGGTAAAGTTGAGGG |
| SARS-CoV-2 | 13384 | 20 | GGTATGTGGAAAGGTTATGG |
| SARS-CoV-2 | 20868 | 32 | GGTGCTGGTTCTGATAAAGGAGTTGCACCAGG |
| SARS-CoV-2 | 24214 | 20 | GGTTGGACCTTTGGTGCAGG |
| SARS-CoV-2 | 24267 | 24 | GGCTTATAGGTTTAATGGTATTGG |
| SARS-CoV-2 | 25196 | 34 | GGCCATGGTACATTTGGCTAGGTTTTATAGCTGG |
| SARS-CoV-2 | 28612 | 32 | GGAACTGGGCCAGAAGCTGGACTTCCCTATGG |
| SARS-CoV-2 | 28902 | 15 | GGCTGGCAATGGCGG |
| SARS-CoV-2 | 29253 | 38 | GGGAACGTGGTTGACCTACACAGGTGCCATCAAATTGG |

| name | postion | length | G-quadruplex sequence |
| --- | --- | --- | --- |
| SARS-CoV | 234 | 34 | GGTTTCGTCCGGGTGTGACCGAAAGGTAAGATGG |
| SARS-CoV | 351 | 25 | GGCTTCGGGGACTCTGTGGAAGAGG |
| SARS-CoV | 408 | 32 | GGCACTTGTGGTCTAGTAGAGCTGGAAAAAGG |
| SARS-CoV | 507 | 34 | GGCCACAAGGTCGTTGAGCTGGTTGCAGAAATGG |
| SARS-CoV | 543 | 35 | GGCATTCAGTACGGTCGTAGCGGTATAACACTGGG |
| SARS-CoV | 642 | 32 | GGTAATAAGGGAGCCGGTGGTCATAGCTATGG |
| SARS-CoV | 1460 | 27 | GGGAGGTAGGACTAGATGTTTTGGAGG |
| SARS-CoV | 1557 | 26 | GGCTCAGGCCATACTGGCATTACTGG |
| SARS-CoV | 3117 | 34 | GGTACAGAGGATGATTATCAAGGTCTCCCTCTGG |
| SARS-CoV | 3185 | 26 | GGAAGAAGAAGAGGAAGACTGGCTGG |
| SARS-CoV | 3399 | 31 | GGTGGTGGTGTAGCAGGTGCACTCAACAAGG |
| SARS-CoV | 4182 | 31 | GGACAAGGATGTGCTGGTTATACACTTGAGG |
| SARS-CoV | 4965 | 32 | GGACAGCAGTTTGGTCCAACATACTTGGATGG |
| SARS-CoV | 6465 | 31 | GGTCATGAGGATCTTATGGCTGCTTATGTGG |
| SARS-CoV | 7158 | 25 | GGTCTGGCCGCTGAGTGGGTTTTGG |
| SARS-CoV | 8691 | 32 | GGTTTTGACGCATGGTTTAGCCAGCGTGGTGG |
| SARS-CoV | 9987 | 42 | GGTTTTAGGAAAATGGCATTCCCGTCAGGCAAAGTTGAAGGG |
| SARS-CoV | 12077 | 30 | GGAGGCCTATGAGCAGGCTGTAGCTAATGG |
| SARS-CoV | 12722 | 20 | GGGAGGTAGGTTTGTGCTGG |
| SARS-CoV | 13314 | 20 | GGAATGTGGAAAGGTTATGG |
| SARS-CoV | 13540 | 18 | GGAGAAGGATGAGGAAGG |
| SARS-CoV | 13999 | 30 | GGATCTTAATGGGAACTGGTACGATTTCGG |
| SARS-CoV | 19922 | 19 | GGTAGAGTGGAAGGACAGG |
| SARS-CoV | 20798 | 32 | GGTGCTGGCTCTGATAAAGGAGTTGCACCAGG |
| SARS-CoV | 21210 | 40 | GGGCTAACTATCTTGGCAAGCCGAAGGAACAAATTGATGG |
| SARS-CoV | 24089 | 20 | GGATGGACATTTGGTGCTGG |
| SARS-CoV | 24142 | 24 | GGCATATAGGTTCAATGGCATTGG |
| SARS-CoV | 24400 | 33 | GGCGGAGGTACAAATTGACAGGTTAATTACAGG |
| SARS-CoV | 25071 | 34 | GGCCTTGGTATGTTTGGCTCGGCTTCATTGCTGG |
| SARS-CoV | 25825 | 29 | GGTGGTTATTCTGAGGATAGGCACTCAGG |
| SARS-CoV | 26832 | 33 | GGTCACTTGCGAATGGCCGGACACTCCCTAGGG |
| SARS-CoV | 28209 | 21 | GGAGGACGCAATGGGGCAAGG |
| SARS-CoV | 28629 | 29 | GGCTTCTACGCAGAGGGAAGCAGAGGCGG |
| SARS-CoV | 28751 | 15 | GGCTAGCGGAGGTGG |
| SARS-CoV | 29102 | 38 | GGGAACATGGCTGACTTATCATGGAGCCATTAAATTGG |

| name | postion | length | G-quadruplex sequence |
| --- | --- | --- | --- |
| MERS-CoV | 230 | 32 | GGTCACAATACACGGTTTCGTCCGGTGCGTGG |
| MERS-CoV | 617 | 41 | GGCAGCCTGGTTGGCACAACTTTGCAGGGCAAGCCTATTGG |
| MERS-CoV | 786 | 31 | GGATGGACGATTTTGAGGCGGATCCTAAAGG |
| MERS-CoV | 928 | 26 | GGCCAAGGATGGAATAACCAAACTGG |
| MERS-CoV | 1292 | 33 | GGAAGTTGTGGTAATGATTCCTGGCTTACAGGG |
| MERS-CoV | 1658 | 25 | GGCTGTACTGGCTCTTGGAACAAGG |
| MERS-CoV | 2345 | 20 | GGGTTTGTGGTGGTCAATGG |
| MERS-CoV | 3734 | 31 | GGCGGTGGTATCGCTGGTGCTATTAATGCGG |
| MERS-CoV | 4526 | 32 | GGTGGTTACAATTCTTGGCATTTAGTCGAGGG |
| MERS-CoV | 4981 | 26 | GGCTGCAGTCCATGGGTGGAAGATGG |
| MERS-CoV | 5275 | 30 | GGTTTGGAGAGAGTGGTGCAATGTCTGTGG |
| MERS-CoV | 6570 | 34 | GGCATCTAGGTGTTACTAAAGGCATATTGACAGG |
| MERS-CoV | 7186 | 27 | GGCCTTCAACTGGTTGTTGTTGGCAGG |
| MERS-CoV | 10049 | 28 | GGAGATGTTGAGGCTTGTATGGTTCAGG |
| MERS-CoV | 10454 | 34 | GGTTCTTGTGGTAGTGTTGGTTACACCAAGGAGG |
| MERS-CoV | 12813 | 25 | GGGCGCGTGTTGAAGGTAAGGACGG |
| MERS-CoV | 13530 | 21 | GGCTAAGGTTGCTGGTATTGG |
| MERS-CoV | 13998 | 21 | GGTCAAGGCTGGTTTAGTCGG |
| MERS-CoV | 14040 | 30 | GGACCTTAATGGCAAGTGGTATGATTTTGG |
| MERS-CoV | 14679 | 27 | GGTATCTAAAGGTTTCTTTAAGGAGGG |
| MERS-CoV | 15964 | 34 | GGTACACTCATGGTAGAGCGGTTTGTGTCTTTGG |
| MERS-CoV | 16529 | 31 | GGTTGGCTACCTGTGACTGGACTGAAAGTGG |
| MERS-CoV | 20558 | 18 | GGTGTAAGGATGGACAGG |
| MERS-CoV | 20812 | 32 | GGCGCTGGTTCTGATAAAGGTATCGCTCCTGG |
| MERS-CoV | 22568 | 26 | GGCTCAGTTGTGGAACAGGCTGAAGG |
| MERS-CoV | 23102 | 26 | GGTGGTGGCTGGCTTGTTGCTAGTGG |
| MERS-CoV | 24311 | 32 | GGCAGCATAGCAGGTGTTGGCTGGACTGCTGG |
| MERS-CoV | 24390 | 30 | GGTTAAACGGTGTTGGCATTACTCAACAGG |
| MERS-CoV | 25338 | 35 | GGCCGTGGTACATTTGGCTTGGTTTCATTGCTGGG |
| MERS-CoV | 26077 | 34 | GGTTGCTACATAAGGATGGAGGAATCCCTGATGG |
| MERS-CoV | 28287 | 32 | GGCCACCTCAAAATGGCTGGCATGCATTTCGG |
| MERS-CoV | 28391 | 33 | GGTGAAGCGGCAAAGCTACGGAACTAATTCCGG |
| MERS-CoV | 28790 | 19 | GGGTATTGGCGGAGACAGG |
| MERS-CoV | 28826 | 33 | GGGAATGGAATTAAGCAACTGGCTCCCAGGTGG |
| MERS-CoV | 28905 | 40 | GGGCTGTTAAGGATGGCATCGTTTGGGTCCATGAAGATGG |
| MERS-CoV | 29156 | 29 | GGCACTTCTCCAGGTCCATCTGGAATCGG |
| MERS-CoV | 29350 | 30 | GGTGCAAGCTTTTGGTCTTCGCGGACCAGG |

| name | postion | length | G-quadruplex sequence |
| --- | --- | --- | --- |
| HCoV-NL63 | 601 | 31 | GGCCATGGTGCAGGAAGTGTGGTTTTTGTGG |
| HCoV-NL63 | 1027 | 35 | GGTTCTGAGAGTTGGAGTGTTGGTGCATGGGATGG |
| HCoV-NL63 | 4252 | 34 | GGTCCACTTAAGGTTGGAGCAGGTGTTATGTTGG |
| HCoV-NL63 | 8102 | 19 | GGTTGGAAGGTTTGGGTGG |
| HCoV-NL63 | 8323 | 41 | GGTGTTTGTTTTGGTTTTGATAAATGGTATGTTAATGATGG |
| HCoV-NL63 | 9745 | 15 | GGTTGTAGGTGGTGG |
| HCoV-NL63 | 9787 | 29 | GGTTTTAATGAATGGGCTATGGCTAATGG |
| HCoV-NL63 | 11733 | 27 | GGGTGATGGTGGTGTTTTAGGTGATGG |
| HCoV-NL63 | 12289 | 34 | GGTAAATGTGTTCAGGTTCCTATTGGTTGTTTGG |
| HCoV-NL63 | 12367 | 21 | GGTTGTTGGTTGGGACACGGG |
| HCoV-NL63 | 14350 | 27 | GGTTTTATAGGCTTGGTAATGAGTTGG |
| HCoV-NL63 | 17238 | 16 | GGTTGGTTGGGTATGG |
| HCoV-NL63 | 19779 | 32 | GGTGCTGGTTCTGACAAAGGTGTGGCACCTGG |
| HCoV-NL63 | 23248 | 35 | GGCCATGTACACAGGTTCTCTTATAGGTGGCATGG |
| HCoV-NL63 | 24354 | 17 | GGCCTTGGTGGGTTTGG |
| HCoV-NL63 | 26050 | 32 | GGTGATTTTTCTGGTGTTGCCTCTCAGGAGGG |
| HCoV-NL63 | 26420 | 26 | GGTGTTGTTTGGGTTGCTAAGGAAGG |

| name | postion | length | G-quadruplex sequence |
| --- | --- | --- | --- |
| HCoV-OC43 | 264 | 30 | GGATGTTTGAGGACGCAGAGGAGAAGTTGG |
| HCoV-OC43 | 581 | 24 | GGATGGACCATGGGTTTGTTTCGG |
| HCoV-OC43 | 683 | 32 | GGTGTCTGTCTTGGTGCAGGTCAATTCGTGGG |
| HCoV-OC43 | 824 | 17 | GGACGTGGCGGTTTTGG |
| HCoV-OC43 | 1544 | 17 | GGCTATGGTGGTTTTGG |
| HCoV-OC43 | 1687 | 29 | GGTTTATACTGGAGTTTTAGGTTGCAAGG |
| HCoV-OC43 | 3235 | 29 | GGACACTTTGGATGATGGTCCAAGTGTGG |
| HCoV-OC43 | 4112 | 23 | GGCCATATGGCACATGGTGGTGG |
| HCoV-OC43 | 4944 | 40 | GGTTTGTGCCTGTTGGTGAAAGTTTTGGTAAGAGTCTAGG |
| HCoV-OC43 | 5338 | 30 | GGTTTTGGCCAAAGGTGGGTTTAAATTTGG |
| HCoV-OC43 | 5462 | 31 | GGTGTAAAGCAGGAACAGCGTACTGGTCTGG |
| HCoV-OC43 | 6069 | 30 | GGCCAACAGCTACAGGTGATGTGGTGTTGG |
| HCoV-OC43 | 7260 | 21 | GGAGTTTTAGGTTGCTGGTGG |
| HCoV-OC43 | 7483 | 18 | GGCTAATGGTGGCACTGG |
| HCoV-OC43 | 10031 | 22 | GGTTTATGGTTGGATGACAAGG |
| HCoV-OC43 | 10373 | 34 | GGATCTTGTGGATCTGTTGGTTATGTAATAATGG |
| HCoV-OC43 | 10481 | 34 | GGGGATTTTTATGGTCCTTATAAGGATGCTCAGG |
| HCoV-OC43 | 13262 | 17 | GGATTTTGGCGGGATGG |
| HCoV-OC43 | 13893 | 30 | GGACAAATTGGTGGAGGTAGGCTTAGTAGG |
| HCoV-OC43 | 14281 | 32 | GGGCCTCTTGTTAGGCAAATTTTTGTGGATGG |
| HCoV-OC43 | 16347 | 24 | GGTAATGAATGGTCTGGTTTTTGG |
| HCoV-OC43 | 17100 | 17 | GGCCAAGGTCAGGGTGG |
| HCoV-OC43 | 19254 | 23 | GGATGGCATGGATGCTAAGCAGG |
| HCoV-OC43 | 19507 | 23 | GGTCATTATACAGGACAGGCTGG |
| HCoV-OC43 | 19942 | 23 | GGCGTAGTGGTGGACAAGGTTGG |
| HCoV-OC43 | 20068 | 29 | GGTAATCTGGGGAGTAATGGTAAACCCGG |
| HCoV-OC43 | 20806 | 32 | GGTGCAGGTTCAGAAAAAGGAGTAGCACCGGG |
| HCoV-OC43 | 23213 | 31 | GGTTGTTGATTCGCGGTGGAACAATGCCAGG |
| HCoV-OC43 | 25130 | 38 | GGTTCGTGTGTAGGTAGTGGTCCTGGTAAAAATAATGG |
| HCoV-OC43 | 25523 | 31 | GGTATTTTAGGCCAAGGCATTTTTGTTGAGG |
| HCoV-OC43 | 28847 | 31 | GGTATAAAACTAGGTACTGGCTATTCTTTGG |
| HCoV-OC43 | 29123 | 34 | GGAAATCGTTCTGGTAATGGCATCCTCAAGTGGG |
| HCoV-OC43 | 29587 | 32 | GGCTATTCCGACTAGGTTTCCGCCTGGCACGG |

| name | postion | length | G-quadruplex sequence |
| --- | --- | --- | --- |
| HCoV-HKU1 | 260 | 30 | GGCTGCTTCCGGATGCAGCGGAGGAGTTGG |
| HCoV-HKU1 | 540 | 30 | GGGTGGTTTTCCTAAAGGGTATGTTATGGG |
| HCoV-HKU1 | 643 | 35 | GGTGAAGATTTTTTGGGTTGGATTGTACCTTTTGG |
| HCoV-HKU1 | 1324 | 29 | GGTTGGGTTTCAGGTAATATGATGGATGG |
| HCoV-HKU1 | 4370 | 22 | GGCATATGCTCCATGGTGGTGG |
| HCoV-HKU1 | 5531 | 20 | GGCAGTGGCAGGAAGCATGG |
| HCoV-HKU1 | 7187 | 24 | GGATAAAGGCTACTTTTGGTTTGG |
| HCoV-HKU1 | 18417 | 25 | GGTTGGGTTGGTTTTGATGTTGAGG |
| HCoV-HKU1 | 19757 | 33 | GGTTAATGTTGGTCATTATGATGGACGTACAGG |
| HCoV-HKU1 | 20317 | 37 | GGAGCCCACAAGGTAATCTAGGTGGTAATTGCGCGGG |
| HCoV-HKU1 | 26159 | 33 | GGCTCAGGTTCAGATTGATAGGCTTATTAATGG |

| name | postion | length | G-quadruplex sequence |
| --- | --- | --- | --- |
| HCoV-229E | 607 | 17 | GGTAAGAGAGGTGGTGG |
| HCoV-229E | 1036 | 35 | GGTACCAAGTCTTGGTCTGTTGGTGATTGGACCGG |
| HCoV-229E | 2449 | 20 | GGAGGCAAAATATGGAATGG |
| HCoV-229E | 2866 | 19 | GGCTGTGGTGGTATTTTGG |
| HCoV-229E | 4563 | 30 | GGTTTGTAAGGTTAAGGATTTTGTGTCTGG |
| HCoV-229E | 6616 | 33 | GGTGTTTTTCTAGGTTACAAGGAAACAAATTGG |
| HCoV-229E | 8407 | 41 | GGTGTGTGTTTTGGCTTTGACAAGTGGTTTGTTAACGATGG |
| HCoV-229E | 8461 | 27 | GGTTACGTTTGTGGTACTGGTTTGTGG |
| HCoV-229E | 9172 | 34 | GGTTCTACATTACAGGCTGGTTTGCGCAAAATGG |
| HCoV-229E | 9826 | 23 | GGTTGCACATGGTGGCTTAAAGG |
| HCoV-229E | 9997 | 25 | GGCTTTGGTGGTAAACAAATTTTGG |
| HCoV-229E | 11629 | 31 | GGTGTTGTTTGGACATTACAGGAAGTTAAGG |
| HCoV-229E | 11781 | 30 | GGGCAAGATGAAGGTCAAGGCCACCAAAGG |
| HCoV-229E | 12044 | 28 | GGAGAGGTGCTGTTTTGGGTTACATTGG |
| HCoV-229E | 12232 | 19 | GGTTCTGGTAGCGGTCAGG |
| HCoV-229E | 17319 | 26 | GGTTGGTTAGGAATGGATGTGGAAGG |
| HCoV-229E | 17609 | 24 | GGTGTTTGTACTTTGGGCTGGCGG |
| HCoV-229E | 19403 | 29 | GGGCATCTTAAAGGCAGAGGAGTTTGTGG |
| HCoV-229E | 19871 | 33 | GGGTGCTGGCTCTGATTATGGTGTTGCACCAGG |
| HCoV-229E | 20061 | 23 | GGTAGGACAAAGGCAATTGATGG |
| HCoV-229E | 20816 | 31 | GGTCTGTTTCTGGCTTGCGGTTTACTACTGG |
| HCoV-229E | 20863 | 26 | GGTACTGGGAGAGGTGATTGTAAAGG |
| HCoV-229E | 21824 | 25 | GGAGTGATGGTGATGGAATTACTGG |
| HCoV-229E | 22803 | 30 | GGCCATGTATACAGGTTCTTTAATTGGTGG |
| HCoV-229E | 23442 | 36 | GGATGTTGAAGCGTGGTCTGGGTTGTGCGTTGATGG |
| HCoV-229E | 23909 | 17 | GGCCGTGGTGGGTGTGG |
| HCoV-229E | 25447 | 28 | GGACATAGATTGGCTTCAGGTGTTCAGG |
| HCoV-229E | 26133 | 34 | GGTCTCAGTCGAGGTCGCAGAGTCGCGGTCGTGG |
| HCoV-229E | 26507 | 26 | GGAAGTGCAGGTGTTGTGGCCAATGG |

**Table S5** Conserved G-quadruplxe sequences in bat SARSr-CoV.

| Bat SARS related coronavirus | 353(nsP1-a) |
| --- | --- |
| >KU973692 bat_SARSr_CoV_F46 | GGCTTTGGGGACTCTGTGGAAGAGG |
| >KJ473811 bat_SARSr_CoV_JL2012 | GGTTTCGGGGATACTGTGGAAGAAG |
| >KJ473812 bat_SARSr_CoV_HeB2013 | GGTTTCGGGGATACTGTGGAAGAAG |
| >KJ473814 bat_SARSr_CoV_HuB2013 | GGCTTCGGGGACTCTGTGGAAGAGG |
| >KJ473816 bat_SARSr_CoV_YN2013 | GGCTTCGGGGACTCTGTGGAAGAGG |
| >KJ473815 bat_SARSr_CoV_GX2013 | GGCTTCGGGGACTCTGTGGAAGAGG |
| >KJ473813 bat_SARSr_CoV_SX2013 | GGTTTCGGGGATACTGTGGAAGAAG |
| >KP886808 bat_SARSr_CoV_YNLF_31C | GGTTTCGGGGATACTGTGGAAGAAG |
| >KP886809 bat_SARSr_CoV_YNLF_34C | GGTTTCGGGGATACTGTGGAAGAAG |
| >KF569996 bat_SARSr_CoV_LYRa11 | GGCTTCGGGGACTCTGTGGAAGAGG |
| >KF367457 bat_SARSr_CoV_WIV1 | GGCTTCGGGGACTCTGTGGAAGAGG |
| >KC881006 bat_SARSr_CoV_Rs3367 | GGCTTCGGGGACTCTGTGGAAGAGG |
| >KC881005 bat_SARSr_CoV_RsSHC014 | GGCTTCGGGGACTCTGTGGAAGAGG |
|  | 644(nsP1-b) |
| >KU973692 bat_SARSr_CoV_F46 | GGTAATAAGGGAGCCGGTGG |
| >KJ473811 bat_SARSr_CoV_JL2012 | GGTAATAAGGGAGCTGGTGG |
| >KJ473812 bat_SARSr_CoV_HeB2013 | GGTAATAAGGGAGCTGGTGG |
| >KJ473814 bat_SARSr_CoV_HuB2013 | GGTAATAAGGGAGCTGGCGG |
| >KJ473816 bat_SARSr_CoV_YN2013 | GGTAATAAGGGAGCCGGTGG |
| >KJ473815 bat_SARSr_CoV_GX2013 | GGTAATAAGGGAGCTGGTGG |
| >KJ473813 bat_SARSr_CoV_SX2013 | GGTAATAAGGGAGCTGGTGG |
| >KP886808 bat_SARSr_CoV_YNLF_31C | GGTAATAAGGGAGCCGGTGG |
| >KP886809 bat_SARSr_CoV_YNLF_34C | GGTAATAAGGGAGCCGGTGG |
| >KF569996 bat_SARSr_CoV_LYRa11 | GGTAATAAGGGAGCCGGTGG |
| >KF367457 bat_SARSr_CoV_WIV1 | GGTAATAAGGGAGCCGGTGG |
| >KC881006 bat_SARSr_CoV_Rs3367 | GGTAATAAGGGAGCCGGTGG |
| >KC881005 bat_SARSr_CoV_RsSHC014 | GGTAATAAGGGAGCCGGTGG |
|  | 13385(nsP10) |
| >KU973692 bat_SARSr_CoV_F46 | GGAATGTGGAAAGGTTATGG |
| >KJ473811 bat_SARSr_CoV_JL2012 | GGAATGTGGAAAGGTTATGG |
| >KJ473812 bat_SARSr_CoV_HeB2013 | GGAATGTGGAAAGGTTATGG |
| >KJ473814 bat_SARSr_CoV_HuB2013 | GGGATGTGGAAAGGTTATGG |
| >KJ473816 bat_SARSr_CoV_YN2013 | GGAATGTGGAAAGGTTATGG |
| >KJ473815 bat_SARSr_CoV_GX2013 | GGAATGTGGAAAGGTTATGG |
| >KJ473813 bat_SARSr_CoV_SX2013 | GGAATGTGGAAAGGTTATGG |
| >KP886808 bat_SARSr_CoV_YNLF_31C | GGAATGTGGAAAGGTTATGG |
| >KP886809 bat_SARSr_CoV_YNLF_34C | GGAATGTGGAAAGGTTATGG |
| >KF569996 bat_SARSr_CoV_LYRa11 | GGTATGTGGAAAGGTTATGG |
| >KF367457 bat_SARSr_CoV_WIV1 | GGAATGTGGAAAGGTTATGG |
| >KC881006 bat_SARSr_CoV_Rs3367 | GGAATGTGGAAAGGTTATGG |
| >KC881005 bat_SARSr_CoV_RsSHC014 | GGAATGTGGAAAGGTTATGG |
|  | 24215(S-a) |
| >KU973692 bat_SARSr_CoV_F46 | GGCTGGACATTCGGTGCTGG |
| >KJ473811 bat_SARSr_CoV_JL2012 | GGCTGGACTTTCGGTGCAGG |
| >KJ473812 bat_SARSr_CoV_HeB2013 | GGCTGGACTTTCGGTGCAGG |
| >KJ473814 bat_SARSr_CoV_HuB2013 | GGCTGGACATTTGGTGCAGG |
| >KJ473816 bat_SARSr_CoV_YN2013 | GGCTGGACATTCGGTGCTGG |
| >KJ473815 bat_SARSr_CoV_GX2013 | GGCTGGACATTCGGTGCTGG |
| >KJ473813 bat_SARSr_CoV_SX2013 | GGCTGGACTTTCGGTGCAGG |
| >KP886808 bat_SARSr_CoV_YNLF_31C | GGCTGGACTTTTGGTGCGGG |
| >KP886809 bat_SARSr_CoV_YNLF_34C | GGCTGGACTTTTGGTGCGGG |
| >KF569996 bat_SARSr_CoV_LYRa11 | GGCTGGACATTTGGTGCGGG |
| >KF367457 bat_SARSr_CoV_WIV1 | GGATGGACATTCGGTGCTGG |
| >KC881006 bat_SARSr_CoV_Rs3367 | GGATGGACATTCGGTGCTGG |
| >KC881005 bat_SARSr_CoV_RsSHC014 | GGATGGACATTCGGTGCTGG |
|  | 24268(S-b) |
| >KU973692 bat_SARSr_CoV_F46 | GGCTTATAGGTTTAATGGCATTGG |
| >KJ473811 bat_SARSr_CoV_JL2012 | GGCTTATAGGTTTAATGGCATTGG |
| >KJ473812 bat_SARSr_CoV_HeB2013 | GGCTTATAGGTTTAACGGCATTGG |
| >KJ473814 bat_SARSr_CoV_HuB2013 | GGCATATAGGTTCAATGGCATTGG |
| >KJ473816 bat_SARSr_CoV_YN2013 | GGCATATAGGTTCAATGGCATTGG |
| >KJ473815 bat_SARSr_CoV_GX2013 | GGCATATAGGTTCAATGGCATTGG |
| >KJ473813 bat_SARSr_CoV_SX2013 | GGCTTATAGGTTTAACGGCATTGG |
| >KP886808 bat_SARSr_CoV_YNLF_31C | GGCTTATAGGTTTAATGGCATTGG |
| >KP886809 bat_SARSr_CoV_YNLF_34C | GGCTTATAGGTTTAATGGCATTGG |
| >KF569996 bat_SARSr_CoV_LYRa11 | GGCTTATAGGTTTAATGGCATTGG |
| >KF367457 bat_SARSr_CoV_WIV1 | GGCATATAGGTTCAATGGCATTGG |
| >KC881006 bat_SARSr_CoV_Rs3367 | GGCATATAGGTTCAATGGCATTGG |
| >KC881005 bat_SARSr_CoV_RsSHC014 | GGCATATAGGTTCAATGGCATTGG |
|  | 25197(S-c) |
| >KU973692 bat_SARSr_CoV_F46 | GGCCATGGTACGTTTGGCTCGG |
| >KJ473811 bat_SARSr_CoV_JL2012 | GGCCTTGGTATGTCTGGCTTGG |
| >KJ473812 bat_SARSr_CoV_HeB2013 | GGCCTTGGTATGTCTGGCTTGG |
| >KJ473814 bat_SARSr_CoV_HuB2013 | GGCCTTGGTATGTTTGGCTCGG |
| >KJ473816 bat_SARSr_CoV_YN2013 | GGCCCTGGTACGTTTGGCTCGG |
| >KJ473815 bat_SARSr_CoV_GX2013 | GGCCCTGGTACGTTTGGCTTGG |
| >KJ473813 bat_SARSr_CoV_SX2013 | GGCCTTGGTATGTCTGGCTTGG |
| >KP886808 bat_SARSr_CoV_YNLF_31C | GGCCTTGGTATGTCTGGCTTGG |
| >KP886809 bat_SARSr_CoV_YNLF_34C | GGCCTTGGTATGTCTGGCTTGG |
| >KF569996 bat_SARSr_CoV_LYRa11 | GGCCTTGGTACGTTTGGCTTGG |
| >KF367457 bat_SARSr_CoV_WIV1 | GGCCTTGGTATGTTTGGCTCGG |
| >KC881006 bat_SARSr_CoV_Rs3367 | GGCCTTGGTATGTTTGGCTCGG |
| >KC881005 bat_SARSr_CoV_RsSHC014 | GGCCTTGGTATGTTTGGCTCGG |
|  | 28903(N) |
| >KU973692 bat_SARSr_CoV_F46 | GGCTAGCGGAGGTGG |
| >KJ473811 bat_SARSr_CoV_JL2012 | GGCTAGCGGAGGTGG |
| >KJ473812 bat_SARSr_CoV_HeB2013 | GGCTAGCGGAGGTGG |
| >KJ473814 bat_SARSr_CoV_HuB2013 | GGCTAGCGGAGGTGG |
| >KJ473816 bat_SARSr_CoV_YN2013 | GGCTAGCGGAGGTGG |
| >KJ473815 bat_SARSr_CoV_GX2013 | GGCTAGCGGAGGTGG |
| >KJ473813 bat_SARSr_CoV_SX2013 | GGCTAGCGGAGGTGG |
| >KP886808 bat_SARSr_CoV_YNLF_31C | TGCTAGTGGAGGTGG |
| >KP886809 bat_SARSr_CoV_YNLF_34C | TGCTAGTGGAGGTGG |
| >KF569996 bat_SARSr_CoV_LYRa11 | GGCTAGCGGAGGTGG |
| >KF367457 bat_SARSr_CoV_WIV1 | GGCTAGCGGAGGTGG |
| >KC881006 bat_SARSr_CoV_Rs3367 | GGCTAGCGGAGGTGG |
| >KC881005 bat_SARSr_CoV_RsSHC014 | GGCTAGCGGAGGTGG |

**Table S6** Conserved G-quadruplex sequences in typical SARSr-CoV.

| Typical SARS-related coronavirus | NCBI accession ID | nsP1-a |
| --- | --- | --- |
| SARS-CoV-2 | NC_045512 | GGCTTTGGAGACTCCGTGGAGGAGG |
| SARS-CoV | NC_004718 | GGCTTCGGGGACTCTGTGGAAGAGG |
| Bat_SARSr_CoV_RaTG13 | MN996532 | GGCTTTGGAGACTCCGTGGAGGAGG |
| Bat_SARSr_CoV_WIV1 | KF367457 | GGCTTCGGGGACTCTGTGGAAGAGG |
| Bat_SARSr_CoV_WIV16 | KT444582 | GGCTTCGGGGACTCTGTGGAAGAGG |
| Bat_SARSr_CoV_Rs3367 | KC881006 | GGCTTCGGGGACTCTGTGGAAGAGG |
| Bat_SARSr_CoV_RsSHC014 | KC881005 | GGCTTCGGGGACTCTGTGGAAGAGG |
| Typical SARS-related coronavirus | NCBI accession ID | nsP1-b |
| SARS-CoV-2 | NC_045512 | GGTAATAAAGGAGCTGGTGG |
| SARS-CoV | NC_004718 | GGTAATAAGGGAGCCGGTGG |
| Bat_SARSr_CoV_RaTG13 | MN996532 | GGTAATAAAGGAGCTGGTGG |
| Bat_SARSr_CoV_WIV1 | KF367457 | GGTAATAAGGGAGCCGGTGG |
| Bat_SARSr_CoV_WIV16 | KT444582 | GGTAATAAGGGAGCCGGTGG |
| Bat_SARSr_CoV_Rs3367 | KC881006 | GGTAATAAGGGAGCCGGTGG |
| Bat_SARSr_CoV_RsSHC014 | KC881005 | GGTAATAAGGGAGCCGGTGG |
| Typical SARS-related coronavirus | NCBI accession ID | nsP10 |
| SARS-CoV-2 | NC_045512 | GGTATGTGGAAAGGTTATGG |
| SARS-CoV | NC_004718 | GGAATGTGGAAAGGTTATGG |
| Bat_SARSr_CoV_RaTG13 | MN996532 | GGTATGTGGAAAGGTTATGG |
| Bat_SARSr_CoV_WIV1 | KF367457 | GGAATGTGGAAAGGTTATGG |
| Bat_SARSr_CoV_WIV16 | KT444582 | GGAATGTGGAAAGGTTATGG |
| Bat_SARSr_CoV_Rs3367 | KC881006 | GGAATGTGGAAAGGTTATGG |
| Bat_SARSr_CoV_RsSHC014 | KC881005 | GGAATGTGGAAAGGTTATGG |
| Typical SARS-related coronavirus | NCBI accession ID | S-a |
| SARS-CoV-2 | NC_045512 | GGTTGGACCTTTGGTGCAGG |
| SARS-CoV | NC_004718 | GGATGGACATTTGGTGCTGG |
| Bat_SARSr_CoV_RaTG13 | MN996532 | GGTTGGACTTTTGGTGCAGG |
| Bat_SARSr_CoV_WIV1 | KF367457 | GGATGGACATTCGGTGCTGG |
| Bat_SARSr_CoV_WIV16 | KT444582 | GGATGGACATTCGGTGCTGG |
| Bat_SARSr_CoV_Rs3367 | KC881006 | GGATGGACATTCGGTGCTGG |
| Bat_SARSr_CoV_RsSHC014 | KC881005 | GGATGGACATTCGGTGCTGG |
| Typical SARS-related coronavirus | NCBI accession ID | S-b |
| SARS-CoV-2 | NC_045512 | GGCTTATAGGTTTAATGGTATTGG |
| SARS-CoV | NC_004718 | GGCATATAGGTTCAATGGCATTGG |
| Bat_SARSr_CoV_RaTG13 | MN996532 | GGCTTATAGGTTTAATGGTATTGG |
| Bat_SARSr_CoV_WIV1 | KF367457 | GGCATATAGGTTCAATGGCATTGG |
| Bat_SARSr_CoV_WIV16 | KT444582 | GGCATATAGGTTCAATGGCATTGG |
| Bat_SARSr_CoV_Rs3367 | KC881006 | GGCATATAGGTTCAATGGCATTGG |
| Bat_SARSr_CoV_RsSHC014 | KC881005 | GGCATATAGGTTCAATGGCATTGG |
| Typical SARS-related coronavirus | NCBI accession ID | S-c |
| SARS-CoV-2 | NC_045512 | GGCCATGGTACATTTGGCTAGG |
| SARS-CoV | NC_004718 | GGCCTTGGTATGTTTGGCTCGG |
| Bat_SARSr_CoV_RaTG13 | MN996532 | GGCCATGGTACATTTGGCTAGG |
| Bat_SARSr_CoV_WIV1 | KF367457 | GGCCTTGGTATGTTTGGCTCGG |
| Bat_SARSr_CoV_WIV16 | KT444582 | GGCCTTGGTATGTTTGGCTCGG |
| Bat_SARSr_CoV_Rs3367 | KC881006 | GGCCTTGGTATGTTTGGCTCGG |
| Bat_SARSr_CoV_RsSHC014 | KC881005 | GGCCTTGGTATGTTTGGCTCGG |
| Typical SARS-related coronavirus | NCBI accession ID | N |
| SARS-CoV-2 | NC_045512 | GGCTGGCAATGGCGG |
| SARS-CoV | NC_004718 | GGCTAGCGGAGGTGG |
| Bat_SARSr_CoV_RaTG13 | MN996532 | GGCTGGCAATGGCAG |
| Bat_SARSr_CoV_WIV1 | KF367457 | GGCTAGCGGAGGTGG |
| Bat_SARSr_CoV_WIV16 | KT444582 | GGCTAGCGGAGGTGG |
| Bat_SARSr_CoV_Rs3367 | KC881006 | GGCTAGCGGAGGTGG |
| Bat_SARSr_CoV_RsSHC014 | KC881005 | GGCTAGCGGAGGTGG |

**Table S7** Conserved G-quadruplex sequences in SARS-CoV-2 strains.

| SARS-CoV-2 strains | nsP1-a | nsP1-b | nsP10 | S-a | S-b | S-c | N |
| --- | --- | --- | --- | --- | --- | --- | --- |
| >NC_045512 | GGCTTTGGAGACTCCGTGGAGGAGG | GGTAATAAAGGAGCTGGTGG | GGTATGTGGAAAGGTTATGG | GGTTGGACCTTTGGTGCAGG | GGCTTATAGGTTTAATGGTATTGG | GGCCATGGTACATTTGGCTAGG | GGCTGGCAATGGCGG |
| >MT263381 | GGCTTTGGAGACTCCGTGGAGGAGG | GGTAATAAAGGAGCTGGTGG | GGTATGTGGAAAGGTTATGG | GGTTGGACCTTTGGTGCAGG | GGCTTATAGGTTTAATGGTATTGG | GGCCATGGTACATTTGGCTAGG | GGCTGGCAATGGCGG |
| >MT263382 | GGCTTTGGAGACTCCGTGGAGGAGG | GGTAATAAAGGAGCTGGTGG | GGTATGTGGAAAGGTTATGG | GGTTGGACCTTTGGTGCAGG | GGCTTATAGGTTTAATGGTATTGG | GGCCATGGTACATTTGGCTAGG | GGCTGGCAATGGCGG |
| >MT263383 | GGCTTTGGAGACTCCGTGGAGGAGG | GGTAATAAAGGAGCTGGTGG | GGTATGTGGAAAGGTTATGG | GGTTGGACCTTTGGTGCAGG | GGCTTATAGGTTTAATGGTATTGG | GGCCATGGTACATTTGGCTAGG | GGCTGGCAATGGCGG |
| >MT263384 | GGCTTTGGAGACTCCGTGGAGGAGG | GGTAATAAAGGAGCTGGTGG | GGTATGTGGAAAGGTTATGG | GGTTGGACCTTTGGTGCAGG | GGCTTATAGGTTTAATGGTATTGG | GGCCATGGTACATTTGGCTAGG | GGCTGGCAATGGCGG |
| >MT263386 | GGCTTTGGAGACTCCGTGGAGGAGG | GGTAATAAAGGAGCTGGTGG | GGTATGTGGAAAGGTTATGG | GGTTGGACCTTTGGTGCAGG | GGCTTATAGGTTTAATGGTATTGG | GGCCATGGTACATTTGGCTAGG | GGCTGGCAATGGCGG |
| >MT263388 | GGCTTTGGAGACTCCGTGGAGGAGG | GGTAATAAAGGAGCTGGTGG | GGTATGTGGAAAGGTTATGG | GGTTGGACCTTTGGTGCAGG | GGCTTATAGGTTTAATGGTATTGG | GGCCATGGTACATTTGGCTAGG | GGCTGGCAATGGCGG |
| >MT263390 | GGCTTTGGAGACTCCGTGGAGGAGG | GGTAATAAAGGAGCTGGTGG | GGTATGTGGAAAGGTTATGG | GGTTGGACCTTTGGTGCAGG | GGCTTATAGGTTTAATGGTATTGG | GGCCATGGTACATTTGGCTAGG | GGCTGGCAATGGCGG |
| >MT263391 | GGCTTTGGAGACTCCGTGGAGGAGG | GGTAATAAAGGAGCTGGTGG | GGTATGTGGAAAGGTTATGG | GGTTGGACCTTTGGTGCAGG | GGCTTATAGGTTTAATGGTATTGG | GGCCATGGTACATTTGGCTAGG | GGCTGGCAATGGCGG |
| >MT263392 | GGCTTTGGAGACTCCGTGGAGGAGG | GGTAATAAAGGAGCTGGTGG | GGTATGTGGAAAGGTTATGG | GGTTGGACCTTTGGTGCAGG | GGCTTATAGGTTTAATGGTATTGG | GGCCATGGTACATTTGGCTAGG | GGCTGGCAATGGCGG |
| >MT263394 | GGCTTTGGAGACTCCGTGGAGGAGG | GGTAATAAAGGAGCTGGTGG | GGTATGTGGAAAGGTTATGG | GGTTGGACCTTTGGTGCAGG | GGCTTATAGGTTTAATGGTATTGG | GGCCATGGTACATTTGGCTAGG | GGCTGGCAATGGCGG |
| >MT263395 | GGCTTTGGAGACTCCGTGGAGGAGG | GGTAATAAAGGAGCTGGTGG | GGTATGTGGAAAGGTTATGG | GGTTGGACCTTTGGTGCAGG | GGCTTATAGGTTTAATGGTATTGG | GGCCATGGTACATTTGGCTAGG | GGCTGGCAATGGCGG |
| >MT263396 | GGCTTTGGAGACTCCGTGGAGGAGG | GGTAATAAAGGAGCTGGTGG | GGTATGTGGAAAGGTTATGG | GGTTGGACCTTTGGTGCAGG | GGCTTATAGGTTTAATGGTATTGG | GGCCATGGTACATTTGGCTAGG | GGCTGGCAATGGCGG |
| >MT263398 | GGCTTTGGAGACTCCGTGGAGGAGG | GGTAATAAAGGAGCTGGTGG | GGTATGTGGAAAGGTTATGG | GGTTGGACCTTTGGTGCAGG | GGCTTATAGGTTTAATGGTATTGG | GGCCATGGTACATTTGGCTAGG | GGCTGGCAATGGCGG |
| >MT263399 | GGCTTTGGAGACTCCGTGGAGGAGG | GGTAATAAAGGAGCTGGTGG | GGTATGTGGAAAGGTTATGG | GGTTGGACCTTTGGTGCAGG | GGCTTATAGGTTTAATGGTATTGG | GGCCATGGTACATTTGGCTAGG | GGCTGGCAATGGCGG |
| >MT263400 | GGCTTTGGAGACTCCGTGGAGGAGG | GGTAATAAAGGAGCTGGTGG | GGTATGTGGAAAGGTTATGG | GGTTGGACCTTTGGTGCAGG | GGCTTATAGGTTTAATGGTATTGG | GGCCATGGTACATTTGGCTAGG | GGCTGGCAATGGCGG |
| >MT263441 | GGCTTTGGAGACTCCGTGGAGGAGG | GGTAATAAAGGAGCTGGTGG | GGTATGTGGAAAGGTTATGG | GGTTGGACCTTTGGTGCAGG | GGCTTATAGGTTTAATGGTATTGG | GGCCATGGTACATTTGGCTAGG | GGCTGGCAATGGCGG |
| >MT263442 | GGCTTTGGAGACTCCGTGGAGGAGG | GGTAATAAAGGAGCTGGTGG | GGTATGTGGAAAGGTTATGG | GGTTGGACCTTTGGTGCAGG | GGCTTATAGGTTTAATGGTATTGG | GGCCATGGTACATTTGGCTAGG | GGCTGGCAATGGCGG |
| >MT263443 | GGCTTTGGAGACTCCGTGGAGGAGG | GGTAATAAAGGAGCTGGTGG | GGTATGTGGAAAGGTTATGG | GGTTGGACCTTTGGTGCAGG | GGCTTATAGGTTTAATGGTATTGG | GGCCATGGTACATTTGGCTAGG | GGCTGGCAATGGCGG |
| >MT263444 | GGCTTTGGAGACTCCGTGGAGGAGG | GGTAATAAAGGAGCTGGTGG | GGTATGTGGAAAGGTTATGG | GGTTGGACCTTTGGTGCAGG | GGCTTATAGGTTTAATGGTATTGG | GGCCATGGTACATTTGGCTAGG | GGCTGGCAATGGCGG |
| >MT263445 | GGCTTTGGAGACTCCGTGGAGGAGG | GGTAATAAAGGAGCTGGTGG | GGTATGTGGAAAGGTTATGG | GGTTGGACCTTTGGTGCAGG | GGCTTATAGGTTTAATGGTATTGG | GGCCATGGTACATTTGGCTAGG | GGCTGGCAATGGCGG |
| >MT263446 | GGCTTTGGAGACTCCGTGGAGGAGG | GGTAATAAAGGAGCTGGTGG | GGTATGTGGAAAGGTTATGG | GGTTGGACCTTTGGTGCAGG | GGCTTATAGGTTTAATGGTATTGG | GGCCATGGTACATTTGGCTAGG | GGCTGGCAATGGCGG |
| >MT263447 | GGCTTTGGAGACTCCGTGGAGGAGG | GGTAATAAAGGAGCTGGTGG | GGTATGTGGAAAGGTTATGG | GGTTGGACCTTTGGTGCAGG | GGCTTATAGGTTTAATGGTATTGG | GGCCATGGTACATTTGGCTAGG | GGCTGGCAATGGCGG |
| >MT263448 | GGCTTTGGAGACTCCGTGGAGGAGG | GGTAATAAAGGAGCTGGTGG | GGTATGTGGAAAGGTTATGG | GGTTGGACCTTTGGTGCAGG | GGCTTATAGGTTTAATGGTATTGG | GGCCATGGTACATTTGGCTAGG | GGCTGGCAATGGCGG |
| >MT263449 | GGCTTTGGAGACTCCGTGGAGGAGG | GGTAATAAAGGAGCTGGTGG | GGTATGTGGAAAGGTTATGG | GGTTGGACCTTTGGTGCAGG | GGCTTATAGGTTTAATGGTATTGG | GGCCATGGTACATTTGGCTAGG | GGCTGGCAATGGCGG |
| >MT263450 | GGCTTTGGAGACTCCGTGGAGGAGG | GGTAATAAAGGAGCTGGTGG | GGTATGTGGAAAGGTTATGG | GGTTGGACCTTTGGTGCAGG | GGCTTATAGGTTTAATGGTATTGG | GGCCATGGTACATTTGGCTAGG | GGCTGGCAATGGCGG |
| >MT263451 | GGCTTTGGAGACTCCGTGGAGGAGG | GGTAATAAAGGAGCTGGTGG | GGTATGTGGAAAGGTTATGG | GGTTGGACCTTTGGTGCAGG | GGCTTATAGGTTTAATGGTATTGG | GGCCATGGTACATTTGGCTAGG | GGCTGGCAATGGCGG |
| >MT263452 | GGCTTTGGAGACTCCGTGGAGGAGG | GGTAATAAAGGAGCTGGTGG | GGTATGTGGAAAGGTTATGG | GGTTGGACCTTTGGTGCAGG | GGCTTATAGGTTTAATGGTATTGG | GGCCATGGTACATTTGGCTAGG | GGCTGGCAATGGCGG |
| >MT263453 | GGCTTTGGAGACTCCGTGGAGGAGG | GGTAATAAAGGAGCTGGTGG | GGTATGTGGAAAGGTTATGG | GGTTGGACCTTTGGTGCAGG | GGCTTATAGGTTTAATGGTATTGG | GGCCATGGTACATTTGGCTAGG | GGCTGGCAATGGCGG |
| >MT263454 | GGCTTTGGAGACTCCGTGGAGGAGG | GGTAATAAAGGAGCTGGTGG | GGTATGTGGAAAGGTTATGG | GGTTGGACCTTTGGTGCAGG | GGCTTATAGGTTTAATGGTATTGG | GGCCATGGTACATTTGGCTAGG | GGCTGGCAATGGCGG |
| >MT263455 | GGCTTTGGAGACTCCGTGGAGGAGG | GGTAATAAAGGAGCTGGTGG | GGTATGTGGAAAGGTTATGG | GGTTGGACCTTTGGTGCAGG | GGCTTATAGGTTTAATGGTATTGG | GGCCATGGTACATTTGGCTAGG | GGCTGGCAATGGCGG |
| >MT263456 | GGCTTTGGAGACTCCGTGGAGGAGG | GGTAATAAAGGAGCTGGTGG | GGTATGTGGAAAGGTTATGG | GGTTGGACCTTTGGTGCAGG | GGCTTATAGGTTTAATGGTATTGG | GGCCATGGTACATTTGGCTAGG | GGCTGGCAATGGCGG |
| >MT263457 | GGCTTTGGAGACTCCGTGGAGGAGG | GGTAATAAAGGAGCTGGTGG | GGTATGTGGAAAGGTTATGG | GGTTGGACCTTTGGTGCAGG | GGCTTATAGGTTTAATGGTATTGG | GGCCATGGTACATTTGGCTAGG | GGCTGGCAATGGCGG |
| >MT263458 | GGCTTTGGAGACTCCGTGGAGGAGG | GGTAATAAAGGAGCTGGTGG | GGTATGTGGAAAGGTTATGG | GGTTGGACCTTTGGTGCAGG | GGCTTATAGGTTTAATGGTATTGG | GGCCATGGTACATTTGGCTAGG | GGCTGGCAATGGCGG |
| >MT263402 | GGCTTTGGAGACTCCGTGGAGGAGG | GGTAATAAAGGAGCTGGTGG | GGTATGTGGAAAGGTTATGG | GGTTGGACCTTTGGTGCAGG | GGCTTATAGGTTTAATGGTATTGG | GGCCATGGTACATTTGGCTAGG | GGCTGGCAATGGCGG |
| >MT263403 | GGCTTTGGAGACTCCGTGGAGGAGG | GGTAATAAAGGAGCTGGTGG | GGTATGTGGAAAGGTTATGG | GGTTGGACCTTTGGTGCAGG | GGCTTATAGGTTTAATGGTATTGG | GGCCATGGTACATTTGGCTAGG | GGCTGGCAATGGCGG |
| >MT263404 | GGCTTTGGAGACTCCGTGGAGGAGG | GGTAATAAAGGAGCTGGTGG | GGTATGTGGAAAGGTTATGG | GGTTGGACCTTTGGTGCAGG | GGCTTATAGGTTTAATGGTATTGG | GGCCATGGTACATTTGGCTAGG | GGCTGGCAATGGCGG |
| >MT263405 | GGCTTTGGAGACTCCGTGGAGGAGG | GGTAATAAAGGAGCTGGTGG | GGTATGTGGAAAGGTTATGG | GGTTGGACCTTTGGTGCAGG | GGCTTATAGGTTTAATGGTATTGG | GGCCATGGTACATTTGGCTAGG | GGCTGGCAATGGCGG |
| >MT263410 | GGCTTTGGAGACTCCGTGGAGGAGG | GGTAATAAAGGAGCTGGTGG | GGTATGTGGAAAGGTTATGG | GGTTGGACCTTTGGTGCAGG | GGCTTATAGGTTTAATGGTATTGG | GGCCATGGTACATTTGGCTAGG | GGCTGGCAATGGCGG |
| >MT263413 | GGCTTTGGAGACTCCGTGGAGGAGG | GGTAATAAAGGAGCTGGTGG | GGTATGTGGAAAGGTTATGG | GGTTGGACCTTTGGTGCAGG | GGCTTATAGGTTTAATGGTATTGG | GGCCATGGTACATTTGGCTAGG | GGCTGGCAATGGCGG |
| >MT263414 | GGCTTTGGAGACTCCGTGGAGGAGG | GGTAATAAAGGAGCTGGTGG | GGTATGTGGAAAGGTTATGG | GGTTGGACCTTTGGTGCAGG | GGCTTATAGGTTTAATGGTATTGG | GGCCATGGTACATTTGGCTAGG | GGCTGGCAATGGCGG |
| >MT263416 | GGCTTTGGAGACTCCGTGGAGGAGG | GGTAATAAAGGAGCTGGTGG | GGTATGTGGAAAGGTTATGG | GGTTGGACCTTTGGTGCAGG | GGCTTATAGGTTTAATGGTATTGG | GGCCATGGTACATTTGGCTAGG | GGCTGGCAATGGCGG |
| >MT263419 | GGCTTTGGAGACTCCGTGGAGGAGG | GGTAATAAAGGAGCTGGTGG | GGTATGTGGAAAGGTTATGG | GGTTGGACCTTTGGTGCAGG | GGCTTATAGGTTTAATGGTATTGG | GGCCATGGTACATTTGGCTAGG | GGCTGGCAATGGCGG |
| >MT263421 | GGCTTTGGAGACTCCGTGGAGGAGG | GGTAATAAAGGAGCTGGTGG | GGTATGTGGAAAGGTTATGG | GGTTGGACCTTTGGTGCAGG | GGCTTATAGGTTTAATGGTATTGG | GGCCATGGTACATTTGGCTAGG | GGCTGGCAATGGCGG |
| >MT263424 | GGCTTTGGAGACTCCGTGGAGGAGG | GGTAATAAAGGAGCTGGTGG | GGTATGTGGAAAGGTTATGG | GGTTGGACCTTTGGTGCAGG | GGCTTATAGGTTTAATGGTATTGG | GGCCATGGTACATTTGGCTAGG | GGCTGGCAATGGCGG |
| >MT263425 | GGCTTTGGAGACTCCGTGGAGGAGG | GGTAATAAAGGAGCTGGTGG | GGTATGTGGAAAGGTTATGG | GGTTGGACCTTTGGTGCAGG | GGCTTATAGGTTTAATGGTATTGG | GGCCATGGTACATTTGGCTAGG | GGCTGGCAATGGCGG |
| >MT263429 | GGCTTTGGAGACTCCGTGGAGGAGG | GGTAATAAAGGAGCTGGTGG | GGTATGTGGAAAGGTTATGG | GGTTGGACCTTTGGTGCAGG | GGCTTATAGGTTTAATGGTATTGG | GGCCATGGTACATTTGGCTAGG | GGCTGGCAATGGCGG |
| >MT263431 | GGCTTTGGAGACTCCGTGGAGGAGG | GGTAATAAAGGAGCTGGTGG | GGTATGTGGAAAGGTTATGG | GGTTGGACCTTTGGTGCAGG | GGCTTATAGGTTTAATGGTATTGG | GGCCATGGTACATTTGGCTAGG | GGCTGGCAATGGCGG |
| >MT263432 | GGCTTTGGAGACTCCGTGGAGGAGG | GGTAATAAAGGAGCTGGTGG | GGTATGTGGAAAGGTTATGG | GGTTGGACCTTTGGTGCAGG | GGCTTATAGGTTTAATGGTATTGG | GGCCATGGTACATTTGGCTAGG | GGCTGGCAATGGCGG |
| >MT263435 | GGCTTTGGAGACTCCGTGGAGGAGG | GGTAATAAAGGAGCTGGTGG | GGTATGTGGAAAGGTTATGG | GGTTGGACCTTTGGTGCAGG | GGCTTATAGGTTTAATGGTATTGG | GGCCATGGTACATTTGGCTAGG | GGCTGGCAATGGCGG |
| >MT263438 | GGCTTTGGAGACTCCGTGGAGGAGG | GGTAATAAAGGAGCTGGTGG | GGTATGTGGAAAGGTTATGG | GGTTGGACCTTTGGTGCAGG | GGCTTATAGGTTTAATGGTATTGG | GGCCATGGTACATTTGGCTAGG | GGCTGGCAATGGCGG |
| >MT263440 | GGCTTTGGAGACTCCGTGGAGGAGG | GGTAATAAAGGAGCTGGTGG | GGTATGTGGAAAGGTTATGG | GGTTGGACCTTTGGTGCAGG | GGCTTATAGGTTTAATGGTATTGG | GGCCATGGTACATTTGGCTAGG | GGCTGGCAATGGCGG |
| >MT263462 | GGCTTTGGAGACTCCGTGGAGGAGG | GGTAATAAAGGAGCTGGTGG | GGTATGTGGAAAGGTTATGG | GGTTGGACCTTTGGTGCAGG | GGCTTATAGGTTTAATGGTATTGG | GGCCATGGTACATTTGGCTAGG | GGCTGGCAATGGCGG |
| >MT263463 | GGCTTTGGAGACTCCGTGGAGGAGG | GGTAATAAAGGAGCTGGTGG | GGTATGTGGAAAGGTTATGG | GGTTGGACCTTTGGTGCAGG | GGCTTATAGGTTTAATGGTATTGG | GGCCATGGTACATTTGGCTAGG | GGCTGGCAATGGCGG |
| >MT263464 | GGCTTTGGAGACTCCGTGGAGGAGG | GGTAATAAAGGAGCTGGTGG | GGTATGTGGAAAGGTTATGG | GGTTGGACCTTTGGTGCAGG | GGCTTATAGGTTTAATGGTATTGG | GGCCATGGTACATTTGGCTAGG | GGCTGGCAATGGCGG |
| >MT263465 | GGCTTTGGAGACTCCGTGGAGGAGG | GGTAATAAAGGAGCTGGTGG | GGTATGTGGAAAGGTTATGG | GGTTGGACCTTTGGTGCAGG | GGCTTATAGGTTTAATGGTATTGG | GGCCATGGTACATTTGGCTAGG | GGCTGGCAATGGCGG |
| >MT263468 | GGCTTTGGAGACTCCGTGGAGGAGG | GGTAATAAAGGAGCTGGTGG | GGTATGTGGAAAGGTTATGG | GGTTGGACCTTTGGTGCAGG | GGCTTATAGGTTTAATGGTATTGG | GGCCATGGTACATTTGGCTAGG | GGCTGGCAATGGCGG |
| >MT263469 | GGCTTTGGAGACTCCGTGGAGGAGG | GGTAATAAAGGAGCTGGTGG | GGTATGTGGAAAGGTTATGG | GGTTGGACCTTTGGTGCAGG | GGCTTATAGGTTTAATGGTATTGG | GGCCATGGTACATTTGGCTAGG | GGCTGGCAATGGCGG |
| >MT262896 | GGCTTTGGAGACTCCGTGGAGGAGG | GGTAATAAAGGAGCTGGTGG | GGTATGTGGAAAGGTTATGG | GGTTGGACCTTTGGTGCAGG | GGCTTATAGGTTTAATGGTATTGG | GGCCATGGTACATTTGGCTAGG | GGCTGGCAATGGCGG |
| >MT262897 | GGCTTTGGAGACTCCGTGGAGGAGG | GGTAATAAAGGAGCTGGTGG | GGTATGTGGAAAGGTTATGG | GGTTGGACCTTTGGTGCAGG | GGCTTATAGGTTTAATGGTATTGG | GGCCATGGTACATTTGGCTAGG | GGCTGGCAATGGCGG |
| >MT262898 | GGCTTTGGAGACTCCGTGGAGGAGG | GGTAATAAAGGAGCTGGTGG | GGTATGTGGAAAGGTTATGG | GGTTGGACCTTTGGTGCAGG | GGCTTATAGGTTTAATGGTATTGG | GGCCATGGTACATTTGGCTAGG | GGCTGGCAATGGCGG |
| >MT262899 | GGCTTTGGAGACTCCGTGGAGGAGG | GGTAATAAAGGAGCTGGTGG | GGTATGTGGAAAGGTTATGG | GGTTGGACCTTTGGTGCAGG | GGCTTATAGGTTTAATGGTATTGG | GGCCATGGTACATTTGGCTAGG | GGCTGGCAATGGCGG |
| >MT262900 | GGCTTTGGAGACTCCGTGGAGGAGG | GGTAATAAAGGAGCTGGTGG | GGTATGTGGAAAGGTTATGG | GGTTGGACCTTTGGTGCAGG | GGCTTATAGGTTTAATGGTATTGG | GGCCATGGTACATTTGGCTAGG | GGCTGGCAATGGCGG |
| >MT262901 | GGCTTTGGAGACTCCGTGGAGGAGG | GGTAATAAAGGAGCTGGTGG | GGTATGTGGAAAGGTTATGG | GGTTGGACCTTTGGTGCAGG | GGCTTATAGGTTTAATGGTATTGG | GGCCATGGTACATTTGGCTAGG | GGCTGGCAATGGCGG |
| >MT262902 | GGCTTTGGAGACTCCGTGGAGGAGG | GGTAATAAAGGAGCTGGTGG | GGTATGTGGAAAGGTTATGG | GGTTGGACCTTTGGTGCAGG | GGCTTATAGGTTTAATGGTATTGG | GGCCATGGTACATTTGGCTAGG | GGCTGGCAATGGCGG |
| >MT262903 | GGCTTTGGAGACTCCGTGGAGGAGG | GGTAATAAAGGAGCTGGTGG | GGTATGTGGAAAGGTTATGG | GGTTGGACCTTTGGTGCAGG | GGCTTATAGGTTTAATGGTATTGG | GGCCATGGTACATTTGGCTAGG | GGCTGGCAATGGCGG |
| >MT262904 | GGCTTTGGAGACTCCGTGGAGGAGG | GGTAATAAAGGAGCTGGTGG | GGTATGTGGAAAGGTTATGG | GGTTGGACCTTTGGTGCAGG | GGCTTATAGGTTTAATGGTATTGG | GGCCATGGTACATTTGGCTAGG | GGCTGGCAATGGCGG |
| >MT262905 | GGCTTTGGAGACTCCGTGGAGGAGG | GGTAATAAAGGAGCTGGTGG | GGTATGTGGAAAGGTTATGG | GGTTGGACCTTTGGTGCAGG | GGCTTATAGGTTTAATGGTATTGG | GGCCATGGTACATTTGGCTAGG | GGCTGGCAATGGCGG |
| >MT262906 | GGCTTTGGAGACTCCGTGGAGGAGG | GGTAATAAAGGAGCTGGTGG | GGTATGTGGAAAGGTTATGG | GGTTGGACCTTTGGTGCAGG | GGCTTATAGGTTTAATGGTATTGG | GGCCATGGTACATTTGGCTAGG | GGCTGGCAATGGCGG |
| >MT262911 | GGCTTTGGAGACTCCGTGGAGGAGG | GGTAATAAAGGAGCTGGTGG | GGTATGTGGAAAGGTTATGG | GGTTGGACCTTTGGTGCAGG | GGCTTATAGGTTTAATGGTATTGG | GGCCATGGTACATTTGGCTAGG | GGCTGGCAATGGCGG |
| >MT262912 | GGCTTTGGAGACTCCGTGGAGGAGG | GGTAATAAAGGAGCTGGTGG | GGTATGTGGAAAGGTTATGG | GGTTGGACCTTTGGTGCAGG | GGCTTATAGGTTTAATGGTATTGG | GGCCATGGTACATTTGGCTAGG | GGCTGGCAATGGCGG |
| >MT262913 | GGCTTTGGAGACTCCGTGGAGGAGG | GGTAATAAAGGAGCTGGTGG | GGTATGTGGAAAGGTTATGG | GGTTGGACCTTTGGTGCAGG | GGCTTATAGGTTTAATGGTATTGG | GGCCATGGTACATTTGGCTAGG | GGCTGGCAATGGCGG |
| >MT262993 | GGCTTTGGAGACTCCGTGGAGGAGG | GGTAATAAAGGAGCTGGTGG | GGTATGTGGAAAGGTTATGG | GGTTGGACCTTTGGTGCAGG | GGCTTATAGGTTTAATGGTATTGG | GGCCATGGTACATTTGGCTAGG | GGCTGGCAATGGCGG |
| >MT263074 | GGCTTTGGAGACTCCGTGGAGGAGG | GGTAATAAAGGAGCTGGTGG | GGTATGTGGAAAGGTTATGG | GGTTGGACCTTTGGTGCAGG | GGCTTATAGGTTTAATGGTATTGG | GGCCATGGTACATTTGGCTAGG | GGCTGGCAATGGCGG |
| >MT262909 | GGCTTTGGAGACTCCGTGGAGGAGG | GGTAATAAAGGAGCTGGTGG | GGTATGTGGAAAGGTTATGG | GGTTGGACCTTTGGTGCAGG | GGCTTATAGGTTTAATGGTATTGG | GGCCATGGTACATTTGGCTAGG | GGCTGGCAATGGCGG |
| >MT262910 | GGCTTTGGAGACTCCGTGGAGGAGG | GGTAATAAAGGAGCTGGTGG | GGTATGTGGAAAGGTTATGG | GGTTGGACCTTTGGTGCAGG | GGCTTATAGGTTTAATGGTATTGG | GGCCATGGTACATTTGGCTAGG | GGCTGGCAATGGCGG |
| >MT262907 | GGCTTTGGAGACTCCGTGGAGGAGG | GGTAATAAAGGAGCTGGTGG | GGTATGTGGAAAGGTTATGG | GGTTGGACCTTTGGTGCAGG | GGCTTATAGGTTTAATGGTATTGG | GGCCATGGTACATTTGGCTAGG | GGCTGGCAATGGCGG |
| >MT262908 | GGCTTTGGAGACTCCGTGGAGGAGG | GGTAATAAAGGAGCTGGTGG | GGTATGTGGAAAGGTTATGG | GGTTGGACCTTTGGTGCAGG | GGCTTATAGGTTTAATGGTATTGG | GGCCATGGTACATTTGGCTAGG | GGCTGGCAATGGCGG |
| >MT263406 | GGCTTTGGAGACTCCGTGGAGGAGG | GGTAATAAAGGAGCTGGTGG | GGTATGTGGAAAGGTTATGG | GGTTGGACCTTTGGTGCAGG | GGCTTATAGGTTTAATGGTATTGG | GGCCATGGTACATTTGGCTAGG | GGCTGGCAATGGCGG |
| >MT263408 | GGCTTTGGAGACTCCGTGGAGGAGG | GGTAATAAAGGAGCTGGTGG | GGTATGTGGAAAGGTTATGG | GGTTGGACCTTTGGTGCAGG | GGCTTATAGGTTTAATGGTATTGG | GGCCATGGTACATTTGGCTAGG | GGCTGGCAATGGCGG |
| >MT263411 | GGCTTTGGAGACTCCGTGGAGGAGG | GGTAATAAAGGAGCTGGTGG | GGTATGTGGAAAGGTTATGG | GGTTGGACCTTTGGTGCAGG | GGCTTATAGGTTTAATGGTATTGG | GGCCATGGTACATTTGGCTAGG | GGCTGGCAATGGCGG |
| >MT263412 | GGCTTTGGAGACTCCGTGGAGGAGG | GGTAATAAAGGAGCTGGTGG | GGTATGTGGAAAGGTTATGG | GGTTGGACCTTTGGTGCAGG | GGCTTATAGGTTTAATGGTATTGG | GGCCATGGTACATTTGGCTAGG | GGCTGGCAATGGCGG |
| >MT263415 | GGCTTTGGAGACTCCGTGGAGGAGG | GGTAATAAAGGAGCTGGTGG | GGTATGTGGAAAGGTTATGG | GGTTGGACCTTTGGTGCAGG | GGCTTATAGGTTTAATGGTATTGG | GGCCATGGTACATTTGGCTAGG | GGCTGGCAATGGCGG |
| >MT263417 | GGCTTTGGAGACTCCGTGGAGGAGG | GGTAATAAAGGAGCTGGTGG | GGTATGTGGAAAGGTTATGG | GGTTGGACCTTTGGTGCAGG | GGCTTATAGGTTTAATGGTATTGG | GGCCATGGTACATTTGGCTAGG | GGCTGGCAATGGCGG |
| >MT263418 | GGCTTTGGAGACTCCGTGGAGGAGG | GGTAATAAAGGAGCTGGTGG | GGTATGTGGAAAGGTTATGG | GGTTGGACCTTTGGTGCAGG | GGCTTATAGGTTTAATGGTATTGG | GGCCATGGTACATTTGGCTAGG | GGCTGGCAATGGCGG |
| >MT263420 | GGCTTTGGAGACTCCGTGGAGGAGG | GGTAATAAAGGAGCTGGTGG | GGTATGTGGAAAGGTTATGG | GGTTGGACCTTTGGTGCAGG | GGCTTATAGGTTTAATGGTATTGG | GGCCATGGTACATTTGGCTAGG | GGCTGGCAATGGCGG |
| >MT263422 | GGCTTTGGAGACTCCGTGGAGGAGG | GGTAATAAAGGAGCTGGTGG | GGTATGTGGAAAGGTTATGG | GGTTGGACCTTTGGTGCAGG | GGCTTATAGGTTTAATGGTATTGG | GGCCATGGTACATTTGGCTAGG | GGCTGGCAATGGCGG |
| >MT263423 | GGCTTTGGAGACTCCGTGGAGGAGG | GGTAATAAAGGAGCTGGTGG | GGTATGTGGAAAGGTTATGG | GGTTGGACCTTTGGTGCAGG | GGCTTATAGGTTTAATGGTATTGG | GGCCATGGTACATTTGGCTAGG | GGCTGGCAATGGCGG |
| >MT263428 | GGCTTTGGAGACTCCGTGGAGGAGG | GGTAATAAAGGAGCTGGTGG | GGTATGTGGAAAGGTTATGG | GGTTGGACCTTTGGTGCAGG | GGCTTATAGGTTTAATGGTATTGG | GGCCATGGTACATTTGGCTAGG | GGCTGGCAATGGCGG |
| >MT263430 | GGCTTTGGAGACTCCGTGGAGGAGG | GGTAATAAAGGAGCTGGTGG | GGTATGTGGAAAGGTTATGG | GGTTGGACCTTTGGTGCAGG | GGCTTATAGGTTTAATGGTATTGG | GGCCATGGTACATTTGGCTAGG | GGCTGGCAATGGCGG |
| >MT263433 | GGCTTTGGAGACTCCGTGGAGGAGG | GGTAATAAAGGAGCTGGTGG | GGTATGTGGAAAGGTTATGG | GGTTGGACCTTTGGTGCAGG | GGCTTATAGGTTTAATGGTATTGG | GGCCATGGTACATTTGGCTAGG | GGCTGGCAATGGCGG |
| >MT263434 | GGCTTTGGAGACTCCGTGGAGGAGG | GGTAATAAAGGAGCTGGTGG | GGTATGTGGAAAGGTTATGG | GGTTGGACCTTTGGTGCAGG | GGCTTATAGGTTTAATGGTATTGG | GGCCATGGTACATTTGGCTAGG | GGCTGGCAATGGCGG |
| >MT263436 | GGCTTTGGAGACTCCGTGGAGGAGG | GGTAATAAAGGAGCTGGTGG | GGTATGTGGAAAGGTTATGG | GGTTGGACCTTTGGTGCAGG | GGCTTATAGGTTTAATGGTATTGG | GGCCATGGTACATTTGGCTAGG | GGCTGGCAATGGCGG |
| >MT263437 | GGCTTTGGAGACTCCGTGGAGGAGG | GGTAATAAAGGAGCTGGTGG | GGTATGTGGAAAGGTTATGG | GGTTGGACCTTTGGTGCAGG | GGCTTATAGGTTTAATGGTATTGG | GGCCATGGTACATTTGGCTAGG | GGCTGGCAATGGCGG |
| >MT263439 | GGCTTTGGAGACTCCGTGGAGGAGG | GGTAATAAAGGAGCTGGTGG | GGTATGTGGAAAGGTTATGG | GGTTGGACCTTTGGTGCAGG | GGCTTATAGGTTTAATGGTATTGG | GGCCATGGTACATTTGGCTAGG | GGCTGGCAATGGCGG |
| >MT263459 | GGCTTTGGAGACTCCGTGGAGGAGG | GGTAATAAAGGAGCTGGTGG | GGTATGTGGAAAGGTTATGG | GGTTGGACCTTTGGTGCAGG | GGCTTATAGGTTTAATGGTATTGG | GGCCATGGTACATTTGGCTAGG | GGCTGGCAATGGCGG |
| >MT263467 | GGCTTTGGAGACTCCGTGGAGGAGG | GGTAATAAAGGAGCTGGTGG | GGTATGTGGAAAGGTTATGG | GGTTGGACCTTTGGTGCAGG | GGCTTATAGGTTTAATGGTATTGG | GGCCATGGTACATTTGGCTAGG | GGCTGGCAATGGCGG |
| >MT262916 | GGCTTTGGAGACTCCGTGGAGGAGG | GGTAATAAAGGAGCTGGTGG | GGTATGTGGAAAGGTTATGG | GGTTGGACCTTTGGTGCAGG | GGCTTATAGGTTTAATGGTATTGG | GGCCATGGTACATTTGGCTAGG | GGCTGGCAATGGCGG |
| >MT262915 | GGCTTTGGAGACTCCGTGGAGGAGG | GGTAATAAAGGAGCTGGTGG | GGTATGTGGAAAGGTTATGG | GGTTGGACCTTTGGTGCAGG | GGCTTATAGGTTTAATGGTATTGG | GGCCATGGTACATTTGGCTAGG | GGCTGGCAATGGCGG |
| >MT262914 | GGCTTTGGAGACTCCGTGGAGGAGG | GGTAATAAAGGAGCTGGTGG | GGTATGTGGAAAGGTTATGG | GGTTGGACCTTTGGTGCAGG | GGCTTATAGGTTTAATGGTATTGG | GGCCATGGTACATTTGGCTAGG | GGCTGGCAATGGCGG |
| >MT259237 | GGCTTTGGAGACTCCGTGGAGGAGG | GGTAATAAAGGAGCTGGTGG | GGTATGTGGAAAGGTTATGG | GGTTGGACCTTTGGTGCAGG | GGCTTATAGGTTTAATGGTATTGG | GGCCATGGTACATTTGGCTAGG | GGCTGGCAATGGCGG |
| >MT259260 | GGCTTTGGAGACTCCGTGGAGGAGG | GGTAATAAAGGAGCTGGTGG | GGTATGTGGAAAGGTTATGG | GGTTGGACCTTTGGTGCAGG | GGCTTATAGGTTTAATGGTATTGG | GGCCATGGTACATTTGGCTAGG | GGCTGGCAATGGCGG |
| >MT259261 | GGCTTTGGAGACTCCGTGGAGGAGG | GGTAATAAAGGAGCTGGTGG | GGTATGTGGAAAGGTTATGG | GGTTGGACCTTTGGTGCAGG | GGCTTATAGGTTTAATGGTATTGG | GGCCATGGTACATTTGGCTAGG | GGCTGGCAATGGCGG |
| >MT259271 | GGCTTTGGAGACTCCGTGGAGGAGG | GGTAATAAAGGAGCTGGTGG | GGTATGTGGAAAGGTTATGG | GGTTGGACCTTTGGTGCAGG | GGCTTATAGGTTTAATGGTATTGG | GGCCATGGTACATTTGGCTAGG | GGCTGGCAATGGCGG |
| >MT259280 | GGCTTTGGAGACTCCGTGGAGGAGG | GGTAATAAAGGAGCTGGTGG | GGTATGTGGAAAGGTTATGG | GGTTGGACCTTTGGTGCAGG | GGCTTATAGGTTTAATGGTATTGG | GGCCATGGTACATTTGGCTAGG | GGCTGGCAATGGCGG |
| >MT259241 | GGCTTTGGAGACTCCGTGGAGGAGG | GGTAATAAAGGAGCTGGTGG | GGTATGTGGAAAGGTTATGG | GGTTGGACCTTTGGTGCAGG | GGCTTATAGGTTTAATGGTATTGG | GGCCATGGTACATTTGGCTAGG | GGCTGGCAATGGCGG |
| >MT259247 | GGCTTTGGAGACTCCGTGGAGGAGG | GGTAATAAAGGAGCTGGTGG | GGTATGTGGAAAGGTTATGG | GGTTGGACCTTTGGTGCAGG | GGCTTATAGGTTTAATGGTATTGG | GGCCATGGTACATTTGGCTAGG | GGCTGGCAATGGCGG |
| >MT259252 | GGCTTTGGAGACTCCGTGGAGGAGG | GGTAATAAAGGAGCTGGTGG | GGTATGTGGAAAGGTTATGG | GGTTGGACCTTTGGTGCAGG | GGCTTATAGGTTTAATGGTATTGG | GGCCATGGTACATTTGGCTAGG | GGCTGGCAATGGCGG |
| >MT259256 | GGCTTTGGAGACTCCGTGGAGGAGG | GGTAATAAAGGAGCTGGTGG | GGTATGTGGAAAGGTTATGG | GGTTGGACCTTTGGTGCAGG | GGCTTATAGGTTTAATGGTATTGG | GGCCATGGTACATTTGGCTAGG | GGCTGGCAATGGCGG |
| >MT259263 | GGCTTTGGAGACTCCGTGGAGGAGG | GGTAATAAAGGAGCTGGTGG | GGTATGTGGAAAGGTTATGG | GGTTGGACCTTTGGTGCAGG | GGCTTATAGGTTTAATGGTATTGG | GGCCATGGTACATTTGGCTAGG | GGCTGGCAATGGCGG |
| >MT259264 | GGCTTTGGAGACTCCGTGGAGGAGG | GGTAATAAAGGAGCTGGTGG | GGTATGTGGAAAGGTTATGG | GGTTGGACCTTTGGTGCAGG | GGCTTATAGGTTTAATGGTATTGG | GGCCATGGTACATTTGGCTAGG | GGCTGGCAATGGCGG |
| >MT259274 | GGCTTTGGAGACTCCGTGGAGGAGG | GGTAATAAAGGAGCTGGTGG | GGTATGTGGAAAGGTTATGG | GGTTGGACCTTTGGTGCAGG | GGCTTATAGGTTTAATGGTATTGG | GGCCATGGTACATTTGGCTAGG | GGCTGGCAATGGCGG |
| >MT259275 | GGCTTTGGAGACTCCGTGGAGGAGG | GGTAATAAAGGAGCTGGTGG | GGTATGTGGAAAGGTTATGG | GGTTGGACCTTTGGTGCAGG | GGCTTATAGGTTTAATGGTATTGG | GGCCATGGTACATTTGGCTAGG | GGCTGGCAATGGCGG |
| >MT259251 | GGCTTTGGAGACTCCGTGGAGGAGG | GGTAATAAAGGAGCTGGTGG | GGTATGTGGAAAGGTTATGG | GGTTGGACCTTTGGTGCAGG | GGCTTATAGGTTTAATGGTATTGG | GGCCATGGTACATTTGGCTAGG | GGCTGGCAATGGCGG |
| >MT259235 | GGCTTTGGAGACTCCGTGGAGGAGG | GGTAATAAAGGAGCTGGTGG | GGTATGTGGAAAGGTTATGG | GGTTGGACCTTTGGTGCAGG | GGCTTATAGGTTTAATGGTATTGG | GGCCATGGTACATTTGGCTAGG | GGCTGGCAATGGCGG |
| >MT259239 | GGCTTTGGAGACTCCGTGGAGGAGG | GGTAATAAAGGAGCTGGTGG | GGTATGTGGAAAGGTTATGG | GGTTGGACCTTTGGTGCAGG | GGCTTATAGGTTTAATGGTATTGG | GGCCATGGTACATTTGGCTAGG | GGCTGGCAATGGCGG |
| >MT259249 | GGCTTTGGAGACTCCGTGGAGGAGG | GGTAATAAAGGAGCTGGTGG | GGTATGTGGAAAGGTTATGG | GGTTGGACCTTTGGTGCAGG | GGCTTATAGGTTTAATGGTATTGG | GGCCATGGTACATTTGGCTAGG | GGCTGGCAATGGCGG |
| >MT259282 | GGCTTTGGAGACTCCGTGGAGGAGG | GGTAATAAAGGAGCTGGTGG | GGTATGTGGAAAGGTTATGG | GGTTGGACCTTTGGTGCAGG | GGCTTATAGGTTTAATGGTATTGG | GGCCATGGTACATTTGGCTAGG | GGCTGGCAATGGCGG |
| >MT258381 | GGCTTTGGAGACTCCGTGGAGGAGG | GGTAATAAAGGAGCTGGTGG | GGTATGTGGAAAGGTTATGG | GGTTGGACCTTTGGTGCAGG | GGCTTATAGGTTTAATGGTATTGG | GGCCATGGTACATTTGGCTAGG | GGCTGGCAATGGCGG |
| >MT258383 | GGCTTTGGAGACTCCGTGGAGGAGG | GGTAATAAAGGAGCTGGTGG | GGTATGTGGAAAGGTTATGG | GGTTGGACCTTTGGTGCAGG | GGCTTATAGGTTTAATGGTATTGG | GGCCATGGTACATTTGGCTAGG | GGCTGGCAATGGCGG |
| >MT259250 | GGCTTTGGAGACTCCGTGGAGGAGG | GGTAATAAAGGAGCTGGTGG | GGTATGTGGAAAGGTTATGG | GGTTGGACCTTTGGTGCAGG | GGCTTATAGGTTTAATGGTATTGG | GGCCATGGTACATTTGGCTAGG | GGCTGGCAATGGCGG |
| >MT259269 | GGCTTTGGAGACTCCGTGGAGGAGG | GGTAATAAAGGAGCTGGTGG | GGTATGTGGAAAGGTTATGG | GGTTGGACCTTTGGTGCAGG | GGCTTATAGGTTTAATGGTATTGG | GGCCATGGTACATTTGGCTAGG | GGCTGGCAATGGCGG |
| >MT258377 | GGCTTTGGAGACTCCGTGGAGGAGG | GGTAATAAAGGAGCTGGTGG | GGTATGTGGAAAGGTTATGG | GGTTGGACCTTTGGTGCAGG | GGCTTATAGGTTTAATGGTATTGG | GGCCATGGTACATTTGGCTAGG | GGCTGGCAATGGCGG |
| >MT259236 | GGCTTTGGAGACTCCGTGGAGGAGG | GGTAATAAAGGAGCTGGTGG | GGTATGTGGAAAGGTTATGG | GGTTGGACCTTTGGTGCAGG | GGCTTATAGGTTTAATGGTATTGG | GGCCATGGTACATTTGGCTAGG | GGCTGGCAATGGCGG |
| >MT259281 | GGCTTTGGAGACTCCGTGGAGGAGG | GGTAATAAAGGAGCTGGTGG | GGTATGTGGAAAGGTTATGG | GGTTGGACCTTTGGTGCAGG | GGCTTATAGGTTTAATGGTATTGG | GGCCATGGTACATTTGGCTAGG | GGCTGGCAATGGCGG |
| >MT258380 | GGCTTTGGAGACTCCGTGGAGGAGG | GGTAATAAAGGAGCTGGTGG | GGTATGTGGAAAGGTTATGG | GGTTGGACCTTTGGTGCAGG | GGCTTATAGGTTTAATGGTATTGG | GGCCATGGTACATTTGGCTAGG | GGCTGGCAATGGCGG |
| >MT258379 | GGCTTTGGAGACTCCGTGGAGGAGG | GGTAATAAAGGAGCTGGTGG | GGTATGTGGAAAGGTTATGG | GGTTGGACCTTTGGTGCAGG | GGCTTATAGGTTTAATGGTATTGG | GGCCATGGTACATTTGGCTAGG | GGCTGGCAATGGCGG |
| >MT259245 | GGCTTTGGAGACTCCGTGGAGGAGG | GGTAATAAAGGAGCTGGTGG | GGTATGTGGAAAGGTTATGG | GGTTGGACCTTTGGTGCAGG | GGCTTATAGGTTTAATGGTATTGG | GGCCATGGTACATTTGGCTAGG | GGCTGGCAATGGCGG |
| >MT259254 | GGCTTTGGAGACTCCGTGGAGGAGG | GGTAATAAAGGAGCTGGTGG | GGTATGTGGAAAGGTTATGG | GGTTGGACCTTTGGTGCAGG | GGCTTATAGGTTTAATGGTATTGG | GGCCATGGTACATTTGGCTAGG | GGCTGGCAATGGCGG |
| >MT259257 | GGCTTTGGAGACTCCGTGGAGGAGG | GGTAATAAAGGAGCTGGTGG | GGTATGTGGAAAGGTTATGG | GGTTGGACCTTTGGTGCAGG | GGCTTATAGGTTTAATGGTATTGG | GGCCATGGTACATTTGGCTAGG | GGCTGGCAATGGCGG |
| >MT259266 | GGCTTTGGAGACTCCGTGGAGGAGG | GGTAATAAAGGAGCTGGTGG | GGTATGTGGAAAGGTTATGG | GGTTGGACCTTTGGTGCAGG | GGCTTATAGGTTTAATGGTATTGG | GGCCATGGTACATTTGGCTAGG | GGCTGGCAATGGCGG |
| >MT259268 | GGCTTTGGAGACTCCGTGGAGGAGG | GGTAATAAAGGAGCTGGTGG | GGTATGTGGAAAGGTTATGG | GGTTGGACCTTTGGTGCAGG | GGCTTATAGGTTTAATGGTATTGG | GGCCATGGTACATTTGGCTAGG | GGCTGGCAATGGCGG |
| >MT259285 | GGCTTTGGAGACTCCGTGGAGGAGG | GGTAATAAAGGAGCTGGTGG | GGTATGTGGAAAGGTTATGG | GGTTGGACCTTTGGTGCAGG | GGCTTATAGGTTTAATGGTATTGG | GGCCATGGTACATTTGGCTAGG | GGCTGGCAATGGCGG |
| >MT259286 | GGCTTTGGAGACTCCGTGGAGGAGG | GGTAATAAAGGAGCTGGTGG | GGTATGTGGAAAGGTTATGG | GGTTGGACCTTTGGTGCAGG | GGCTTATAGGTTTAATGGTATTGG | GGCCATGGTACATTTGGCTAGG | GGCTGGCAATGGCGG |
| >MT259287 | GGCTTTGGAGACTCCGTGGAGGAGG | GGTAATAAAGGAGCTGGTGG | GGTATGTGGAAAGGTTATGG | GGTTGGACCTTTGGTGCAGG | GGCTTATAGGTTTAATGGTATTGG | GGCCATGGTACATTTGGCTAGG | GGCTGGCAATGGCGG |
| >MT258378 | GGCTTTGGAGACTCCGTGGAGGAGG | GGTAATAAAGGAGCTGGTGG | GGTATGTGGAAAGGTTATGG | GGTTGGACCTTTGGTGCAGG | GGCTTATAGGTTTAATGGTATTGG | GGCCATGGTACATTTGGCTAGG | GGCTGGCAATGGCGG |
| >MT259243 | GGCTTTGGAGACTCCGTGGAGGAGG | GGTAATAAAGGAGCTGGTGG | GGTATGTGGAAAGGTTATGG | GGTTGGACCTTTGGTGCAGG | GGCTTATAGGTTTAATGGTATTGG | GGCCATGGTACATTTGGCTAGG | GGCTGGCAATGGCGG |
| >MT259244 | GGCTTTGGAGACTCCGTGGAGGAGG | GGTAATAAAGGAGCTGGTGG | GGTATGTGGAAAGGTTATGG | GGTTGGACCTTTGGTGCAGG | GGCTTATAGGTTTAATGGTATTGG | GGCCATGGTACATTTGGCTAGG | GGCTGGCAATGGCGG |
| >MT259246 | GGCTTTGGAGACTCCGTGGAGGAGG | GGTAATAAAGGAGCTGGTGG | GGTATGTGGAAAGGTTATGG | GGTTGGACCTTTGGTGCAGG | GGCTTATAGGTTTAATGGTATTGG | GGCCATGGTACATTTGGCTAGG | GGCTGGCAATGGCGG |
| >MT259248 | GGCTTTGGAGACTCCGTGGAGGAGG | GGTAATAAAGGAGCTGGTGG | GGTATGTGGAAAGGTTATGG | GGTTGGACCTTTGGTGCAGG | GGCTTATAGGTTTAATGGTATTGG | GGCCATGGTACATTTGGCTAGG | GGCTGGCAATGGCGG |
| >MT259253 | GGCTTTGGAGACTCCGTGGAGGAGG | GGTAATAAAGGAGCTGGTGG | GGTATGTGGAAAGGTTATGG | GGTTGGACCTTTGGTGCAGG | GGCTTATAGGTTTAATGGTATTGG | GGCCATGGTACATTTGGCTAGG | GGCTGGCAATGGCGG |
| >MT259258 | GGCTTTGGAGACTCCGTGGAGGAGG | GGTAATAAAGGAGCTGGTGG | GGTATGTGGAAAGGTTATGG | GGTTGGACCTTTGGTGCAGG | GGCTTATAGGTTTAATGGTATTGG | GGCCATGGTACATTTGGCTAGG | GGCTGGCAATGGCGG |
| >MT259267 | GGCTTTGGAGACTCCGTGGAGGAGG | GGTAATAAAGGAGCTGGTGG | GGTATGTGGAAAGGTTATGG | GGTTGGACCTTTGGTGCAGG | GGCTTATAGGTTTAATGGTATTGG | GGCCATGGTACATTTGGCTAGG | GGCTGGCAATGGTGG |
| >MT259273 | GGCTTTGGAGACTCCGTGGAGGAGG | GGTAATAAAGGAGCTGGTGG | GGTATGTGGAAAGGTTATGG | GGTTGGACCTTTGGTGCAGG | GGCTTATAGGTTTAATGGTATTGG | GGCCATGGTACATTTGGCTAGG | GGCTGGCAATGGCGG |
| >MT259277 | GGCTTTGGAGACTCCGTGGAGGAGG | GGTAATAAAGGAGCTGGTGG | GGTATGTGGAAAGGTTATGG | GGTTGGACCTTTGGTGCAGG | GGCTTATAGGTTTAATGGTATTGG | GGCCATGGTACATTTGGCTAGG | GGCTGGCAATGGCGG |
| >MT259278 | GGCTTTGGAGACTCCGTGGAGGAGG | GGTAATAAAGGAGCTGGTGG | GGTATGTGGAAAGGTTATGG | GGTTGGACCTTTGGTGCAGG | GGCTTATAGGTTTAATGGTATTGG | GGCCATGGTACATTTGGCTAGG | GGCTGGCAATGGCGG |
| >MT259226 | GGCTTTGGAGACTCCGTGGAGGAGG | GGTAATAAAGGAGCTGGTGG | GGTATGTGGAAAGGTTATGG | GGTTGGACCTTTGGTGCAGG | GGCTTATAGGTTTAATGGTATTGG | GGCCATGGTACATTTGGCTAGG | GGCTGGCAATGGCGG |
| >MT259227 | GGCTTTGGAGACTCCGTGGAGGAGG | GGTAATAAAGGAGCTGGTGG | GGTATGTGGAAAGGTTATGG | GGTTGGACCTTTGGTGCAGG | GGCTTATAGGTTTAATGGTATTGG | GGCCATGGTACATTTGGCTAGG | GGCTGGCAATGGCGG |
| >MT259228 | GGCTTTGGAGACTCCGTGGAGGAGG | GGTAATAAAGGAGCTGGTGG | GGTATGTGGAAAGGTTATGG | GGTTGGACCTTTGGTGCAGG | GGCTTATAGGTTTAATGGTATTGG | GGCCATGGTACATTTGGCTAGG | GGCTGGCAATGGCGG |
| >MT259229 | GGCTTTGGAGACTCCGTGGAGGAGG | GGTAATAAAGGAGCTGGTGG | GGTATGTGGAAAGGTTATGG | GGTTGGACCTTTGGTGCAGG | GGCTTATAGGTTTAATGGTATTGG | GGCCATGGTACATTTGGCTAGG | GGCTGGCAATGGCGG |
| >MT259230 | GGCTTTGGAGACTCCGTGGAGGAGG | GGTAATAAAGGAGCTGGTGG | GGTATGTGGAAAGGTTATGG | GGTTGGACCTTTGGTGCAGG | GGCTTATAGGTTTAATGGTATTGG | GGCCATGGTACATTTGGCTAGG | GGCTGGCAATGGCGG |
| >MT259231 | GGCTTTGGAGACTCCGTGGAGGAGG | GGTAATAAAGGAGCTGGTGG | GGTATGTGGAAAGGTTATGG | GGTTGGACCTTTGGTGCAGG | GGCTTATAGGTTTAATGGTATTGG | GGCCATGGTACATTTGGCTAGG | GGCTGGCAATGGCGG |
| >MT251977 | GGCTTTGGAGACTCCGTGGAGGAGG | GGTAATAAAGGAGCTGGTGG | GGTATGTGGAAAGGTTATGG | GGTTGGACCTTTGGTGCAGG | GGCTTATAGGTTTAATGGTATTGG | GGCCATGGTACATTTGGCTAGG | GGCTGGCAATGGCGG |
| >MT251976 | GGCTTTGGAGACTCCGTGGAGGAGG | GGTAATAAAGGAGCTGGTGG | GGTATGTGGAAAGGTTATGG | GGTTGGACCTTTGGTGCAGG | GGCTTATAGGTTTAATGGTATTGG | GGCCATGGTACATTTGGCTAGG | GGCTGGCAATGGCGG |
| >MT251973 | GGCTTTGGAGACTCCGTGGAGGAGG | GGTAATAAAGGAGCTGGTGG | GGTATGTGGAAAGGTTATGG | GGTTGGACCTTTGGTGCAGG | GGCTTATAGGTTTAATGGTATTGG | GGCCATGGTACATTTGGCTAGG | GGCTGGCAATGGCGG |
| >MT251972 | GGCTTTGGAGACTCCGTGGAGGAGG | GGTAATAAAGGAGCTGGTGG | GGTATGTGGAAAGGTTATGG | GGTTGGACCTTTGGTGCAGG | GGCTTATAGGTTTAATGGTATTGG | GGCCATGGTACATTTGGCTAGG | GGCTGGCAATGGCGG |
| >MT253702 | GGCTTTGGAGACTCCGTGGAGGAGG | GGTAATAAAGGAGCTGGTGG | GGTATGTGGAAAGGTTATGG | GGTTGGACCTTTGGTGCAGG | GGCTTATAGGTTTAATGGTATTGG | GGCCATGGTACATTTGGCTAGG | GGCTGGCAATGGCGG |
| >MT253704 | GGCTTTGGAGACTCCGTGGAGGAGG | GGTAATAAAGGAGCTGGTGG | GGTATGTGGAAAGGTTATGG | GGTTGGACCTTTGGTGCAGG | GGCTTATAGGTTTAATGGTATTGG | GGCCATGGTACATTTGGCTAGG | GGCTGGCAATGGCGG |
| >MT253706 | GGCTTTGGAGACTCCGTGGAGGAGG | GGTAATAAAGGAGCTGGTGG | GGTATGTGGAAAGGTTATGG | GGTTGGACCTTTGGTGCAGG | GGCTTATAGGTTTAATGGTATTGG | GGCCATGGTACATTTGGCTAGG | GGCTGGCAATGGCGG |
| >MT253701 | GGCTTTGGAGACTCCGTGGAGGAGG | GGTAATAAAGGAGCTGGTGG | GGTATGTGGAAAGGTTATGG | GGTTGGACCTTTGGTGCAGG | GGCTTATAGGTTTAATGGTATTGG | GGCCATGGTACATTTGGCTAGG | GGCTGGCAATGGCGG |
| >MT253703 | GGCTTTGGAGACTCCGTGGAGGAGG | GGTAATAAAGGAGCTGGTGG | GGTATGTGGAAAGGTTATGG | GGTTGGACCTTTGGTGCAGG | GGCTTATAGGTTTAATGGTATTGG | GGCCATGGTACATTTGGCTAGG | GGCTGGCAATGGCGG |
| >MT253705 | GGCTTTGGAGACTCCGTGGAGGAGG | GGTAATAAAGGAGCTGGTGG | GGTATGTGGAAAGGTTATGG | GGTTGGACCTTTGGTGCAGG | GGCTTATAGGTTTAATGGTATTGG | GGCCATGGTACATTTGGCTAGG | GGCTGGCAATGGCGG |
| >MT253710 | GGCTTTGGAGACTCCGTGGAGGAGG | GGTAATAAAGGAGCTGGTGG | GGTATGTGGAAAGGTTATGG | GGTTGGACCTTTGGTGCAGG | GGCTTATAGGTTTAATGGTATTGG | GGCCATGGTACATTTGGCTAGG | GGCTGGCAATGGCGG |
| >MT253707 | GGCTTTGGAGACTCCGTGGAGGAGG | GGTAATAAAGGAGCTGGTGG | GGTATGTGGAAAGGTTATGG | GGTTGGACCTTTGGTGCAGG | GGCTTATAGGTTTAATGGTATTGG | GGCCATGGTACATTTGGCTAGG | GGCTGGCAATGGCGG |
| >MT253696 | GGCTTTGGAGACTCCGTGGAGGAGG | GGTAATAAAGGAGCTGGTGG | GGTATGTGGAAAGGTTATGG | GGTTGGACCTTTGGTGCAGG | GGCTTATAGGTTTAATGGTATTGG | GGCCATGGTACATTTGGCTAGG | GGCTGGCAATGGCGG |
| >MT253697 | GGCTTTGGAGACTCCGTGGAGGAGG | GGTAATAAAGGAGCTGGTGG | GGTATGTGGAAAGGTTATGG | GGTTGGACCTTTGGTGCAGG | GGCTTATAGGTTTAATGGTATTGG | GGCCATGGTACATTTGGCTAGG | GGCTGGCAATGGCGG |
| >MT253699 | GGCTTTGGAGACTCCGTGGAGGAGG | GGTAATAAAGGAGCTGGTGG | GGTATGTGGAAAGGTTATGG | GGTTGGACCTTTGGTGCAGG | GGCTTATAGGTTTAATGGTATTGG | GGCCATGGTACATTTGGCTAGG | GGCTGGCAATGGCGG |
| >MT253700 | GGCTTTGGAGACTCCGTGGAGGAGG | GGTAATAAAGGAGCTGGTGG | GGTATGTGGAAAGGTTATGG | GGTTGGACCTTTGGTGCAGG | GGCTTATAGGTTTAATGGTATTGG | GGCCATGGTACATTTGGCTAGG | GGCTGGCAATGGCGG |
| >MT253708 | GGCTTTGGAGACTCCGTGGAGGAGG | GGTAATAAAGGAGCTGGTGG | GGTATGTGGAAAGGTTATGG | GGTTGGACCTTTGGTGCAGG | GGCTTATAGGTTTAATGGTATTGG | GGCCATGGTACATTTGGCTAGG | GGCTGGCAATGGCGG |
| >MT253709 | GGCTTTGGAGACTCCGTGGAGGAGG | GGTAATAAAGGAGCTGGTGG | GGTATGTGGAAAGGTTATGG | GGTTGGACCTTTGGTGCAGG | GGCTTATAGGTTTAATGGTATTGG | GGCCATGGTACATTTGGCTAGG | GGCTGGCAATGGCGG |
| >MT251980 | GGCTTTGGAGACTCCGTGGAGGAGG | GGTAATAAAGGAGCTGGTGG | GGTATGTGGAAAGGTTATGG | GGTTGGACCTTTGGTGCAGG | GGCTTATAGGTTTAATGGTATTGG | GGCCATGGTACATTTGGCTAGG | GGCTGGCAATGGCGG |
| >MT251979 | GGCTTTGGAGACTCCGTGGAGGAGG | GGTAATAAAGGAGCTGGTGG | GGTATGTGGAAAGGTTATGG | GGTTGGACCTTTGGTGCAGG | GGCTTATAGGTTTAATGGTATTGG | GGCCATGGTACATTTGGCTAGG | GGCTGGCAATGGCGG |
| >MT251978 | GGCTTTGGAGACTCCGTGGAGGAGG | GGTAATAAAGGAGCTGGTGG | GGTATGTGGAAAGGTTATGG | GGTTGGACCTTTGGTGCAGG | GGCTTATAGGTTTAATGGTATTGG | GGCCATGGTACATTTGGCTAGG | GGCTGGCAATGGCGG |
| >MT253698 | GGCTTTGGAGACTCCGTGGAGGAGG | GGTAATAAAGGAGCTGGTGG | GGTATGTGGAAAGGTTATGG | GGTTGGACCTTTGGTGCAGG | GGCTTATAGGTTTAATGGTATTGG | GGCCATGGTACATTTGGCTAGG | GGCTGGCAATGGCGG |
| >MT251974 | GGCTTTGGAGACTCCGTGGAGGAGG | GGTAATAAAGGAGCTGGTGG | GGTATGTGGAAAGGTTATGG | GGTTGGACCTTTGGTGCAGG | GGCTTATAGGTTTAATGGTATTGG | GGCCATGGTACATTTGGCTAGG | GGCTGGCAATGGCGG |
| >MT251975 | GGCTTTGGAGACTCCGTGGAGGAGG | GGTAATAAAGGAGCTGGTGG | GGTATGTGGAAAGGTTATGG | GGTTGGACCTTTGGTGCAGG | GGCTTATAGGTTTAATGGTATTGG | GGCCATGGTACATTTGGCTAGG | GGCTGGCAATGGCGG |
| >MT246473 | GGCTTTGGAGACTCCGTGGAGGAGG | GGTAATAAAGGAGCTGGTGG | GGTATGTGGAAAGGTTATGG | GGTTGGACCTTTGGTGCAGG | GGCTTATAGGTTTAATGGTATTGG | GGCCATGGTACATTTGGCTAGG | GGCTGGCAATGGCGG |
| >MT246475 | GGCTTTGGAGACTCCGTGGAGGAGG | GGTAATAAAGGAGCTGGTGG | GGTATGTGGAAAGGTTATGG | GGTTGGACCTTTGGTGCAGG | GGCTTATAGGTTTAATGGTATTGG | GGCCATGGTACATTTGGCTAGG | GGCTGGCAATGGCGG |
| >MT246474 | GACTTTGGAGACTCCGTGGAGGAGG | GGTAATAAAGGAGCTGGTGG | GGTATGTGGAAAGGTTATGG | GGTTGGACCTTTGGTGCAGG | GGCTTATAGGTTTAATGGTATTGG | GGCCATGGTACATTTGGCTAGG | GGCTGGCAATGGCGG |
| >MT246468 | GGCTTTGGAGACTCCGTGGAGGAGG | GGTAATAAAGGAGCTGGTGG | GGTATGTGGAAAGGTTATGG | GGTTGGACCTTTGGTGCAGG | GGCTTATAGGTTTAATGGTATTGG | GGCCATGGTACATTTGGCTAGG | GGCTGGCAATGGCGG |
| >MT246469 | GGCTTTGGAGACTCCGTGGAGGAGG | GGTAATAAAGGAGCTGGTGG | GGTATGTGGAAAGGTTATGG | GGTTGGACCTTTGGTGCAGG | GGCTTATAGGTTTAATGGTATTGG | GGCCATGGTACATTTGGCTAGG | GGCTGGCAATGGCGG |
| >MT246470 | GGCTTTGGAGACTCCGTGGAGGAGG | GGTAATAAAGGAGCTGGTGG | GGTATGTGGAAAGGTTATGG | GGTTGGACCTTTGGTGCAGG | GGCTTATAGGTTTAATGGTATTGG | GGCCATGGTACATTTGGCTAGG | GGCTGGCAATGGCGG |
| >MT246471 | GGCTTTGGAGACTCCGTGGAGGAGG | GGTAATAAAGGAGCTGGTGG | GGTATGTGGAAAGGTTATGG | GGTTGGACCTTTGGTGCAGG | GGCTTATAGGTTTAATGGTATTGG | GGCCATGGTACATTTGGCTAGG | GGCTGGCAATGGCGG |
| >MT246472 | GGCTTTGGAGACTCCGTGGAGGAGG | GGTAATAAAGGAGCTGGTGG | GGTATGTGGAAAGGTTATGG | GGTTGGACCTTTGGTGCAGG | GGCTTATAGGTTTAATGGTATTGG | GGCCATGGTACATTTGGCTAGG | GGCTGGCAATGGCGG |
| >MT246486 | GGCTTTGGAGACTCCGTGGAGGAGG | GGTAATAAAGGAGCTGGTGG | GGTATGTGGAAAGGTTATGG | GGTTGGACCTTTGGTGCAGG | GGCTTATAGGTTTAATGGTATTGG | GGCCATGGTACATTTGGCTAGG | GGCTGGCAATGGCGG |
| >MT246487 | GGCTTTGGAGACTCCGTGGAGGAGG | GGTAATAAAGGAGCTGGTGG | GGTATGTGGAAAGGTTATGG | GGTTGGACCTTTGGTGCAGG | GGCTTATAGGTTTAATGGTATTGG | GGCCATGGTACATTTGGCTAGG | GGCTGGCAATGGCGG |
| >MT246451 | GGCTTTGGAGACTCCGTGGAGGAGG | GGTAATAAAGGAGCTGGTGG | GGTATGTGGAAAGGTTATGG | GGTTGGACCTTTGGTGCAGG | GGCTTATAGGTTTAATGGTATTGG | GGCCATGGTACATTTGGCTAGG | GGCTGGCAATGGCGG |
| >MT246452 | GGCTTTGGAGACTCCGTGGAGGAGG | GGTAATAAAGGAGCTGGTGG | GGTATGTGGAAAGGTTATGG | GGTTGGACCTTTGGTGCAGG | GGCTTATAGGTTTAATGGTATTGG | GGCCATGGTACATTTGGCTAGG | GGCTGGCAATGGCGG |
| >MT246453 | GGCTTTGGAGACTCCGTGGAGGAGG | GGTAATAAAGGAGCTGGTGG | GGTATGTGGAAAGGTTATGG | GGTTGGACCTTTGGTGCAGG | GGCTTATAGGTTTAATGGTATTGG | GGCCATGGTACATTTGGCTAGG | GGCTGGCAATGGCGG |
| >MT246454 | GGCTTTGGAGACTCCGTGGAGGAGG | GGTAATAAAGGAGCTGGTGG | GGTATGTGGAAAGGTTATGG | GGTTGGACCTTTGGTGCAGG | GGCTTATAGGTTTAATGGTATTGG | GGCCATGGTACATTTGGCTAGG | GGCTGGCAATGGCGG |
| >MT246455 | GGCTTTGGAGACTCCGTGGAGGAGG | GGTAATAAAGGAGCTGGTGG | GGTATGTGGAAAGGTTATGG | GGTTGGACCTTTGGTGCAGG | GGCTTATAGGTTTAATGGTATTGG | GGCCATGGTACATTTGGCTAGG | GGCTGGCAATGGCGG |
| >MT246456 | GGCTTTGGAGACTCCGTGGAGGAGG | GGTAATAAAGGAGCTGGTGG | GGTATGTGGAAAGGTTATGG | GGTTGGACCTTTGGTGCAGG | GGCTTATAGGTTTAATGGTATTGG | GGCCATGGTACATTTGGCTAGG | GGCTGGCAATGGCGG |
| >MT246457 | GGCTTTGGAGACTCCGTGGAGGAGG | GGTAATAAAGGAGCTGGTGG | GGTATGTGGAAAGGTTATGG | GGTTGGACCTTTGGTGCAGG | GGCTTATAGGTTTAATGGTATTGG | GGCCATGGTACATTTGGCTAGG | GGCTGGCAATGGCGG |
| >MT246460 | GGCTTTGGAGACTCCGTGGAGGAGG | GGTAATAAAGGAGCTGGTGG | GGTATGTGGAAAGGTTATGG | GGTTGGACCTTTGGTGCAGG | GGCTTATAGGTTTAATGGTATTGG | GGCCATGGTACATTTGGCTAGG | GGCTGGCAATGGCGG |
| >MT246476 | GGCTTTGGAGACTCCGTGGAGGAGG | GGTAATAAAGGAGCTGGTGG | GGTATGTGGAAAGGTTATGG | GGTTGGACCTTTGGTGCAGG | GGCTTATAGGTTTAATGGTATTGG | GGCCATGGTACATTTGGCTAGG | GGCTGGCAATGGCGG |
| >MT246477 | GGCTTTGGAGACTCCGTGGAGGAGG | GGTAATAAAGGAGCTGGTGG | GGTATGTGGAAAGGTTATGG | GGTTGGACCTTTGGTGCAGG | GGCTTATAGGTTTAATGGTATTGG | GGCCATGGTACATTTGGCTAGG | GGCTGGCAATGGCGG |
| >MT246478 | GGCTTTGGAGACTCCGTGGAGGAGG | GGTAATAAAGGAGCTGGTGG | GGTATGTGGAAAGGTTATGG | GGTTGGACCTTTGGTGCAGG | GGCTTATAGGTTTAATGGTATTGG | GGCCATGGTACATTTGGCTAGG | GGCTGGCAATGGCGG |
| >MT246479 | GGCTTTGGAGACTCCGTGGAGGAGG | GGTAATAAAGGAGCTGGTGG | GGTATGTGGAAAGGTTATGG | GGTTGGACCTTTGGTGCAGG | GGCTTATAGGTTTAATGGTATTGG | GGCCATGGTACATTTGGCTAGG | GGCTGGCAATGGCGG |
| >MT246480 | GGCTTTGGAGACTCCGTGGAGGAGG | GGTAATAAAGGAGCTGGTGG | GGTATGTGGAAAGGTTATGG | GGTTGGACCTTTGGTGCAGG | GGCTTATAGGTTTAATGGTATTGG | GGCCATGGTACATTTGGCTAGG | GGCTGGCAATGGCGG |
| >MT246481 | GGCTTTGGAGACTCCGTGGAGGAGG | GGTAATAAAGGAGCTGGTGG | GGTATGTGGAAAGGTTATGG | GGTTGGACCTTTGGTGCAGG | GGCTTATAGGTTTAATGGTATTGG | GGCCATGGTACATTTGGCTAGG | GGCTGGCAATGGCGG |
| >MT246458 | GGCTTTGGAGACTCCGTGGAGGAGG | GGTAATAAAGGAGCTGGTGG | GGTATGTGGAAAGGTTATGG | GGTTGGACCTTTGGTGCAGG | GGCTTATAGGTTTAATGGTATTGG | GGCCATGGTACATTTGGCTAGG | GGCTGGCAATGGCGG |
| >MT246488 | GGCTTTGGAGACTCCGTGGAGGAGG | GGTAATAAAGGAGCTGGTGG | GGTATGTGGAAAGGTTATGG | GGTTGGACCTTTGGTGCAGG | GGCTTATAGGTTTAATGGTATTGG | GGCCATGGTACATTTGGCTAGG | GGCTGGCAATGGCGG |
| >MT246489 | GGCTTTGGAGACTCCGTGGAGGAGG | GGTAATAAAGGAGCTGGTGG | GGTATGTGGAAAGGTTATGG | GGTTGGACCTTTGGTGCAGG | GGCTTATAGGTTTAATGGTATTGG | GGCCATGGTACATTTGGCTAGG | GGCTGGCAATGGCGG |
| >MT246490 | GGCTTTGGAGACTCCGTGGAGGAGG | GGTAATAAAGGAGCTGGTGG | GGTATGTGGAAAGGTTATGG | GGTTGGACCTTTGGTGCAGG | GGCTTATAGGTTTAATGGTATTGG | GGCCATGGTACATTTGGCTAGG | GGCTGGCAATGGCGG |
| >MT246667 | GGCTTTGGAGACTCCGTGGAGGAGG | GGTAATAAAGGAGCTGGTGG | GGTATGTGGAAAGGTTATGG | GGTTGGACCTTTGGTGCAGG | GGCTTATAGGTTTAATGGTATTGG | GGCCATGGTACATTTGGCTAGG | GGCTGGCAATGGCGG |
| >MT246484 | GGCTTTGGAGACTCCGTGGAGGAGG | GGTAATAAAGGAGCTGGTGG | GGTATGTGGAAAGGTTATGG | GGTTGGACCTTTGGTGCAGG | GGCTTATAGGTTTAATGGTATTGG | GGCCATGGTACATTTGGCTAGG | GGCTGGCAATGGCGG |
| >MT246485 | GGCTTTGGAGACTCCGTGGAGGAGG | GGTAATAAAGGAGCTGGTGG | GGTATGTGGAAAGGTTATGG | GGTTGGACCTTTGGTGCAGG | GGCTTATAGGTTTAATGGTATTGG | GGCCATGGTACATTTGGCTAGG | GGCTGGCAATGGCGG |
| >MT246482 | GGCTTTGGAGACTCCGTGGAGGAGG | GGTAATAAAGGAGCTGGTGG | GGTATGTGGAAAGGTTATGG | GGTTGGACCTTTGGTGCAGG | GGCTTATAGGTTTAATGGTATTGG | GGCCATGGTACATTTGGCTAGG | GGCTGGCAATGGCGG |
| >MT246463 | GGCTTTGGAGACTCCGTGGAGGAGG | GGTAATAAAGGAGCTGGTGG | GGTATGTGGAAAGGTTATGG | GGTTGGACCTTTGGTGCAGG | GGCTTATAGGTTTAATGGTATTGG | GGCCATGGTACATTTGGCTAGG | GGCTGGCAATGGCGG |
| >MT246464 | GGCTTTGGAGACTCCGTGGAGGAGG | GGTAATAAAGGAGCTGGTGG | GGTATGTGGAAAGGTTATGG | GGTTGGACCTTTGGTGCAGG | GGCTTATAGGTTTAATGGTATTGG | GGCCATGGTACATTTGGCTAGG | GGCTGGCAATGGCGG |
| >MT246466 | GGCTTTGGAGACTCCGTGGAGGAGG | GGTAATAAAGGAGCTGGTGG | GGTATGTGGAAAGGTTATGG | GGTTGGACCTTTGGTGCAGG | GGCTTATAGGTTTAATGGTATTGG | GGCCATGGTACATTTGGCTAGG | GGCTGGCAATGGCGG |
| >MT246467 | GGCTTTGGAGACTCCGTGGAGGAGG | GGTAATAAAGGAGCTGGTGG | GGTATGTGGAAAGGTTATGG | GGTTGGACCTTTGGTGCAGG | GGCTTATAGGTTTAATGGTATTGG | GGCCATGGTACATTTGGCTAGG | GGCTGGCAATGGCGG |
| >MT233526 | GGCTTTGGAGACTCCGTGGAGGAGG | GGTAATAAAGGAGCTGGTGG | GGTATGTGGAAAGGTTATGG | GGTTGGACCTTTGGTGCAGG | GGCTTATAGGTTTAATGGTATTGG | GGCCATGGTACATTTGGCTAGG | GGCTGGCAATGGCGG |
| >MT246449 | GGCTTTGGAGACTCCGTGGAGGAGG | GGTAATAAAGGAGCTGGTGG | GGTATGTGGAAAGGTTATGG | GGTTGGACCTTTGGTGCAGG | GGCTTATAGGTTTAATGGTATTGG | GGCCATGGTACATTTGGCTAGG | GGCTGGCAATGGCGG |
| >MT246450 | GGCTTTGGAGACTCCGTGGAGGAGG | GGTAATAAAGGAGCTGGTGG | GGTATGTGGAAAGGTTATGG | GGTTGGACCTTTGGTGCAGG | GGCTTATAGGTTTAATGGTATTGG | GGCCATGGTACATTTGGCTAGG | GGCTGGCAATGGCGG |
| >MT246459 | GGCTTTGGAGACTCCGTGGAGGAGG | GGTAATAAAGGAGCTGGTGG | GGTATGTGGAAAGGTTATGG | GGTTGGACCTTTGGTGCAGG | GGCTTATAGGTTTAATGGTATTGG | GGCCATGGTACATTTGGCTAGG | GGCTGGCAATGGCGG |
| >MT246462 | GGCTTTGGAGACTCCGTGGAGGAGG | GGTAATAAAGGAGCTGGTGG | GGTATGTGGAAAGGTTATGG | GGTTGGACCTTTGGTGCAGG | GGCTTATAGGTTTAATGGTATTGG | GGCCATGGTACATTTGGCTAGG | GGCTGGCAATGGCGG |
| >MT246461 | GGCTTTGGAGACTCCGTGGAGGAGG | GGTAATAAAGGAGCTGGTGG | GGTATGTGGAAAGGTTATGG | GGTTGGACCTTTGGTGCAGG | GGCTTATAGGTTTAATGGTATTGG | GGCCATGGTACATTTGGCTAGG | GGCTGGCAATGGCGG |
| >MT240479 | GGCTTTGGAGACTCCGTGGAGGAGG | GGTAATAAAGGAGCTGGTGG | GGTATGTGGAAAGGTTATGG | GGTTGGACCTTTGGTGCAGG | GGCTTATAGGTTTAATGGTATTGG | GGCCATGGTACATTTGGCTAGG | GGCTGGCAATGGCGG |
| >MT233522 | GGCTTTGGAGACTCCGTGGAGGAGG | GGTAATAAAGGAGCTGGTGG | GGTATGTGGAAAGGTTATGG | GGTTGGACCTTTGGTGCAGG | GGCTTATAGGTTTAATGGTATTGG | GGCCATGGTACATTTGGCTAGG | GGCTGGCAATGGCGG |
| >MT233519 | GGCTTTGGAGACTCCGTGGAGGAGG | GGTAATAAAGGAGCTGGTGG | GGTATGTGGAAAGGTTATGG | GGTTGGACCTTTGGTGCAGG | GGCTTATAGGTTTAATGGTATTGG | GGCCATGGTACATTTGGCTAGG | GGCTGGCAATGGCGG |
| >MT233523 | GGCTTTGGAGACTCCGTGGAGGAGG | GGTAATAAAGGAGCTGGTGG | GGTATGTGGAAAGGTTATGG | GGTTGGACCTTTGGTGCAGG | GGCTTATAGGTTTAATGGTATTGG | GGCCATGGTACATTTGGCTAGG | GGCTGGCAATGGCGG |
| >MT226610 | GGCTTTGGAGACTCCGTGGAGGAGG | GGTAATAAAGGAGCTGGTGG | GGTATGTGGAAAGGTTATGG | GGTTGGACCTTTGGTGCAGG | GGCTTATAGGTTTAATGGTATTGG | GGCCATGGTACATTTGGCTAGG | GGCTGGCAATGGCGG |
| >MT198652 | GGCTTTGGAGACTCCGTGGAGGAGG | GGTAATAAAGGAGCTGGTGG | GGTATGTGGAAAGGTTATGG | GGTTGGACCTTTGGTGCAGG | GGCTTATAGGTTTAATGGTATTGG | GGCCATGGTACATTTGGCTAGG | GGCTGGCAATGGCGG |
| >MT192772 | GGCTTTGGAGACTCCGTGGAGGAGG | GGTAATAAAGGAGCTGGTGG | GGTATGTGGAAAGGTTATGG | GGTTGGACCTTTGGTGCAGG | GGCTTATAGGTTTAATGGTATTGG | GGCCATGGTACATTTGGCTAGG | GGCTGGCAATGGCGG |
| >MT192765 | GGCTTTGGAGACTCCGTGGAGGAGG | GGTAATAAAGGAGCTGGTGG | GGTATGTGGAAAGGTTATGG | GGTTGGACCTTTGGTGCAGG | GGCTTATAGGTTTAATGGTATTGG | GGCCATGGTACATTTGGCTAGG | GGCTGGCAATGGCGG |
| >MT192759 | GGCTTTGGAGACTCCGTGGAGGAGG | GGTAATAAAGGAGCTGGTGG | GGTATGTGGAAAGGTTATGG | GGTTGGACCTTTGGTGCAGG | GGCTTATAGGTTTAATGGTATTGG | GGCCATGGTACATTTGGCTAGG | GGCTGGCAATGGCGG |
| >MT192773 | GGCTTTGGAGACTCCGTGGAGGAGG | GGTAATAAAGGAGCTGGTGG | GGTATGTGGAAAGGTTATGG | GGTTGGACCTTTGGTGCAGG | GGCTTATAGGTTTAATGGTATTGG | GGCCATGGTACATTTGGCTAGG | GGCTGGCAATGGCGG |
| >MT188340 | GGCTTTGGAGACTCCGTGGAGGAGG | GGTAATAAAGGAGCTGGTGG | GGTATGTGGAAAGGTTATGG | GGTTGGACCTTTGGTGCAGG | GGCTTATAGGTTTAATGGTATTGG | GGCCATGGTACATTTGGCTAGG | GGCTGGCAATGGCGG |
| >MT188339 | GGCTTTGGAGACTCCGTGGAGGAGG | GGTAATAAAGGAGCTGGTGG | GGTATGTGGAAAGGTTATGG | GGTTGGACCTTTGGTGCAGG | GGCTTATAGGTTTAATGGTATTGG | GGCCATGGTACATTTGGCTAGG | GGCTGGCAATGGCGG |
| >MT188341 | GGCTTTGGAGACTCCGTGGAGGAGG | GGTAATAAAGGAGCTGGTGG | GGTATGTGGAAAGGTTATGG | GGTTGGACCTTTGGTGCAGG | GGCTTATAGGTTTAATGGTATTGG | GGCCATGGTACATTTGGCTAGG | GGCTGGCAATGGCGG |
| >MT184908 | GGCTTTGGAGACTCCGTGGAGGAGG | GGTAATAAAGGAGCTGGTGG | GGTATGTGGAAAGGTTATGG | GGTTGGACCTTTGGTGCAGG | GGCTTATAGGTTTAATGGTATTGG | GGCCATGGTACATTTGGCTAGG | GGCTGGCAATGGCGG |
| >MT184909 | GGCTTTGGAGACTCCGTGGAGGAGG | GGTAATAAAGGAGCTGGTGG | GGTATGTGGAAAGGTTATGG | GGTTGGACCTTTGGTGCAGG | GGCTTATAGGTTTAATGGTATTGG | GGCCATGGTACATTTGGCTAGG | GGCTGGCAATGGCGG |
| >MT184910 | GGCTTTGGAGACTCCGTGGAGGAGG | GGTAATAAAGGAGCTGGTGG | GGTATGTGGAAAGGTTATGG | GGTTGGACCTTTGGTGCAGG | GGCTTATAGGTTTAATGGTATTGG | GGCCATGGTACATTTGGCTAGG | GGCTGGCAATGGCGG |
| >MT184907 | GGCTTTGGAGACTCCGTGGAGGAGG | GGTAATAAAGGAGCTGGTGG | GGTATGTGGAAAGGTTATGG | GGTTGGACCTTTGGTGCAGG | GGCTTATAGGTTTAATGGTATTGG | GGCCATGGTACATTTGGCTAGG | GGCTGGCAATGGCGG |
| >MT184911 | GGCTTTGGAGACTCCGTGGAGGAGG | GGTAATAAAGGAGCTGGTGG | GGTATGTGGAAAGGTTATGG | GGTTGGACCTTTGGTGCAGG | GGCTTATAGGTTTAATGGTATTGG | GGCCATGGTACATTTGGCTAGG | GGCTGGCAATGGCGG |
| >MT184913 | GGCTTTGGAGACTCCGTGGAGGAGG | GGTAATAAAGGAGCTGGTGG | GGTATGTGGAAAGGTTATGG | GGTTGGACCTTTGGTGCAGG | GGCTTATAGGTTTAATGGTATTGG | GGCCATGGTACATTTGGCTAGG | GGCTGGCAATGGCAG |
| >MT184912 | GGCTTTGGAGACTCCGTGGAGGAGG | GGTAATAAAGGAGCTGGTGG | GGTATGTGGAAAGGTTATGG | GGTTGGACCTTTGGTGCAGG | GGCTTATAGGTTTAATGGTATTGG | GGCCATGGTACATTTGGCTAGG | GGCTGGCAATGGCGG |
| >MT163719 | GGCTTTGGAGACTCCGTGGAGGAGG | GGTAATAAAGGAGCTGGTGG | GGTATGTGGAAAGGTTATGG | GGTTGGACCTTTGGTGCAGG | GGCTTATAGGTTTAATGGTATTGG | GGCCATGGTACATTTGGCTAGG | GGCTGGCAATGGCGG |
| >MT163716 | GGCTTTGGAGACTCCGTGGAGGAGG | GGTAATAAAGGAGCTGGTGG | GGTATGTGGAAAGGTTATGG | GGTTGGACCTTTGGTGCAGG | GGCTTATAGGTTTAATGGTATTGG | GGCCATGGTACATTTGGCTAGG | GGCTGGCAATGGCGG |
| >MT163717 | GGCTTTGGAGACTCCGTGGAGGAGG | GGTAATAAAGGAGCTGGTGG | GGTATGTGGAAAGGTTATGG | GGTTGGACCTTTGGTGCAGG | GGCTTATAGGTTTAATGGTATTGG | GGCCATGGTACATTTGGCTAGG | GGCTGGCAATGGCGG |
| >MT163718 | GGCTTTGGAGACTCCGTGGAGGAGG | GGTAATAAAGGAGCTGGTGG | GGTATGTGGAAAGGTTATGG | GGTTGGACCTTTGGTGCAGG | GGCTTATAGGTTTAATGGTATTGG | GGCCATGGTACATTTGGCTAGG | GGCTGGCAATGGCGG |
| >MT159705 | GGCTTTGGAGACTCCGTGGAGGAGG | GGTAATAAAGGAGCTGGTGG | GGTATGTGGAAAGGTTATGG | GGTTGGACCTTTGGTGCAGG | GGCTTATAGGTTTAATGGTATTGG | GGCCATGGTACATTTGGCTAGG | GGCTGGCAATGGCGG |
| >MT159722 | GGCTTTGGAGACTCCGTGGAGGAGG | GGTAATAAAGGAGCTGGTGG | GGTATGTGGAAAGGTTATGG | GGTTGGACCTTTGGTGCAGG | GGCTTATAGGTTTAATGGTATTGG | GGCCATGGTACATTTGGCTAGG | GGCTGGCAATGGCGG |
| >MT159710 | GGCTTTGGAGACTCCGTGGAGGAGG | GGTAATAAAGGAGCTGGTGG | GGTATGTGGAAAGGTTATGG | GGTTGGACCTTTGGTGCAGG | GGCTTATAGGTTTAATGGTATTGG | GGCCATGGTACATTTGGCTAGG | GGCTGGCAATGGCGG |
| >MT159715 | GGCTTTGGAGACTCCGTGGAGGAGG | GGTAATAAAGGAGCTGGTGG | GGTATGTGGAAAGGTTATGG | GGTTGGACCTTTGGTGCAGG | GGCTTATAGGTTTAATGGTATTGG | GGCCATGGTACATTTGGCTAGG | GGCTGGCAATGGCGG |
| >MT159716 | GGCTTTGGAGACTCCGTGGAGGAGG | GGTAATAAAGGAGCTGGTGG | GGTATGTGGAAAGGTTATGG | GGTTGGACCTTTGGTGCAGG | GGCTTATAGGTTTAATGGTATTGG | GGCCATGGTACATTTGGCTAGG | GGCTGGCAATGGCGG |
| >MT159711 | GGCTTTGGAGACTCCGTGGAGGAGG | GGTAATAAAGGAGCTGGTGG | GGTATGTGGAAAGGTTATGG | GGTTGGACCTTTGGTGCAGG | GGCTTATAGGTTTAATGGTATTGG | GGCCATGGTACATTTGGCTAGG | GGCTGGCAATGGCGG |
| >MT159712 | GGCTTTGGAGACTCCGTGGAGGAGG | GGTAATAAAGGAGCTGGTGG | GGTATGTGGAAAGGTTATGG | GGTTGGACCTTTGGTGCAGG | GGCTTATAGGTTTAATGGTATTGG | GGCCATGGTACATTTGGCTAGG | GGCTGGCAATGGCGG |
| >MT159717 | GGCTTTGGAGACTCCGTGGAGGAGG | GGTAATAAAGGAGCTGGTGG | GGTATGTGGAAAGGTTATGG | GGTTGGACCTTTGGTGCAGG | GGCTTATAGGTTTAATGGTATTGG | GGCCATGGTACATTTGGCTAGG | GGCTGGCAATGGCGG |
| >MT159719 | GGCTTTGGAGACTCCGTGGAGGAGG | GGTAATAAAGGAGCTGGTGG | GGTATGTGGAAAGGTTATGG | GGTTGGACCTTTGGTGCAGG | GGCTTATAGGTTTAATGGTATTGG | GGCCATGGTACATTTGGCTAGG | GGCTGGCAATGGCGG |
| >MT159720 | GGCTTTGGAGACTCCGTGGAGGAGG | GGTAATAAAGGAGCTGGTGG | GGTATGTGGAAAGGTTATGG | GGTTGGACCTTTGGTGCAGG | GGCTTATAGGTTTAATGGTATTGG | GGCCATGGTACATTTGGCTAGG | GGCTGGCAATGGCGG |
| >MT159709 | GGCTTTGGAGACTCCGTGGAGGAGG | GGTAATAAAGGAGCTGGTGG | GGTATGTGGAAAGGTTATGG | GGTTGGACCTTTGGTGCAGG | GGCTTATAGGTTTAATGGTATTGG | GGCCATGGTACATTTGGCTAGG | GGCTGGCAATGGCGG |
| >MT121215 | GGCTTTGGAGACTCCGTGGAGGAGG | GGTAATAAAGGAGCTGGTGG | GGTATGTGGAAAGGTTATGG | GGTTGGACCTTTGGTGCAGG | GGCTTATAGGTTTAATGGTATTGG | GGCCATGGTACATTTGGCTAGG | GGCTGGCAATGGCGG |
| >MT159718 | GGCTTTGGAGACTCCGTGGAGGAGG | GGTAATAAAGGAGCTGGTGG | GGTATGTGGAAAGGTTATGG | GGTTGGACCTTTGGTGCAGG | GGCTTATAGGTTTAATGGTATTGG | GGCCATGGTACATTTGGCTAGG | GGCTGGCAATGGCGG |
| >MT159721 | GGCTTTGGAGACTCCGTGGAGGAGG | GGTAATAAAGGAGCTGGTGG | GGTATGTGGAAAGGTTATGG | GGTTGGACCTTTGGTGCAGG | GGCTTATAGGTTTAATGGTATTGG | GGCCATGGTACATTTGGCTAGG | GGCTGGCAATGGCGG |
| >MT159708 | GGCTTTGGAGACTCCGTGGAGGAGG | GGTAATAAAGGAGCTGGTGG | GGTATGTGGAAAGGTTATGG | GGTTGGACCTTTGGTGCAGG | GGCTTATAGGTTTAATGGTATTGG | GGCCATGGTACATTTGGCTAGG | GGCTGGCAATGGCGG |
| >MT159714 | GGCTTTGGAGACTCCGTGGAGGAGG | GGTAATAAAGGAGCTGGTGG | GGTATGTGGAAAGGTTATGG | GGTTGGACCTTTGGTGCAGG | GGCTTATAGGTTTAATGGTATTGG | GGCCATGGTACATTTGGCTAGG | GGCTGGCAATGGCGG |
| >MT159707 | GGCTTTGGAGACTCCGTGGAGGAGG | GGTAATAAAGGAGCTGGTGG | GGTATGTGGAAAGGTTATGG | GGTTGGACCTTTGGTGCAGG | GGCTTATAGGTTTAATGGTATTGG | GGCCATGGTACATTTGGCTAGG | GGCTGGCAATGGCGG |
| >MT066156 | GGCTTTGGAGACTCCGTGGAGGAGG | GGTAATAAAGGAGCTGGTGG | GGTATGTGGAAAGGTTATGG | GGTTGGACCTTTGGTGCAGG | GGCTTATAGGTTTAATGGTATTGG | GGCCATGGTACATTTGGCTAGG | GGCTGGCAATGGCGG |
| >MT159706 | GGCTTTGGAGACTCCGTGGAGGAGG | GGTAATAAAGGAGCTGGTGG | GGTATGTGGAAAGGTTATGG | GGTTGGACCTTTGGTGCAGG | GGCTTATAGGTTTAATGGTATTGG | GGCCATGGTACATTTGGCTAGG | GGCTGGCAATGGCGG |
| >MT159713 | GGCTTTGGAGACTCCGTGGAGGAGG | GGTAATAAAGGAGCTGGTGG | GGTATGTGGAAAGGTTATGG | GGTTGGACCTTTGGTGCAGG | GGCTTATAGGTTTAATGGTATTGG | GGCCATGGTACATTTGGCTAGG | GGCTGGCAATGGCGG |
| >MT012098 | GGCTTTGGAGACTCCGTGGAGGAGG | GGTAATAAAGGAGCTGGTGG | GGTATGTGGAAAGGTTATGG | GGTTGGACCTTTGGTGCAGG | GGCTTATAGGTTTAATGGTATTGG | GGCCATGGTACATTTGGCTAGG | GGCTGGCAATGGCGG |
| >MT050493 | GGCTTTGGAGACTCCGTGGAGGAGG | GGTAATAAAGGAGCTGGTGG | GGTATGTGGAAAGGTTATGG | GGTTGGACCTTTGGTGCAGG | GGCTTATAGGTTTAATGGTATTGG | GGCCATGGTACATTTGGCTAGG | GGCTGGCAATGGCGG |
| >MT152824 | GGCTTTGGAGACTCCGTGGAGGAGG | GGTAATAAAGGAGCTGGTGG | GGTATGTGGAAAGGTTATGG | GGTTGGACCTTTGGTGCAGG | GGCTTATAGGTTTAATGGTATTGG | GGCCATGGTACATTTGGCTAGG | GGCTGGCAATGGCGG |
| >MT135041 | GGCTTTGGAGACTCCGTGGAGGAGG | GGTAATAAAGGAGCTGGTGG | GGTATGTGGAAAGGTTATGG | GGTTGGACCTTTGGTGCAGG | GGCTTATAGGTTTAATGGTATTGG | GGCCATGGTACATTTGGCTAGG | GGCTGGCAATGGCGG |
| >MT135042 | GGCTTTGGAGACTCCGTGGAGGAGG | GGTAATAAAGGAGCTGGTGG | GGTATGTGGAAAGGTTATGG | GGTTGGACCTTTGGTGCAGG | GGCTTATAGGTTTAATGGTATTGG | GGCCATGGTACATTTGGCTAGG | GGCTGGCAATGGCGG |
| >MT135043 | GGCTTTGGAGACTCCGTGGAGGAGG | GGTAATAAAGGAGCTGGTGG | GGTATGTGGAAAGGTTATGG | GGTTGGACCTTTGGTGCAGG | GGCTTATAGGTTTAATGGTATTGG | GGCCATGGTACATTTGGCTAGG | GGCTGGCAATGGCGG |
| >MT135044 | GGCTTTGGAGACTCCGTGGAGGAGG | GGTAATAAAGGAGCTGGTGG | GGTATGTGGAAAGGTTATGG | GGTTGGACCTTTGGTGCAGG | GGCTTATAGGTTTAATGGTATTGG | GGCCATGGTACATTTGGCTAGG | GGCTGGCAATGGCGG |
| >MT126808 | GGCTTTGGAGACTCCGTGGAGGAGG | GGTAATAAAGGAGCTGGTGG | GGTATGTGGAAAGGTTATGG | GGTTGGACCTTTGGTGCAGG | GGCTTATAGGTTTAATGGTATTGG | GGCCATGGTACATTTGGCTAGG | GGCTGGCAATGGCGG |
| >MT123292 | GGCTTTGGAGACTCCGTGGAGGAGG | GGTAATAAAGGAGCTGGTGG | GGTATGTGGAAAGGTTATGG | GGTTGGACCTTTGGTGCAGG | GGCTTATAGGTTTAATGGTATTGG | GGCCATGGTACATTTGGCTAGG | GGCTGGCAATGGCGG |
| >MT123290 | GGCTTTGGAGACTCCGTGGAGGAGG | GGTAATAAAGGAGCTGGTGG | GGTATGTGGAAAGGTTATGG | GGTTGGACCTTTGGTGCAGG | GGCTTATAGGTTTAATGGTATTGG | GGCCATGGTACATTTGGCTAGG | GGCTGGCAATGGCGG |
| >MT123291 | GGCTTTGGAGACTCCGTGGAGGAGG | GGTAATAAAGGAGCTGGTGG | GGTATGTGGAAAGGTTATGG | GGTTGGACCTTTGGTGCAGG | GGCTTATAGGTTTAATGGTATTGG | GGCCATGGTACATTTGGCTAGG | GGCTGGCAATGGCGG |
| >MT123293 | GGCTTTGGAGACTCCGTGGAGGAGG | GGTAATAAAGAAGCTGGTGG | GGTATGTGGAAAGGTTATGG | GGTTGGACCTTTGGTGCAGG | GGCTTATAGGTTTAATGGTATTGG | GGCCATGGTACATTTGGCTAGG | GGCTGGCAATGGCGG |
| >MT118835 | GGCTTTGGAGACTCCGTGGAGGAGG | GGTAATAAAGGAGCTGGTGG | GGTATGTGGAAAGGTTATGG | GGTTGGACCTTTGGTGCAGG | GGCTTATAGGTTTAATGGTATTGG | GGCCATGGTACATTTGGCTAGG | GGCTGGCAATGGCGG |
| >MT106053 | GGCTTTGGAGACTCCGTGGAGGAGG | GGTAATAAAGGAGCTGGTGG | GGTATGTGGAAAGGTTATGG | GGTTGGACCTTTGGTGCAGG | GGCTTATAGGTTTAATGGTATTGG | GGCCATGGTACATTTGGCTAGG | GGCTGGCAATGGCGG |
| >MT106052 | GGCTTTGGAGACTCCGTGGAGGAGG | GGTAATAAAGGAGCTGGTGG | GGTATGTGGAAAGGTTATGG | GGTTGGACCTTTGGTGCAGG | GGCTTATAGGTTTAATGGTATTGG | GGCCATGGTACATTTGGCTAGG | GGCTGGCAATGGCGG |
| >MT106054 | GGCTTTGGAGACTCCGTGGAGGAGG | GGTAATAAAGGAGCTGGTGG | GGTATGTGGAAAGGTTATGG | GGTTGGACCTTTGGTGCAGG | GGCTTATAGGTTTAATGGTATTGG | GGCCATGGTACATTTGGCTAGG | GGCTGGCAATGGCGG |
| >MT093631 | GGCTTTGGAGACTCCGTGGAGGAGG | GGTAATAAAGGAGCTGGTGG | GGTATGTGGAAAGGTTATGG | GGTTGGACCTTTGGTGCAGG | GGCTTATAGGTTTAATGGTATTGG | GGCCATGGTACATTTGGCTAGG | GGCTGGCAATGGCGG |
| >MT093571 | GGCTTTGGAGACTCCGTGGAGGAGG | GGTAATAAAGGAGCTGGTGG | GGTATGTGGAAAGGTTATGG | GGTTGGACCTTTGGTGCAGG | GGCTTATAGGTTTAATGGTATTGG | GGCCATGGTACATTTGGCTAGG | GGCTGGCAATGGCGG |
| >MT072688 | GGCTTTGGAGACTCCGTGGAGGAGG | GGTAATAAAGGAGCTGGTGG | GGTATGTGGAAAGGTTATGG | GGTTGGACCTTTGGTGCAGG | GGCTTATAGGTTTAATGGTATTGG | GGCCATGGTACATTTGGCTAGG | GGCTGGCAATGGCGG |
| >MT066175 | GGCTTTGGAGACTCCGTGGAGGAGG | GGTAATAAAGGAGCTGGTGG | GGTATGTGGAAAGGTTATGG | GGTTGGACCTTTGGTGCAGG | GGCTTATAGGTTTAATGGTATTGG | GGCCATGGTACATTTGGCTAGG | GGCTGGCAATGGCGG |
| >MT066176 | GGCTTTGGAGACTCCGTGGAGGAGG | GGTAATAAAGGAGCTGGTGG | GGTATGTGGAAAGGTTATGG | GGTTGGACCTTTGGTGCAGG | GGCTTATAGGTTTAATGGTATTGG | GGCCATGGTACATTTGGCTAGG | GGCTGGCAATGGCGG |
| >MT049951 | GGCTTTGGAGACTCCGTGGAGGAGG | GGTAATAAAGGAGCTGGTGG | GGTATGTGGAAAGGTTATGG | GGTTGGACCTTTGGTGCAGG | GGCTTATAGGTTTAATGGTATTGG | GGCCATGGTACATTTGGCTAGG | GGCTGGCAATGGCGG |
| >MT044257 | GGCTTTGGAGACTCCGTGGAGGAGG | GGTAATAAAGGAGCTGGTGG | GGTATGTGGAAAGGTTATGG | GGTTGGACCTTTGGTGCAGG | GGCTTATAGGTTTAATGGTATTGG | GGCCATGGTACATTTGGCTAGG | GGCTGGCAATGGCGG |
| >MT044258 | GGCTTTGGAGACTCCGTGGAGGAGG | GGTAATAAAGGAGCTGGTGG | GGTATGTGGAAAGGTTATGG | GGTTGGACCTTTGGTGCAGG | GGCTTATAGGTTTAATGGTATTGG | GGCCATGGTACATTTGGCTAGG | GGCTGGCAATGGCGG |
| >MT039890 | GGCTTTGGAGACTCCGTGGAGGAGG | GGTAATAAAGGAGCTGGTGG | GGTATGTGGAAAGGTTATGG | GGTTGGACCTTTGGTGCAGG | GGCTTATAGGTTTAATGGTATTGG | GGCCATGGTACATTTGGCTAGG | GGCTGGCAATGGCGG |
| >MT039887 | GGCTTTGGAGACTCCGTGGAGGAGG | GGTAATAAAGGAGCTGGTGG | GGTATGTGGAAAGGTTATGG | GGTTGGACCTTTGGTGCAGG | GGCTTATAGGTTTAATGGTATTGG | GGCCATGGTACATTTGGCTAGG | GGCTGGCAATGGCGG |
| >MT039873 | GGCTTTGGAGACTCCGTGGAGGAGG | GGTAATAAAGGAGCTGGTGG | GGTATGTGGAAAGGTTATGG | GGTTGGACCTTTGGTGCAGG | GGCTTATAGGTTTAATGGTATTGG | GGCCATGGTACATTTGGCTAGG | GGCTGGCAATGGCGG |
| >MT039888 | GGCTTTGGAGACTCCGTGGAGGAGG | GGTAATAAAGGAGCTGGTGG | GGTATGTGGAAAGGTTATGG | GGTTGGACCTTTGGTGCAGG | GGCTTATAGGTTTAATGGTATTGG | GGCCATGGTACATTTGGCTAGG | GGCTGGCAATGGCGG |
| >MT027063 | GGCTTTGGAGACTCCGTGGAGGAGG | GGTAATAAAGGAGCTGGTGG | GGTATGTGGAAAGGTTATGG | GGTTGGACCTTTGGTGCAGG | GGCTTATAGGTTTAATGGTATTGG | GGCCATGGTACATTTGGCTAGG | GGCTGGCAATGGCGG |
| >MT027064 | GGCTTTGGAGACTCCGTGGAGGAGG | GGTAATAAAGGAGCTGGTGG | GGTATGTGGAAAGGTTATGG | GGTTGGACCTTTGGTGCAGG | GGCTTATAGGTTTAATGGTATTGG | GGCCATGGTACATTTGGCTAGG | GGCTGGCAATGGCGG |
| >MT027062 | GGCTTTGGAGACTCCGTGGAGGAGG | GGTAATAAAGGAGCTGGTGG | GGTATGTGGAAAGGTTATGG | GGTTGGACCTTTGGTGCAGG | GGCTTATAGGTTTAATGGTATTGG | GGCCATGGTACATTTGGCTAGG | GGCTGGCAATGGCGG |
| >MT020881 | GGCTTTGGAGACTCCGTGGAGGAGG | GGTAATAAAGGAGCTGGTGG | GGTATGTGGAAAGGTTATGG | GGTTGGACCTTTGGTGCAGG | GGCTTATAGGTTTAATGGTATTGG | GGCCATGGTACATTTGGCTAGG | GGCTGGCAATGGCGG |
| >MT019533 | GGCTTTGGAGACTCCGTGGAGGAGG | GGTAATAAAGGAGCTGGTGG | GGTATGTGGAAAGGTTATGG | GGTTGGACCTTTGGTGCAGG | GGCTTATAGGTTTAATGGTATTGG | GGCCATGGTACATTTGGCTAGG | GGCTGGCAATGGCGG |
| >MT019531 | GGCTTTGGAGACTCCGTGGAGGAGG | GGTAATAAAGGAGCTGGTGG | GGTATGTGGAAAGGTTATGG | GGTTGGACCTTTGGTGCAGG | GGCTTATAGGTTTAATGGTATTGG | GGCCATGGTACATTTGGCTAGG | GGCTGGCAATGGCGG |
| >MT019532 | GGCTTTGGAGACTCCGTGGAGGAGG | GGTAATAAAGGAGCTGGTGG | GGTATGTGGAAAGGTTATGG | GGTTGGACCTTTGGTGCAGG | GGCTTATAGGTTTAATGGTATTGG | GGCCATGGTACATTTGGCTAGG | GGCTGGCAATGGCGG |
| >MT019530 | GGCTTTGGAGACTCCGTGGAGGAGG | GGTAATAAAGGAGCTGGTGG | GGTATGTGGAAAGGTTATGG | GGTTGGACCTTTGGTGCAGG | GGCTTATAGGTTTAATGGTATTGG | GGCCATGGTACATTTGGCTAGG | GGCTGGCAATGGCGG |
| >MT019529 | GGCTTTGGAGACTCCGTGGAGGAGG | GGTAATAAAGGAGCTGGTGG | GGTATGTGGAAAGGTTATGG | GGTTGGACCTTTGGTGCAGG | GGCTTATAGGTTTAATGGTATTGG | GGCCATGGTACATTTGGCTAGG | GGCTGGCAATGGCGG |
| >MT020880 | GGCTTTGGAGACTCCGTGGAGGAGG | GGTAATAAAGGAGCTGGTGG | GGTATGTGGAAAGGTTATGG | GGTTGGACCTTTGGTGCAGG | GGCTTATAGGTTTAATGGTATTGG | GGCCATGGTACATTTGGCTAGG | GGCTGGCAATGGCGG |
| >MT007544 | GGCTTTGGAGACTCCGTGGAGGAGG | GGTAATAAAGGAGCTGGTGG | GGTATGTGGAAAGGTTATGG | GGTTGGACCTTTGGTGCAGG | GGCTTATAGGTTTAATGGTATTGG | GGCCATGGTACATTTGGCTAGG | GGCTGGCAATGGCGG |
| >MN996527 | GGCTTTGGAGACTCCGTGGAGGAGG | GGTAATAAAGGAGCTGGTGG | GGTATGTGGAAAGGTTATGG | GGTTGGACCTTTGGTGCAGG | GGCTTATAGGTTTAATGGTATTGG | GGCCATGGTACATTTGGCTAGG | GGCTGGCAATGGCGG |
| >MN996528 | GGCTTTGGAGACTCCGTGGAGGAGG | GGTAATAAAGGAGCTGGTGG | GGTATGTGGAAAGGTTATGG | GGTTGGACCTTTGGTGCAGG | GGCTTATAGGTTTAATGGTATTGG | GGCCATGGTACATTTGGCTAGG | GGCTGGCAATGGCGG |
| >MN996529 | GGCTTTGGAGACTCCGTGGAGGAGG | GGTAATAAAGGAGCTGGTGG | GGTATGTGGAAAGGTTATGG | GGTTGGACCTTTGGTGCAGG | GGCTTATAGGTTTAATGGTATTGG | GGCCATGGTACATTTGGCTAGG | GGCTGGCAATGGCGG |
| >MN996530 | GGCTTTGGAGACTCCGTGGAGGAGG | GGTAATAAAGGAGCTGGTGG | GGTATGTGGAAAGGTTATGG | GGTTGGACCTTTGGTGCAGG | GGCTTATAGGTTTAATGGTATTGG | GGCCATGGTACATTTGGCTAGG | GGCTGGCAATGGCGG |
| >MN996531 | GGCTTTGGAGACTCCGTGGAGGAGG | GGTAATAAAGGAGCTGGTGG | GGTATGTGGAAAGGTTATGG | GGTTGGACCTTTGGTGCAGG | GGCTTATAGGTTTAATGGTATTGG | GGCCATGGTACATTTGGCTAGG | GGCTGGCAATGGCGG |
| >MN988669 | GGCTTTGGAGACTCCGTGGAGGAGG | GGTAATAAAGGAGCTGGTGG | GGTATGTGGAAAGGTTATGG | GGTTGGACCTTTGGTGCAGG | GGCTTATAGGTTTAATGGTATTGG | GGCCATGGTACATTTGGCTAGG | GGCTGGCAATGGCGG |
| >MN994467 | GGCTTTGGAGACTCCGTGGAGGAGG | GGTAATAAAGGAGCTGGTGG | GGTATGTGGAAAGGTTATGG | GGTTGGACCTTTGGTGCAGG | GGCTTATAGGTTTAATGGTATTGG | GGCCATGGTACATTTGGCTAGG | GGCTGGCAATGGCGG |
| >MN994468 | GGCTTTGGAGACTCCGTGGAGGAGG | GGTAATAAAGGAGCTGGTGG | GGTATGTGGAAAGGTTATGG | GGTTGGACCTTTGGTGCAGG | GGCTTATAGGTTTAATGGTATTGG | GGCCATGGTACATTTGGCTAGG | GGCTGGCAATGGCGG |
| >MN997409 | GGCTTTGGAGACTCCGTGGAGGAGG | GGTAATAAAGGAGCTGGTGG | GGTATGTGGAAAGGTTATGG | GGTTGGACCTTTGGTGCAGG | GGCTTATAGGTTTAATGGTATTGG | GGCCATGGTACATTTGGCTAGG | GGCTGGCAATGGCGG |
| >MN988668 | GGCTTTGGAGACTCCGTGGAGGAGG | GGTAATAAAGGAGCTGGTGG | GGTATGTGGAAAGGTTATGG | GGTTGGACCTTTGGTGCAGG | GGCTTATAGGTTTAATGGTATTGG | GGCCATGGTACATTTGGCTAGG | GGCTGGCAATGGCGG |
| >MN988713 | GGCTTTGGAGACTCCGTGGAGGAGG | GGTAATAAAGGAGCTGGTGG | GGTATGTGGAAAGGTTATGG | GGTTGGACCTTTGGTGCAGG | GGCTTATAGGTTTAATGGTATTGG | GGCCATGGTACATTTGGCTAGG | GGCTGGCAATGGCGG |
| >MN975262 | GGCTTTGGAGACTCCGTGGAGGAGG | GGTAATAAAGGAGCTGGTGG | GGTATGTGGAAAGGTTATGG | GGTTGGACCTTTGGTGCAGG | GGCTTATAGGTTTAATGGTATTGG | GGCCATGGTACATTTGGCTAGG | GGCTGGCAATGGCGG |
| >MN938384 | GGCTTTGGAGACTCCGTGGAGGAGG | GGTAATAAAGGAGCTGGTGG | GGTATGTGGAAAGGTTATGG | GGTTGGACCTTTGGTGCAGG | GGCTTATAGGTTTAATGGTATTGG | GGCCATGGTACATTTGGCTAGG | GGCTGGCAATGGCGG |
| >MN985325 | GGCTTTGGAGACTCCGTGGAGGAGG | GGTAATAAAGGAGCTGGTGG | GGTATGTGGAAAGGTTATGG | GGTTGGACCTTTGGTGCAGG | GGCTTATAGGTTTAATGGTATTGG | GGCCATGGTACATTTGGCTAGG | GGCTGGCAATGGCGG |
| >MN908947 | GGCTTTGGAGACTCCGTGGAGGAGG | GGTAATAAAGGAGCTGGTGG | GGTATGTGGAAAGGTTATGG | GGTTGGACCTTTGGTGCAGG | GGCTTATAGGTTTAATGGTATTGG | GGCCATGGTACATTTGGCTAGG | GGCTGGCAATGGCGG |
